# Integrated transcriptome profiling of plasma exosomes reveals molecular stratification of exocrine and endocrine disorders and S100A8-mediated cell interactions in chronic pancreatitis

**DOI:** 10.1038/s41421-025-00832-x

**Published:** 2025-11-18

**Authors:** Deyu Zhang, Zaoqu Liu, Shiyu Li, Shutong Liu, Wanshun Li, Hongxuan Ma, Liqi Sun, Lisi Peng, Mengruo Jiang, Zhenghui Yang, Chang Wu, Yue Liu, Jiayu Li, Zhendong Jin, Xinwei Han, Baoan Ji, Zhaoshen Li, Haojie Huang

**Affiliations:** 1 Department of Gastroenterology, Changhai Hospital, Naval Medical University, Shanghai, China; 2 Institute of Basic Medical Sciences, Chinese Academy of Medical Sciences and Peking Union Medical College, Beijing, China; 3 Department of Gastroenterology, Sir Run Run Shaw Hospital, Zhejiang University School of Medicine, Hangzhou, Zhejiang China; 4 Department of Interventional Radiology, The First Affiliated Hospital of Zhengzhou University, Zhengzhou, Henan China; 5 National Key Laboratory of Immunity and Inflammation, Institute of Immunology, Naval Medical University, Shanghai, China; 6 Department of Nephrology, The First Affiliated Hospital of Zhengzhou University, Zhengzhou, Henan China; 7 Department of Cancer Biology, Mayo Clinic, Jacksonville, FL USA

**Keywords:** Bioinformatics, miRNAs

## Abstract

Exocrine and endocrine disorders and insufficiency are two major harmful pathological processes in chronic pancreatitis (CP) and can lead to steatorrhea and diabetes. However, there is a lack of reliable clinical classification schemes for evaluating the severity of exocrine and endocrine disorders in CP, and the underlying mechanisms are also unclear. In particular, exosome-based liquid biopsy and classification in CP are lacking. Here, we performed transcriptome sequencing on plasma exosomes from CP patients with different degrees of CP severity. Additionally, we analyzed single-cell sequencing data from pancreatic lesions in CP patients to interpret the classification, and an external cohort was established to verify the classification. Ultimately, we established and preliminarily verified a 3-stage classification system to predict steatorrhea and diabetes onset in CP patients based on the expression of 12 miRNAs in plasma exosomes. A publicly-available online tool implementing this classification system was also developed. Further analysis, in combination with single-cell sequencing data from CP mice, identified exosome-derived miR-24-3p and neutrophil S100A8 as pivotal factors in CP progression. Mechanistically, our findings suggest that downregulated exosome-derived miR-24-3p in CP may lead to the upregulation of its target gene, S100A8, in neutrophils, thus promoting CP-related exocrine and endocrine disorders by activating the fibrotic phenotype of pancreatic stellate cells and inducing inflammation in macrophages, leading to the apoptosis of pancreatic β cells. Together, our work provides a novel exosome-based 3-stage classification system for CP and highlights the role of exosomal miR-24-3p and S100A8 in fibrosis and pancreatic β-cell apoptosis.

## Introduction

Chronic pancreatitis (CP) is a condition of gradually worsening fibrosis and inflammation that affects pancreatic tissue and is driven by a complex interaction of genetic and environmental factors^1^. CP is characterized by repeated inflammatory episodes that lead to the replacement of the pancreatic parenchyma with fibrous tissue. This fibrosis results in a gradual decline in both exocrine and endocrine pancreatic function, leading to various complications, including pseudocyst formation, obstruction of the pancreatic and bile ducts, steatorrhea, diabetes, vascular issues, malnutrition, and persistent pain. Among these complications, steatorrhea and diabetes are particularly serious. Steatorrhea is a major sign of severe pancreatic exocrine insufficiency and usually presents with nutritional malabsorption, which leads to vitamin and micronutrient deficiency and weight loss and increases the risk of developing premature atherosclerosis, cardiovascular events, osteoporosis, fracture, immune deficiency, and infection^2–5^. Diabetes mellitus that appears after the development of CP is referred to as type 3c diabetes mellitus (T3cDM) and is a sign of endocrine dysfunction^2^. In addition to experiencing the serious complications that affect all diabetic patients, CP patients with T3cDM are particularly vulnerable to complications or death related to hypoglycemic events^6,7^. Thus, CP substantially diminishes both the quality of life and life expectancy of the affected patients.

Given the range of effects of CP, evaluating disease severity, monitoring treatment effects and determining the risk of severe complications are of particular importance in this disease. The first classification of CP, established at a symposium in Marseilles in 1963, emphasized morphological changes and disease etiology^8^. A revised version in 1985 refined these morphological criteria and linked them to potential losses in pancreatic function, introducing further subtypes in the Marseilles–Rome classification^9,10^. The Cambridge classification, the first clinical grading system for CP, is based on ductal changes observed in endoscopic retrograde pancreatography (ERP) and, to some extent, on ultrasound or CT findings^11,12^. Although the Cambridge criteria are the standard for imaging-based grading of CP, experts have noted that these morphological changes might not accurately reflect the functional or histological condition of the pancreas, potentially missing early disease stages^13^. More recently, several classification systems have been developed to describe disease progression by grading severity on the basis of imaging, clinical symptoms, laboratory indices, the need for intervention, and functional loss^14–18^. However, these systems are designed to evaluate the degree of CP severity on the basis of only a clinical index rather than a combination of molecular biology features, compromising the clinical accuracy of these classifications.

Molecular insights gained over the past 20 years have demonstrated the heterogeneous nature of CP. Notably, several genetic mutations have been found to contribute to disease pathogenesis by disrupting trypsin regulation, impairing anti-proteolytic defenses, or altering calcium signaling. For example, *PRSS1* gain-of-function mutations are hallmark drivers of hereditary CP^19^, whereas CASR polymorphisms are strongly associated with alcohol-related subtypes^20^. On the basis of these findings, recent studies have further proposed molecular subtyping strategies for categorizing CP into genetic, alcohol-associated, and idiopathic subtypes^1^. Nevertheless, these classification systems remain largely etiology-based, whereas the systematic integration of multi-omics biomarkers would permit precision stratification and assessment of the current risk of complications, especially steatorrhea and diabetes. Moreover, liquid biopsy techniques based on novel markers and high-throughput sequencing were not applied in these systems. Additionally, the basic mechanisms associated with different levels of disease severity are not well defined.

Extracellular vesicles (exosomes) are particles characterized by a lipid bilayer and a typical size of 50 nm to 1 µm; they mainly contain proteins and RNAs, including mRNAs, long noncoding RNAs (lncRNAs), and miRNAs, as cargo. Several previous studies have indicated that exosomes produced by pancreatic parenchymal cells are key mediators of pancreatic cell communication^21^ and that exosomes play a role in fibrosis^22,23^ and exocrine and endocrine impairment^24^ in CP. However, the potential of peripheral exosomes as sources of clinically relevant RNA and protein biomarkers remains largely unexplored.

In this study, we preliminarily confirmed that specific proteins and RNAs released by damaged pancreatic parenchymal cells in the CP are encapsulated by and can be identified in circulating exosomes. These exosomes allow the transcriptomic and proteomic characterization of various types of damaged pancreatic parenchymal cells in CP. This approach shows potential for the development of an AI-based stratification system to assess disease severity and complication risk, as well as for elucidating the mechanisms underlying CP-related exocrine and endocrine dysfunction.

## Results

### Whole-transcriptome sequencing reveals differentially expressed exosome-derived RNAs in CP patients and healthy donors

**Fig. 1:**
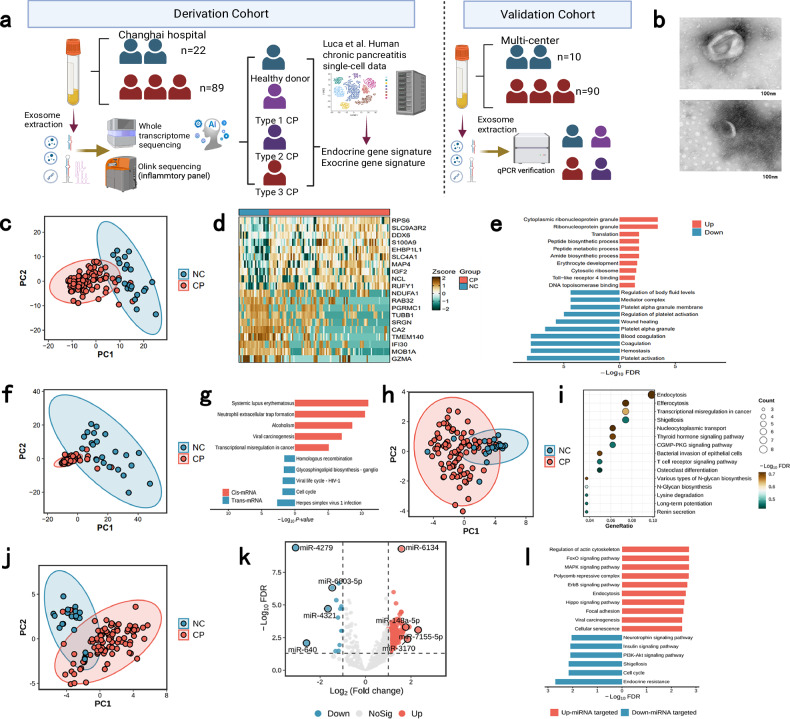
Characterization of differentially expressed exosome-derived RNAs through whole-transcriptome sequencing. **a** Flow chart of this study. **b** A representative electron microscope image of extracted exosomes. **c** Principal component analysis (PCA) of the differences in mRNA expression between the healthy donor (NC) group and CP groups. **d** Top 10 differentially expressed mRNAs (DEMs) between the NC and CP groups (FDR < 0.05). **e** Top 10 enriched pathways associated with the up- or down-regulated mRNAs between the NC and CP groups (FDR < 0.05). **f** PCA of lncRNA expression in the NC and CP groups. **g** Top 10 pathways enriched among the lncRNA-related DEGs (*cis*-mRNAs and *trans*-mRNAs) between the NC and CP groups (FDR < 0.05). **h** PCA of circRNA expression between the NC and CP groups. **i** GO terms enriched among the host genes of the differentially expressed circRNAs (FDR < 0.05). **j** PCA plot of circRNA expression in the NC and CP groups. **k** Volcano plot of DEmiRNAs. **l** The pathways enriched among the genes targeted by the differentially expressed miRNAs (FDR < 0.05).

A flow chart of the study design is shown in Fig. 1a. First, plasma was collected from 89 CP patients and 22 healthy donors (NCs), and exosomes were extracted and verified (Fig. 1b; Supplementary Table S1 and Fig. S1a, b). Then, the RNA contents of the exosomes were sequenced, and differential expression analysis of the mRNAs, miRNAs, lncRNAs, and circular RNAs (circRNAs) between the patient and healthy groups was performed, followed by pathway enrichment analysis. All of the types of RNA exhibited distinct distributions between CP patients and NCs (Fig. 1c, f, h, j). The differentially expressed genes (DEGs) were identified according to the criteria of |logFold Change (FC) > 1| and false discovery rate (FDR) < 0.05 and visualized (Fig. 1d, k; Supplementary Figs. S1c, e, f, S2a, b, f and Tables S2–S5). The pathways enriched among the DEGs indicate the promotion of translation-related pathways and keratinocyte migration (Fig. 1e; Supplementary Fig. S1d). The pathways enriched among putative *cis*- and *trans*-regulatory mRNAs of the differentially expressed lncRNAs (DElncRNAs) were related to autism and neutrophil activation (Fig. 1g), and Gene Ontology (GO) analysis suggested activation of DNA replication pathways (Supplementary Fig. S1g, h). The putative *cis*- and *trans*-regulatory mRNAs of the DElncRNAs in these pathways were also visualized (Supplementary Fig. S1i–k).

Next, the host genes of the differentially expressed circRNAs were identified. The enriched GO terms were related to endocytosis (Fig. 1i; Supplementary Fig. S2d, e). The enriched pathway terms were related to protein processes (Supplementary Fig. S2c). Additionally, pathway enrichment analysis revealed that the differentially expressed miRNA-targeted genes are related to the activation of several specific pathways, including the FoxO, MAPK, Erbb, and Hippo pathways, endocrine resistance, and the insulin signaling pathway (Fig. 1l; Supplementary Fig. S2h–k). GO analysis also revealed enrichment of protein process-related terms (Fig. 1g).

### Establishment and validation of a three-level classification complication prediction system

**Fig. 2:**
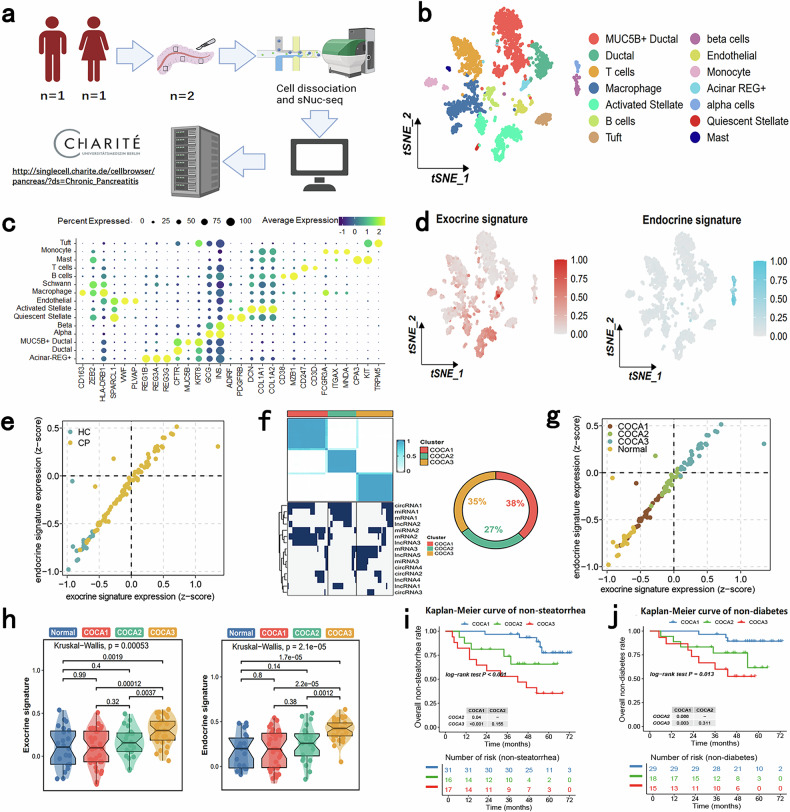
Establishment and validation of a three-level classification system based on exosomes from CP patients in the derivation cohort. **a** Flow diagram of the acquisition of online human CP single-cell sequencing data. **b** Uniform manifold approximation and projection (UMAP) of cell types identified in human CP single-cell sequencing data. **c** Dot plot of marker genes in each cell type. **d** UMAP plot of the expression of exocrine or endocrine signatures. **e** Four-quadrant diagram showing the expression of exocrine or endocrine signature in each sample, divided into HCs or CP patients. **f** Left: concept map of the COCA classification method. Right: flow diagram of the acquisition of online human CP single-cell sequencing data. **g** Four-quadrant diagram showing the expression of exocrine or endocrine signatures in each sample, divided into HCs or each subtype of CP patients. **h** Expression of the exocrine or endocrine signature among HCs or each subtype of CP. **i** Kaplan–Meier curve of steatorrhea development in non-steatorrhea CP patients stratified by COCA subtype during follow-up in the derivation cohort. **j** Kaplan–Meier curve of diabetes development in non-diabetic CP patients stratified by COCA subtype during follow-up in the derivation cohort.

A previous study indicated that lesion-derived exosomes can disseminate into the peripheral blood and that liquid biopsy to measure these exosomes had value in prostate cancer patients^25^. Therefore, we speculate that RNAs and proteins from injured pancreatic parenchymal cells in CP might be encapsulated within exosomes and subsequently secreted into the bloodstream. Specifically, we hypothesized that the RNA contents of peripheral exosomes may reflect the severity of pancreatic parenchymal cell injury in CP patients and the risk of CP-related complications, including steatorrhea and diabetes. To test our hypothesis, we first reanalyzed single-cell sequencing data downloaded from a public database. A schematic of the online single-cell sequencing data is shown in Fig. 2a^26^. Annotation identified 13 cell types in the CP group (Fig. 2b; Supplementary Fig. S3a–c), and the expression levels of the corresponding marker genes were visualized (Fig. 2c). We subsequently identified and visualized the expression of two gene signatures in the single-cell sequencing data: one exocrine signature, consisting of the top 40 marker genes (based on logFC values) of acinar REG^+^ cells and activated stellate cells (Supplementary Table S6), and one endocrine signature, consisting of the top 40 marker genes (based on logFC values) of alpha cells and beta cells (Fig. 2d; Supplementary Table S7). The two signatures were mapped to our exosome sequencing data, and then the median expression levels of genes in each signature in CP patients were defined as the expression of each signature and visualized in a dot plot (Fig. 2e). Notably, both exocrine signature genes and endocrine signature genes were expressed at low levels in healthy donors and at higher levels in CP patients, consistent with our initial assumptions.

Next, to identify common subtypes based on the combinations of different types of RNAs in exosomes from the peripheral blood of CP patients, analysis with the cluster of cluster assignment (COCA) algorithm^27^ was applied to each of the four molecular types above (mRNA, miRNA, lncRNA, and circRNA). The cluster number was set to 3, as this produced the largest silhouette score and lowest PAC score (Supplementary Fig. S3d). The 3 clusters, COCA1–3, were identified as CP patient subtypes (Fig. 2f). The clinical characteristics of each cluster of CP patients are shown in Supplementary Table S8, indicating a trend of increasing CP severity, with increasing morbidity of diabetes mellitus and steatorrhea, from COCA1 to COCA3. Consistent with this finding, we also observed that the conversion of COCA subtypes was coincident with increased expression of the exocrine and endocrine signatures (Fig. 2g, h). PCA of the expression of genes in these two signatures in exosome sequencing data also revealed that the COCA1 cluster was closer to normal samples (healthy donors), whereas the COCA2 and COCA3 clusters were further away from normal samples (Supplementary Fig. S3e).

On the basis of the above findings, we sought to determine whether this COCA classification system could be used to predict the risk of diabetes and steatorrhea in CP patients. To this end, patients who did not have steatorrhea or diabetes at the time of sample collection were included for follow-up. Among these patients, we observed a significantly higher incidence of steatorrhea and diabetes in those with COCA3 or COCA2 subtypes compared with those with the COCA1 subtype (Fig. 2i, j, *P* < 0.05), suggesting that the COCA system we developed can predict the risk of incipient diabetes and steatorrhea in CP patients.

### Identification of the main exosome-related characteristics of each COCA subtype

**Fig. 3:**
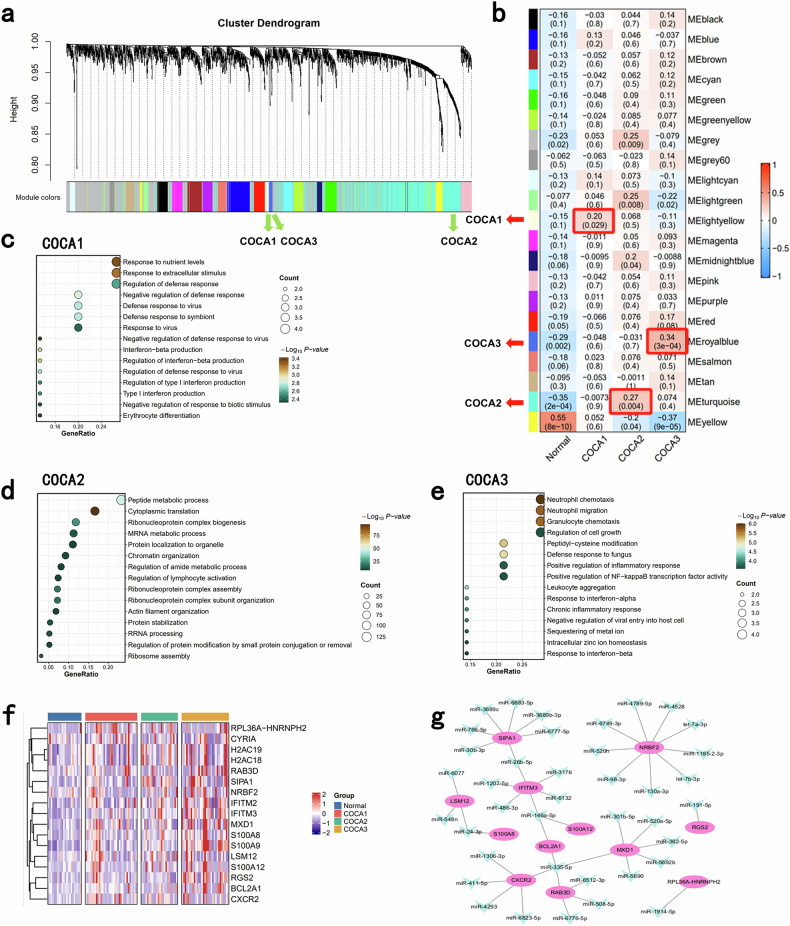
Identification of the main exosome-related characteristics of each COCA subtype. **a** Dendrogram of each identified cluster. **b** Correlations between each cluster and each COCA subtype. The first line in each box shows the correlation index, and the line below shows the FDR value. **c** The pathways significantly enriched in the MElightyellow module (COCA1). **d** The pathways significantly enriched in the MEturquoise module (COCA2). **e** The pathways significantly enriched in the MEroyalblue module (COCA3). **f** The expression of the top 17 genes of the MEroyalblue module in normal samples and each COCA subtype. **g** Prediction of miRNA‒mRNA sponging networks.

To define the key pathways activated in each COCA subtype on the basis of mRNA data from exosome sequencing, we performed weighted gene correlation network analysis (WGCNA) (Supplementary Fig. S4a–d). Eighteen distinct modules were identified (Fig. 3a), and correlation analysis revealed that the MElightyellow, MEturquoise, and MEroyalblue modules were significantly positively correlated with COCA1, COCA2, and COCA3 subtypes, respectively (Fig. 3b). Thus, these modules were identified as marker modules specific to each corresponding COCA subtype. The pathways enriched in the COCA1 subtype (MElightyellow module) were related to stress responses, including the defense response and extracellular stimulus (Fig. 3c). The pathways enriched in the COCA2 subtype (MEturquoise module) were related to metabolic changes, including peptide metabolic processes and mRNA metabolic processes (Fig. 3d). The pathways enriched in the COCA3 subtype (MEroyalblue module) were related to inflammation, including neutrophil-related migration and the chronic inflammatory response (Fig. 3e). The top 17 upregulated genes in MEroyalblue are shown in Fig. 3f, demonstrating a gradually increasing trend from the normal group to the COCA1 group and then the COCA3 group. Then, candidate miRNA–mRNA interaction networks within these groups, based on experimental evidence, were subsequently downloaded from the miRTarBase (Supplementary Table S9). The potential sponged significantly downregulated miRNAs of these genes are shown in Fig. 3g.

### A 12-miRNA diagnostic classifier accurately discriminates control, COCA1, COCA2, and COCA3 patients

**Fig. 4:**
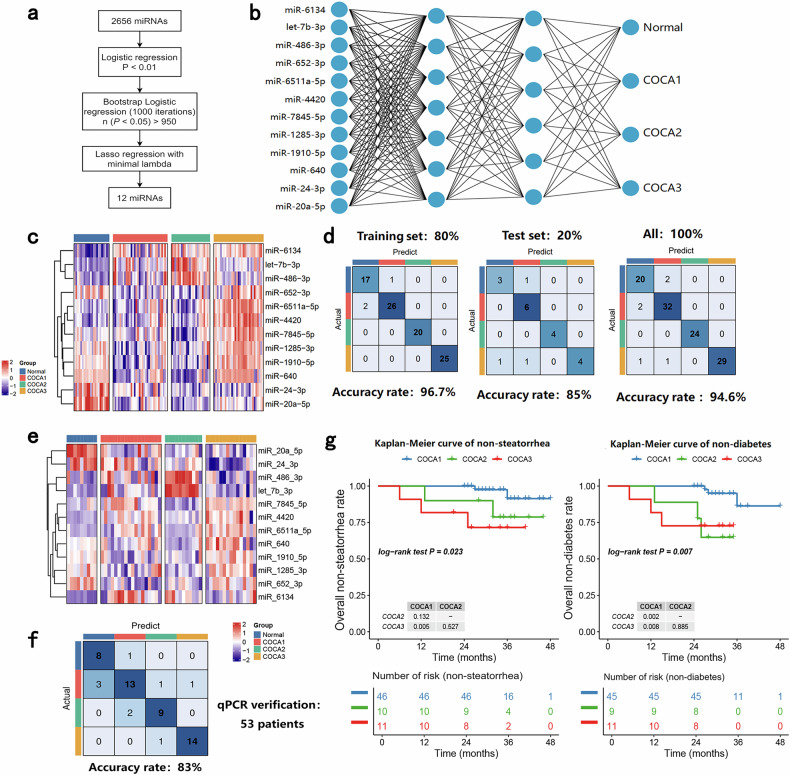
Development and validation of a 12-miRNA diagnostic classifier for COCA subtypes of CP in the derivation cohort and validation cohort. **a** LASSO regression analysis was performed on the basis of the expression of the exosome sequencing-derived miRNA matrix. **b** Structure diagram of the BPNN algorithm. **c** Expression heatmap of the 12 screened miRNAs. **d** Accuracy rates of the developed classifier in the training sets (left), test sets (middle), and all datasets (right). **e** Expression heatmap of 12 screened miRNAs in 53 randomly selected exosome samples determined via qPCR. **f** Accuracy rates of the developed classifier on the basis of the qPCR data from 53 randomly selected exosome samples. **g** Left: Kaplan–Meier curve of steatorrhea development in non-steatorrhea CP patients stratified by COCA subtype during follow-up in the derivation cohort. Right: Kaplan–Meier curve of diabetes development in non-diabetic CP patients stratified by COCA subtype during follow-up in the derivation cohort.

Although the above approach identified three subtypes of CP patients on the basis of the exosome sequencing data, there were still several methodological shortcomings. In particular, this classification algorithm was not able to distinguish healthy individuals from CP patients and also required comprehensive exosome sequencing data, rather than only a few markers, for precise classification. In addition, the clinical differences among the subtypes were not evaluated in the validation cohort. In light of previous work reporting that miRNAs are relatively stable in exosomes and are optimal diagnostic indices^28^, we focused on these RNAs to develop a more robust diagnostic. First, to address the challenges of high dimensionality and multicollinearity inherent in exosome miRNA sequencing data, we implemented LASSO regression with L1 regularization, a method proven effective for biomarker selection in sparse, correlated biological datasets^29^. LASSO regression-derived biomarkers have remarkable clinical translatability and could enable precise disease subtype stratification when integrated with BPNN’s nonlinear modeling capabilities—a synergistic approach that not only reduces computational complexity but also enhances the biological interpretability of the diagnostic framework. Therefore, we established a 12-miRNA diagnostic classifier using the LASSO + BPNN algorithm on the basis of miRNA sequencing data from existing COCA subtypes and normal samples (Fig. 4a). The expression of the 12 miRNAs is shown in Fig. 4b, and the structure of the BPNN is visualized in Fig. 4c. The sequencing data were then randomly divided into a training set (80% of the patients) and a test set (20% of the patients). The efficacy of the classifier was then tested on the training sets, test sets and their combination (all patients), yielding accuracy values of 85–96.7% (Fig. 4d). We then randomly selected 53 samples from all the exosome sequencing samples for quantitative polymerase chain reaction (qPCR) of these 12 miRNAs (Fig. 4e); the qPCR results were then input into the classifier, yielding an accuracy of 83% (Fig. 4f). Next, we performed PCR on plasma exosome samples from healthy controls (*n* = 10) and CP patients (*n* = 90) from another cohort and predicted the COCA subtypes among these samples (Supplementary Tables S10, S11). All normal samples from healthy individuals (*n* = 10) were accurately identified.

Analyses of the clinical characteristics of each cluster also indicated an increase in diabetes and steatorrhea morbidity from COCA1 to COCA3 (Supplementary Table S12). Moreover, follow-up of patients who did not have steatorrhea or diabetes at the time of sample collection revealed a significant increase in the incidence of steatorrhea and diabetes in patients with the COCA3 or COCA2 subtype compared with those with the COCA1 subtype (Fig. 4g, *P* < 0.05). To further validate the diagnostic utility of the classification system for CP, we downloaded miRNA data from public databases (GSE263996, GSE218599) representing 15 cases of osteoarthritis and 3 cases of rheumatoid arthritis. After batch effect removal, these datasets were combined with the miRNA data from our local exploratory cohort of 89 CP patients. The COCA classifier was then applied to predict CP status on the basis of the expression levels of the 12 predefined miRNAs.

Among the 107 subjects (89 with CP and 18 with other diseases), 78 out of 89 predicted CP cases were correctly identified by the COCA algorithm (sensitivity: 87.6%, specificity: 77.8%; Supplementary Table S13). These results preliminarily demonstrate that our algorithm not only exhibits predictive efficacy for CP-associated complications (e.g., steatorrhea and diabetes) but also has potential diagnostic value for distinguishing CP from non-CP conditions.

### Identification of the pivotal role of interaction between exosome-derived miR-24-3p and S100A8 in the CP diagnostic classifier and the use of neutrophils as resources for exosomal S100A8 in CP

**Fig. 5:**
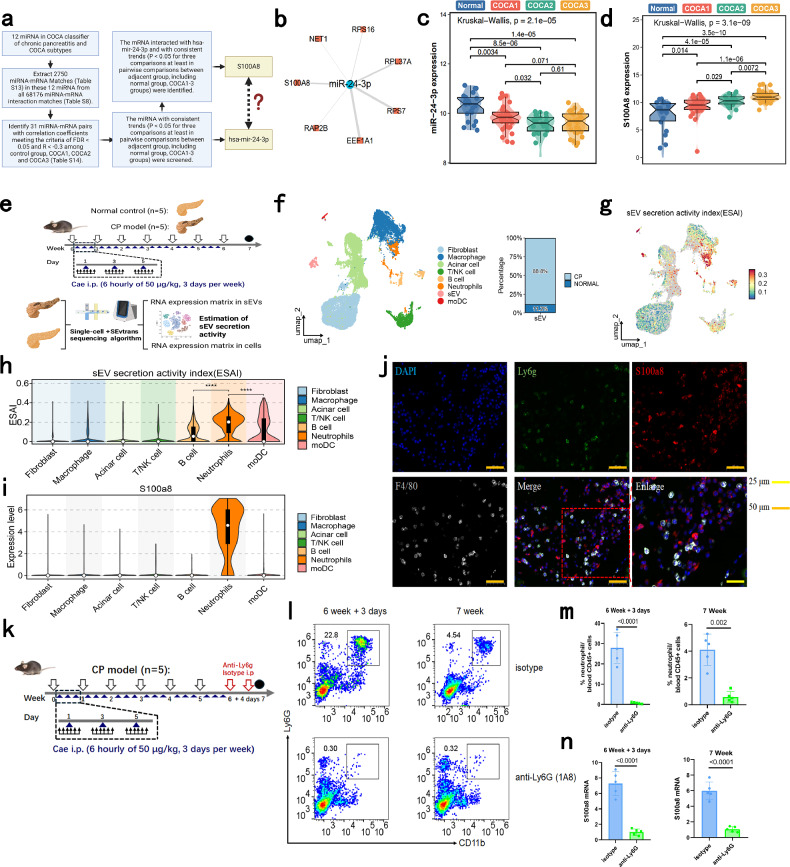
Identification of the exosome-derived miR-24-3p and S100A8 interaction as a key factor in the CP diagnostic classifier and of neutrophils as resources for exosomal S100A8 in CP. **a** Overview of the filtering strategy. **b** The screened miRNA (mir-24-3p)–mRNA interaction network. **c** The expression of miR-24-3p in each COCA subtype and normal samples. **d** S100A8 expression in each COCA subtype and normal samples. **e** Schematic diagram of the CP mouse model construction, single-cell data processing, and estimation of exosome secretion activity using the sEVtrans algorithm. **f** Left: UMAP plot of all cell types and identified exosomes (sEVs) in the single-cell sequencing data. Right: proportion of exosomes from the CP group and NC group. **g** UMAP plot showing the exosome secretion activity index (ESAI) value distributions among different cells. **h** Violin plot showing the ESAI distributions in different cell types. ****P < 0.0001. **i** Violin plot showing the expression of S100A8 in different cell types. **j** Representative images of Ly6G, F4/80 and S100A8 immunofluorescence in the mouse CP model. **k** Schematic diagram of the CP mouse model constructed with intraperitoneal injection of a neutrophil depletion antibody (anti-Ly6g) compared with that generated by isotype injection. **l** Representative flow cytometry image after establishment of the CP model compared with that after mock establishment via isotype injection. **m** Neutrophil counts in CD45^+^ blood cells in CP mice after model establishment compared with mock establishment. **n** mRNA expression of exosomal S100A8 in the CP mice after model establishment compared with mock establishment.

Given that miRNAs and their target genes have been reported as key mechanisms of exosome-induced disease progression^28^, we sought to further elucidate the mechanism of the 12 miRNAs in CP progression. We extracted 2750 mRNAs identified as potential interaction partners of the 12 miRNAs in our diagnostic panel (Supplementary Table S9); these 2750 genes are listed in Supplementary Table S14. We then filtered the predicted miRNA‒mRNA pairs in this 12-miRNA COCA diagnostic classifier via miRNA‒mRNA interaction estimation and correlation analysis, as illustrated in Fig. 5a. Specifically, miR-24-3p, miR-20a-5p, and their putative mRNA networks were identified as the pivotal factors (Fig. 5b; Supplementary Fig. S5a left and Table S15). Because the expression of miR-24-3p showed a more consistent downward trend from the normal samples to the COCA1–3 samples than miR-20a-5p (Fig. 5c; Supplementary Fig. S5a right), miR-24-3p was chosen for subsequent experiments. Similarly, among the putative target mRNAs of miR-24-3p, only S100A8 showed a consistent upward trend among the normal samples and COCA1–3 samples (Fig. 5d; Supplementary Fig. S5b). Therefore, we next focused on whether the interaction between miR-24-3p and S100A8 could affect the development of CP.

Our first question was which type of cell primarily releases exosomes in CP. As illustrated above, we preliminarily confirmed that specific proteins and RNAs released by damaged pancreatic parenchymal cells in CP can be identified in circulating exosomes. Thus, we performed single-cell sequencing on pancreatic tissue from CP patients and normal controls. The sEVtrans algorithm was also used to estimate the exosome secretion activity index (ESAI) among all cell types (Fig. 5e). We further extracted exosomes from mouse pancreas tissues and measured the expression levels of miR-24-3p and S100A8 mRNA in these samples. First, the sequences of mir-24-3p were compared between mice and humans to evaluate their homology; this comparison indicated that the sequence of mmu-mir-24-3p is the same as that of hsa-mir-24-3p (Supplementary Fig. S5c). The expression of mmu-mir-24-3p in exosomes was significantly lower in the CP group than in the normal (NC) group, and the expression of S100A8 in CP exosomes was significantly greater in the CP group (Supplementary Fig. S5d).

Then, cell annotation was performed on the basis of the single-cell sequencing data after separation of the exosome data using sEVtrans quality control (Supplementary Fig. S5e, f) and cell clustering (Supplementary Fig. S5g, h). Macrophages, T cells, NK cells, acinar cells, fibroblasts, neutrophils, monocyte-like dendritic cells (moDCs), naïve B cells, plasma, and red cells were identified (Supplementary Fig. S5i). We then combined the exosome data from sEVtrans with the single-cell sequencing data and determined that 88.8% of the exosomes were derived from the CP group (Fig. 5f). Next, the ESAI values of the cell types were determined using sEVtrans. Neutrophils were identified as the core exosome-secreting cell type (Fig. 5g, h). Moreover, S100A8 mRNA expression was detected only in neutrophils (Fig. 5i), and S100A8 protein expression was detected in Ly6g^+^ neutrophils but not in F4/80^+^ macrophages (Fig. 5j).

To verify that neutrophils are the source of exosomal S100A8 mRNA, we performed neutrophil depletion in a mouse model of CP (Fig. 5k; Supplementary Fig. S5j). Intraperitoneal injection of anti-Ly6g antibody at 6 and 6 weeks + 4 days successfully depleted the neutrophils (Fig. 5l, m). We then extracted the exosomes from the plasma and detected the expression levels of S100A8 and mmu-mir-24-3p. The expression of the S100A8 mRNA significantly decreased after neutrophil depletion in CP model mice (Fig. 5n), and no significant change in the expression of mmu-mir-24-3p was observed (Supplementary Fig. S5k). Moreover, the pathological score was significantly decreased after neutrophil depletion in CP model mice (Supplementary Fig. S5l). Together, these results demonstrate that while neutrophils are the source of S100A8 mRNA in the plasma exosomes of CP, exosomal miR-24-3p is not generated by neutrophils.

### Exosome-derived miR-24-3p loss leads to S100A8 upregulation in neutrophils, enhancing the fibrotic phenotype in pancreatic stellate cells by the S100A8-TLR4/RAGE-ROS axis

**Fig. 6:**
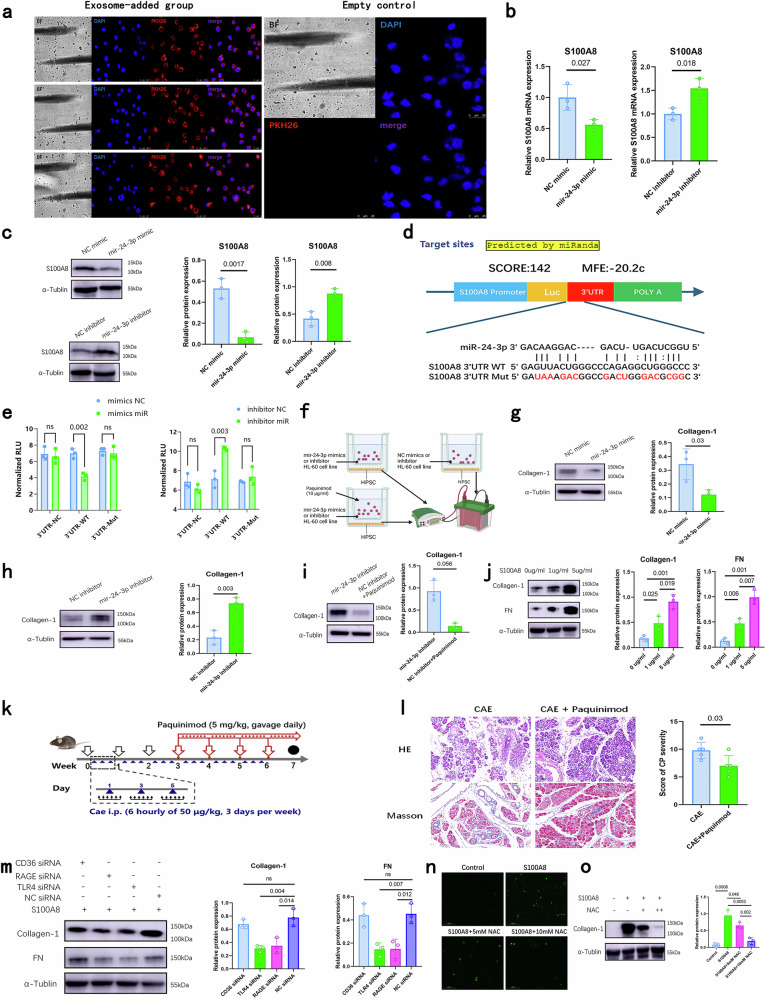
Exosome-derived mir-24-3p loss leads to S100A8 upregulation in neutrophils, enhancing fibrotic phenotype in pancreatic stellate cells via the S100A8-TLR4/RAGE-ROS axis. **a** Exosomes loaded with PKH26 dye can be taken up by HL-60 neutrophils, including the exosome-supplemented group and the empty control group. **b** S100A8 mRNA expression in HL-60 cells after transfection with miR-24-3p or S100A8. *n* = 3 per group. **c** Protein expression of S100A8 in HL-60 cells after transfection with miR-24-3p mimic or inhibitor. *n* = 3 per group. **d** Design of the luciferase reporter vector with sequencing alignment of miR-24-3p with the 3’-UTR of the S100A8 gene. **e** Luciferase activity of the human S100A8 3’-UTR in HEK293T cells. Cells were transfected with empty plasmid (NC), WT, or mutant 3’-UTR (Mut) plasmids together with miR-24-3p mimics or inhibitors. *n* = 3 per group. **f** Schematic diagram of the neutrophil and pancreatic stellate cell co-culture system. Relative protein levels of collagen-1 in pancreatic stellar cells after co-culture with mir-24-3p mimic (**g**), inhibitor (**h**), or paquinimod (**i**). *n* = 3 per group. **j** The protein levels of collagen-1 and FN in pancreatic stellate cells after exogenous supplementation with S100A8. *n* = 3 per group. **k** Schematic diagram of paquinimod administration in the CP mouse model. **l** Representative HE and Masson sections from CP model mice with or without paquinimod injection (left) and the pathological scores of the two groups. *n* = 5 per group. **m** The protein levels of collagen-1 and FN in pancreatic stellate cells after exogenous supplementation with S100A8 (1 µg/mL) and transfection with CD36, TLR4, RAGE or NC siRNA. *n* = 3 per group. **n** Representative fluorescence images of ROS in pancreatic stellate cells from the control group, S100A8 (1 µg/mL) group, S100A8 (1 µg/mL) + 5 mM NAC group, and S100A8 (1 µg/mL) + 10 mM NAC group. **o** The protein levels of collagen-1 and FN in pancreatic stellate cells after exogenous supplementation with S100A8 (1 µg/mL) or NAC (5 mM or 10 mM). *n* = 3 per group.

Having identified mir-24-3p and S100A8 as a pivotal interaction pair in plasma exosomes from CP patients, and having demonstrated that exosomal S100A8 mRNA is derived mainly from neutrophils, we hypothesized that neutrophils are the effector cells of the mir-24-3p and S100A8 interaction. Therefore, we next tested whether neutrophils could take up exosomes from the plasma of CP patients. We successfully induced a neutrophil-like phenotype in HL-60 cells in vitro (Supplementary Fig. S6a). Subsequent detection of exosome uptake by these cells revealed that exosomes derived from the plasma of CP patients can be taken up by these neutrophil-like HL-60 cells (Fig. 6a; Supplementary Fig. S6b). Moreover, the upregulation of miR-24-3p in these cells inhibited the expression of S100A8 at the mRNA and protein levels, and the opposite effect was observed upon miR-24-3p knockdown (Fig. 6b, c; Supplementary Fig. S6c). Furthermore, we successfully separated peripheral neutrophils from CP patients and healthy donors (Supplementary Fig. S6d). Exosome uptake detection also revealed that exosomes derived from the plasma of CP patients can be taken up by separated neutrophils (Supplementary Fig. S6e). Moreover, we observed that the S100A8 mRNA and protein levels in neutrophils from CP patients were significantly greater than those in neutrophils from healthy controls (Supplementary Fig. S6f, g).

Next, we predicted the 3’-untranslated region (UTR) binding site between miR-24-3p and S100A8 via miRanda software and constructed luciferase reporter vectors bearing mutated and wild-type (WT) binding sites within the 3’-UTR of S100A8 (Fig. 6d). To further test whether miR-24-3p can directly target S100A8, we transfected these luciferase reporter vectors into HEK293T cells. Luciferase reporter assays revealed that the luciferase reporter activity of the WT 3’-UTR was strongly inhibited by the miR-24-3p mimics and increased by the miR-24-3p inhibitor (Fig. 6e).

Subsequently, to assess the profibrotic effect of neutrophils on human pancreatic stellate cells (HPSCs), a co-culture system was designed (Fig. 6f). Upregulation of miR-24-3p decreased the expression of collagen-1 and vice versa (Fig. 6g, h). The addition of the S100A8 inhibitor paquinimod inhibited the increase in collagen-1 caused by the inhibition of miR-24-3p in neutrophils (Fig. 6i). Moreover, the addition of S100A8 to pancreatic stellate cells increased the expression of fibrosis markers in a dose-dependent manner (Fig. 6j). In the co-culture system, the protein level of collagen-1 in HPSCs co-cultured with neutrophils from CP patients was significantly greater than that in HPSCs from healthy controls (Supplementary Fig. S6h). The addition of paquinimod inhibited the increase in the protein level of collagen-1 in HPSCs co-cultured with neutrophils from CP patients (Supplementary Fig. S6i). Next, to test whether similar effects occur in vivo, we explored the anti-fibrotic effect of S100A8 inhibition therapy in a CP mouse model (Fig. 6k). Paquinimod administration partly alleviated the severity of CP (Fig. 6l) but had no significant effect on the M2 polarization of macrophages in the pancreatic tissues of CP model mice (Supplementary Fig. S6j). Moreover, paquinimod administration decreased immune cells infiltration and also decreased the expression of S100A8 in pancreatic neutrophils (Supplementary Fig. S6k, l).

Next, we sought to identify the exact functional receptor of S100A8 in pancreatic stellate cells. A previous study identified three potential receptors for S100A8: TLR4, RAGE, and CD36^30^. We successfully knocked down these three genes (Supplementary Fig. S6m–o). In HPSCs, the expression of fibrosis-related proteins, including Collagen-1 and fibronectin (FN), was inhibited in the presence of S100A8 following the knockdown of RAGE or TLR4, but not after CD36 knockdown (Fig. 6m). Further, because reactive oxygen species (ROS) have been reported to be involved in common biological processes after the activation of RAGE or TLR4^31–33^, we evaluated whether ROS blockade could impact S100A8-induced fibrosis in pancreatic stellar cells. Notably, treatment with NAC (a ROS inhibitor) inhibited ROS and fibrosis in S100A8-induced pancreatic stellate cells in a dose-dependent manner (Fig. 6n, o).

### S100A8 exacerbates β-cell apoptosis and islet inflammation through the TLR4-mediated inflammatory response of macrophages

**Fig. 7:**
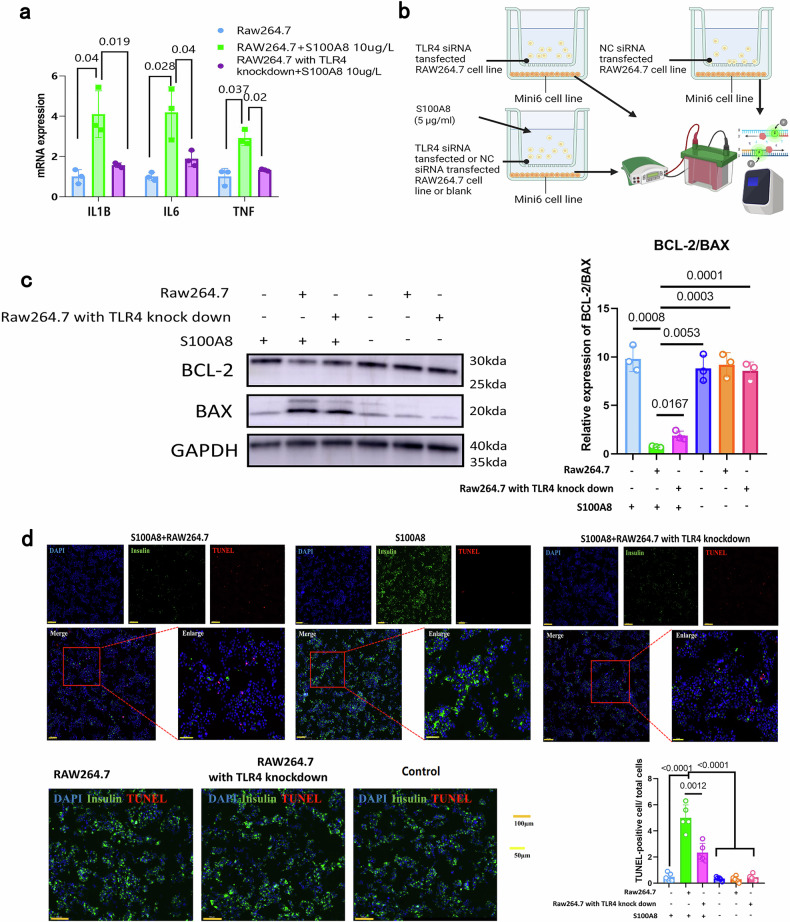
S100A8 exacerbates β-cell apoptosis and islet inflammation by regulating the TLR4-mediated inflammatory response of macrophages. **a** mRNA expression of IL-1B, IL6, and TNF in RAW264.7 cells after exogenous supplementation with S100A8 (1 µg/mL) and transfection with or without TLR4 siRNA. *n* = 3 per group. **b** Schematic diagram of the macrophage and pancreatic stellate cell co-culture system. **c** Relative protein levels of BCL-2 and Bax in MIN6 cells after culture with RAW264.7 cells, TLR4-knockdown RAW264.7 cells, and/or exogenous supplementation with S100A8 (1 µg/mL). *n* = 3 per group. **d** Representative immunofluorescence image and TUNEL-positive cell counts of MIN6 cells after co-culture with RAW264.7 cells, TLR4-knockdown RAW264.7 cells, and/or exogenous supplementation with S100A8 (1 µg/mL). *n* = 5 per group.

**Fig. 8:**
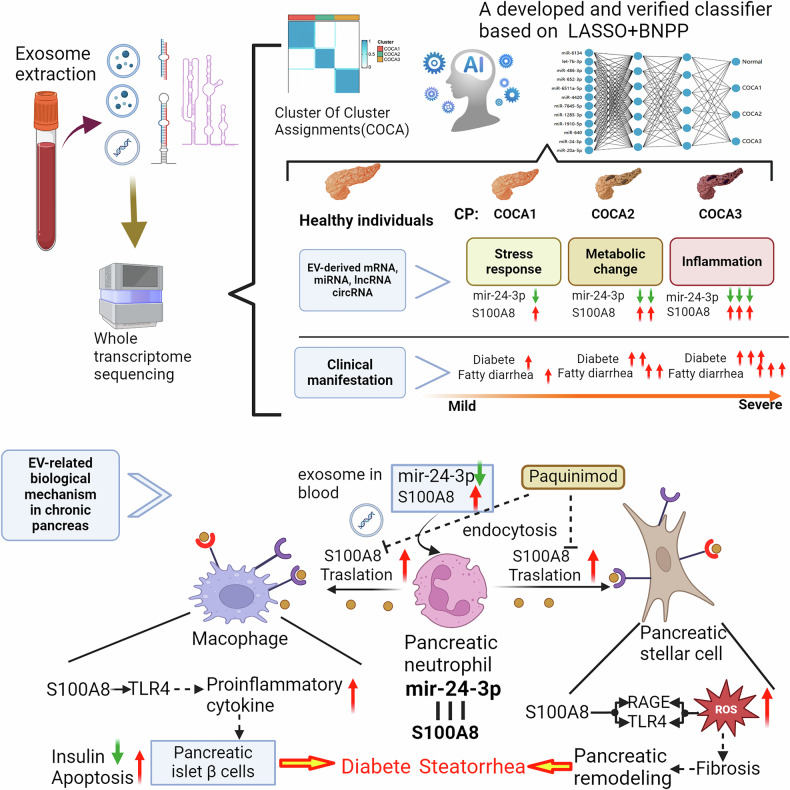
Diagram of the diagnostic workflow and mechanism of CP exacerbation proposed in this study. The diagnostic workflow and mechanism for CP exacerbation proposed in this study include the development and validation of apersonalized AI-based risk stratification model for CP (above), as well as the elucidation of the mechanism by which exosome derived miRNAs contribute to the exacerbation of diabetes and steatorrhea in CP (down).

Finally, we explored the potential mechanism of S100A8-mediated pancreatic endocrine disorders. One previous study reported that signaling between pancreatic β-cells and macrophages involving S100A8 and TLR4 exacerbates β-cell apoptosis and islet inflammation in type 2 diabetes^34^. We hypothesized that a similar mechanism could link the abnormally increased levels of S100A8 with pancreatic β-cell apoptosis in CP. To test this idea, we knocked down TLR4 in RAW264.7 macrophages (Supplementary Fig. S5j) and co-cultured them with or without S100A8. In WT macrophages, the mRNA levels of inflammatory cytokines, including IL-1B, IL-6, and TNF, increased after S100A8 stimulation, and this increase was reversed after TLR4 knockdown (Fig. 7a). These results suggest that S100A8 promotes the inflammatory response of macrophages through TLR4. Next, we designed a co-culture system to verify the impact of macrophages on the apoptosis of pancreatic β-cells (Fig. 7b). Western blot and immunofluorescence revealed that S100A8 promoted the apoptosis of pancreatic β-cells in the presence of macrophages. The knockdown of TLR4 in macrophages could partly reverse this apoptotic phenotype of pancreatic β-cells, and S100A8 alone could not induce the apoptosis of pancreatic β-cells in the absence of macrophages (Fig. 7c, d). These results suggest that S100A8 can promote the inflammatory phenotype of macrophages and that S100A8 can promote pancreatic β-cell apoptosis through the activation of TLR4 in macrophages. Diagrams illustrating the proposed mechanism and the diagnostic scheme presented in this study are shown in Fig. 8.

## Discussion

CP has a reported global incidence ranging from 42 to 73 per 100,000, with a rising prevalence observed over time^35^. Most individuals with CP are managed on an outpatient basis. However, records from the United States, the United Kingdom, the Netherlands, and Finland indicate a concerning increase in annual hospital admissions, with a reported 30% increase over a six-year period, underscoring the major socioeconomic impact of this disease. Mortality rates associated with CP are estimated to be between 12.8% and 19.8% on the basis of mean observation periods of 6.3–9.8 years^35–38^. Additionally, approximately 33% of patients with CP find themselves unable to continue working^39^. Given these considerations, researchers have put considerable effort into the development of a stable classification system to predict the severity of CP. The 1963 Marseilles classification focused on morphology and etiology^8^ and was later refined in 1985 as the Marseilles–Rome classification with functional correlations^9,10^. The Cambridge classification introduced imaging-based grading using ERP/CT findings but faced limitations in reflecting early-stage functional/histological changes^11,12^. Recent systems integrate imaging, symptoms, interventions, and functional loss to stratify severity^14–18^. However, these classifications do not rely on molecular biomarkers, reducing their clinical accuracy.

Over the past two decades, molecular studies have revealed CP heterogeneity: genetic mutations disrupt trypsin regulation or calcium signaling. Emerging molecular subtyping categorizes CP into genetic, alcohol-related, and idiopathic forms^1^. However, these etiological frameworks lack multi-omics biomarker integration, limiting precision in stratifying the risk of complications, including steatorrhea and diabetes.

Currently, there are no widely used models for the assessment of CP severity or the risk of two severe complications caused by exocrine and endocrine disorders, steatorrhea and diabetes. Using liquid biopsy, single-cell sequencing data from the pancreatic lesions of CP patients, and whole-transcriptome sequencing of peripheral blood exosomes, we show for the first time that the mRNA alterations in the pancreatic cells of CP patients are recapitulated in circulating exosomes. This finding indicates that circulating exosomes could serve as a reflection of the molecular changes occurring within the pancreas, suggesting a non-invasive approach for monitoring CP progression and complication risk. These findings are consistent with some recent results of studies involving different kinds of cancers, including prostate cancer, breast cancer, and lung cancer^25,40,41^; specifically, the components of exosomes in the bloodstream were associated with various malignant phenotypes from local lesions and could serve as novel biomarkers. Our current findings extend this idea from cancer to noncancer chronic diseases.

Another highlight of our study is the successful development and use of a COCA-based subtyping system based on whole-transcriptome sequencing of circulating exosomes. Given the multidimensionality of these data, including mRNA, miRNA, circRNA, and lncRNA data, a viable multidimensional clustering algorithm is needed. Most previous multiplatform-based molecular cancer subtyping studies, including those based on lung cancer^42^, esophageal cancer^43^, urothelial carcinoma^44^ and pancancer datasets^27^, have used the COCA algorithm. However, to our knowledge, this algorithm has not been previously applied to a noncancer chronic disease. In our study, the application of COCA yielded 3 clusters (COCA1–3), which were identified as subtypes of CP. Each cluster exhibited the activation of distinct biological processes, as identified via WGCNA, namely, the stress response for COCA1, metabolic changes for COCA2, and inflammation for COCA3. The mRNA levels of the pancreas-derived exocrine and endocrine signature genes also increased significantly from COCA1 to COCA3. Additionally, consistent with these findings, the incidence of steatorrhea and diabetes tended to increase from COCA1 to COCA3. These results preliminarily confirmed the viability of our current classification.

The miRNAs in exosomes are relatively stable, highlighting their potential as robust biomarkers for disease diagnosis^28^. To increase the clinical translation potential of our current classification system, we constructed a 12-miRNA classifier based on circulating exosomes via LASSO + BPNN. A previous study indicated that BPNNs can efficiently extract key features included in exceedingly complicated nonlinear hidden neural layers and can handle complex and nonlinear problems more easily than conventional logistic regression models^45^. In our current study, the COCA1–3 subtypes and healthy samples were all successfully distinguished, with more than 80% accuracy rates in both the reanalysis of the sequencing data and analysis of qPCR data for these 12 miRNAs among the enrolled samples. Moreover, the application of the model to qPCR data from the validation group revealed a robust ability to distinguish CP patients from healthy individuals and a tendency of increasing steatorrhea and diabetes from COCA1 to COCA3. This classification system enables personalized risk assessment for CP complications. For example, patients identified as high risk may benefit from early interventions, such as extracorporeal shock wave lithotripsy and/or endoscopic retrograde cholangiopancreatography, for more active protection of pancreatic function. Conversely, low-risk patients could avoid unnecessary treatments, optimizing resource allocation. Future studies should explore whether tailoring therapies on the basis of exosome-derived RNA signatures improves clinical outcomes. Additionally, this classifier has been uploaded to the Gitee database so that it can be accessed by any clinician or researcher.

Our current study also revealed the pivotal role of decreased exosome-derived miR-24-3p and increased neutrophil-derived S100A8 in pancreatic fibrosis and the apoptosis of pancreatic β-cells for the first time. Several previous studies have reported that miR-24-3p inhibits fibrosis and inflammation^46,47^. Here, we reported the function of decreased exosome-derived miR-24-3p in pancreatic fibrosis and that it prevents the apoptosis of pancreatic β-cells occurs through its direct targeting of S100A8 in neutrophils. S100A8, an S100 protein, has been reported to be an important contributor to fibrosis in multiple diseases, including diabetic nephropathy^48^, hepatic fibrosis^49,50^ and pulmonary fibrosis^51^. Additionally, one previous study revealed that S100A8 could promote apoptosis in pancreatic β-cells^34^. Extending these previous findings, we showed that S100A8 promoted pancreatic fibrosis through the activation of TLR4/RAGE and ROS in pancreatic stellate cells and that the proapoptotic effect of S100A8 on pancreatic β-cells in CP occurred through the promotion of pancreatic macrophage inflammation. These results indicate that S100A8 inhibition could be a potential therapeutic strategy for both exocrine and endocrine disorders associated with CP, including steatorrhea and diabetes.

However, this study also has several limitations. First, all the examined samples were obtained from specimen repositories at different hospitals. Further prospective studies of our COCA classifier are needed to verify its ability to predict steatorrhea and diabetes in CP patients. Second, the etiology of CP in our enrolled patients was not clearly defined, because the TIGAR-O Risk/Etiology Checklist^52^ is not commonly used in Chinese hospitals. Third, the impact of S100A8 on the apoptosis of pancreatic β-cells has not been well studied in vivo. Additionally, while paquinimod reduced CP severity in mice, its clinical translation requires caution. Its off-target effects and limited tissue specificity may hinder its applicability. Further in-depth studies are needed to address these limitations.

In conclusion, we propose a new 3-stage system for distinguishing CP patients from healthy individuals and developed an online tool for predicting the risk of steatorrhea and diabetes in CP patients via exosome-related liquid biopsy data. Additionally, our study revealed the pivotal role of exosome-derived miR-24-3p in directly targeting S100A8 in neutrophils, showing that neutrophil-derived S100A8 promoted pancreatic fibrosis through the activation of TLR4/RAGE and ROS in pancreatic stellate cells and that the proapoptotic effect of neutrophil-derived S100A8 in pancreatic β-cells in CP was induced through the promotion of pancreatic macrophage inflammation.

## Materials and methods

### Patients and samples

Between October 2017 and October 2019, plasma samples from 140 chronic pancreatitis patients and 27 healthy donors were collected and frozen in Changhai Hospital in Shanghai, China. These blood samples were obtained from chronic pancreatitis patients hospitalized for scheduled pancreatic duct stone removal using ERCP. Based on the previously reported preoperative indications and contraindications of ERCP for pancreatic stones, acute pancreatitis flare is contraindicated to surgery and patients with an acute episode of chronic pancreatitis were excluded from hospitalization^53^. In addition, between October 2018 and October 2020, plasma samples from 39 chronic pancreatitis patients and 5 healthy donors were collected and frozen in the First Affiliated Hospital of Zhengzhou University in Zhengzhou, China. The blood samples from these chronic pancreatitis patients were collected when these patients were routinely admitted for examination, and patients with an acute episode of chronic pancreatitis were excluded. The diagnosis and the clinical stage of chronic pancreatitis were followed by the Guidelines for the diagnosis and treatment of chronic pancreatitis in China (2018 edition)^54^. All of our enrolled patients in this study are more than 20 years old and without a family history of pancreatic disease, so hereditary factors are not considered. Blood samples were taken in the morning using EDTA‐containing tubes and centrifuged at 2890 × *g* at 4 °C for 10 min, then the isolated plasma was stored at −80 °C. All diagnoses and treatment methods for chronic pancreatitis were carried out in accordance with existing guidelines^55–57^. Information, including smoking and alcohol consumption, demographic data (age, sex, etc.), and diabetes mellitus, was documented in detail in Tables 1 and 3. All patients provided written informed consent prior to participation. Ethical approval for this research was granted by the Ethics Committee of Changhai Hospital (CHEC2017‐232) and the Ethics Committee of the First Affiliated Hospital of Zhengzhou University (2018-KY-0324). All enrolled patients were followed up for about 4 years to investigate whether new-onset steatorrhea or diabetes mellitus could be diagnosed. This study adheres to the ethical guidelines of the Helsinki Declaration.

### RNA sequencing of plasma exosomes

Total exosomal RNA from each sample was extracted by TRIzol reagent (Invitrogen, USA) according to the manufacturer’s instructions. The quality and purity of the extracted RNAs were measured using an Agilent 2100 Bioanalyzer (Agilent Technologies, USA). The sequencing library was performed using NEXTflex^®^Small RNA-Seq Kit v3 (NOVA-5132-05, Bio Scientific Corporation, USA) or Ovation Human FFPE RNA-Seq Library System (0340-32, NUGEN, USA), and a sample input of 20 ng of each total RNA was used. Finally, we profiled the expression of the sequencing library using HiSeq 2500 (Illumina, Inc., San Diego, CA, USA). Cutadapt software was utilized to remove low-quality reads and acquire high-quality reads^58^. The comparison software Tophat (http://ccb.jhu.edu/software/tophat/index.shtml) was used to map clean reads to a reference genome annotated with a gene location, and Bowtie2 (http://ccb.jhu.edu/software/tophat/index.shtml) was applied to build the index of the reference genome. CircRNAs and the host gene of circRNAs were identified by find_circ^59^ and CIRCexplorer^60^. We used StringTie software to assemble the transcripts and utilized Perl scripts to screen known lncRNAs. Quantitative analysis of lncRNAs and mRNAs was performed using the Ballgown R package.

### Differential expression

Differential exosomal mRNA, lncRNA, miRNA, and circRNA expression analysis of the two groups was conducted by limma package in R. Fold change (FC) was regarded as an indicator of differential expression between the CP group and the control group. *t*-tests were utilized to evaluate the statistical significance of differences. *P*-values < 0.05 were considered to demonstrate differential expression.

### Prediction of target genes of LncRNA

LncRNA target gene prediction Target gene prediction of lncRNAs was carried out in two ways: the *cis*-acting target gene prediction, and *trans*-acting target gene prediction. Based on the theory of *cis*-acting regulatory element, the protein-coding genes located within 10 kb from lncRNA were selected as potential *cis*-acting target^61^. While for *trans*-acting target prediction, the Pearson correlation coefficients between the coding genes and lncRNAs were calculated and analyzed for the identification of *trans*-acting regulatory elements (Coefficients > 0.5).

### Identification of the interaction pairs about miRNA–mRNA

68,176 potential miRNA–mRNA interaction networks with Experimental Evidence were downloaded from the miRTarBase (Supplementary Table S9). Then, the 68,176 predicted miRNA‒mRNA regulatory relationships were integrated with the DEM data to acquire the miRNA-DEM regulatory relationship. Then, the ceRNA networks were developed using Cytoscape (version 3.6.1; http://www.cytoscape.org/).

### Pathway enrichment analysis

The mRNAs targeted by the differentially expressed miRNAs, mRNA of *cis*-acting regulatory and *trans*-acting regulatory lncRNAs, mRNA transcribed by host genes of circRNA were subjected to GO and Kyoto Encyclopedia of Genes and Genomes pathway enrichment analysis using the clusterProfiler package. This analysis was performed to identify enriched biological processes, molecular functions, cellular components, and pathways associated with these mRNAs. A Benjamini–Hochberg adjusted *P*-value (FDR) of less than 0.05 was considered significant for the enrichment results.

### scRNA-seq data processing, cluster annotation, and data integration for human pancreas

The single-cell data of 13 chronic pancreatic tissues were downloaded from http://singlecell.charite.de/cellbrowser/pancreas/?ds=Chronic_Pancreatitis^26^ using Seurat package (v4.4.0)^62^. Cells expressing less than 500 genes as well as more than 5000 genes, cells with a proportion of mitochondria higher than 10%, cells with a total number of mRNA molecules detected less than 500, cells with erythrocyte content higher than 3% and genes expressed in less than three cells, were excluded from the downstream analysis. The “SoupX’ method from the SoupX package (v1.6.2)^63^ and the “doubletFinder_v3” method from the DoubletFinder package (v2.0.3)^64^ were applied for additional cell filtering. Filtered data were then log-normalized and scaled, with cell–cell variation due to UMI counts and percent mitochondrial reads regressed out. A total of 2000 features for anchoring (the “FindVariableFeatures” function) and 40 dimensions for alignment (“Integrate Data”) were used. Cell clustering was performed by “FindClusters” function at a resolution of 0.1. Dimensionality reduction was performed with “Run UMAP” function and visualized by UMAP. For subgroup cell clustering, cells of different types were extracted separately and clustered by their respective first 30 principal components using different resolutions based on visual inspection.

### Integrated COCA

Subtypes derived from each of the four platforms, mRNA, miRNA, lncRNA, and circRNA, were calculated as described above. Subtype calls for each of the four platforms were coded into a series of indicator variables for each subtype, following the definitions in Monti et al.^65^. The binary matrix was used as the input for consensus clustering in the ConsensusClusterPlus R package^66^. Specifically, 1000 iterations of 80% resampling from 2 to 10 clusters were done using PAM method based on a Pearson correlation distance metric. The desired number of clusters was determined based on the proportion increase Δ(*K*) in the area under the CDF, where

$$\varDelta (K)=\left\{\begin{array}{cc}A(K) & K=2\\ \frac{A(K+1)-A(K)}{A(K)} & K > 2\end{array}\right.$$

and A(*K*) is the area under curve of *K* clusters (*K* = 2, 3, …, 10). We chose the number of clusters where further increase in the number did not remarkably increase the A(*K*) and the main groups were stable on the consensus clustering map.

### Identification of exocrine and endocrine signature genes and subgrouping

We applied the “FindAllMarkers” function in Seurat to identify specific genes for each cell subset. For the selection of marker genes specific to each cell cluster/subset, we calculated the log_2_ FC between two groups (a cell cluster/subset vs other cells) using the “findallmarkers’ function with the Wilcoxon rank-sum test (default parameters). Gene markers of cell types and each cell subgroup were chosen according to a previous study, and the cell subgroup was annotated according to these gene markers. Then, the exocrine disorder signature genes are identified as the top 40 genes (calculated by log_2_FC) in Acinar REG^+^ cells and activated stellate cells (Supplementary Table S6), and the endocrine signature genes are also identified, consisting of the top 40 marker genes (calculated by log_2_FC) of alpha cells and beta cells (Supplementary Table S7). Median expression levels of coexpressed exocrine (*X* axis) signature genes and endocrine (*Y* axis) signature genes were used to visualize the distribution of each sample in coordinate graphs.

### WGCNA

Clusters of highly correlated genes associated with each COCA cluster were identified via a WGCNA approach^67^, which was conducted using R (v 3.6.3). Soft-thresholding power (*β*) was established with the pickSoftThreshold R function to better detect strong correlations between gene modules. Hierarchical clustering analyses were then conducted to detect modules. Interaction strength was assessed with the Heatmap tools package, with gene significance (GS) and module membership (MM) being calculated to assess relationships.

### Construction of a classifier for COCA classification and diagnosis of CP

To facilitate the clinical translation of our research findings, we employed a resilient backpropagation-based neural network algorithm for modeling miRNA expression profiles, aiming to accurately predict chronic pancreatitis and its subtypes, thereby enhancing the clinical applicability of our study. The workflow is as follows:

1. Feature Selection: For each subtype and healthy samples, we first retained miRNAs with a *P*-value < 0.01 from univariate logistic regression. Subsequently, we employed the Bootstrapping method to randomly sample 70% of the data from all samples, performing logistic regression over 1000 iterations. We retained genes that remained significant (*P* < 0.05) in over 95% of the resampling processes. Following this, we utilized the Lasso algorithm for further dimensionality reduction and model simplification, keeping those features with non-zero Lasso coefficients as input variables for modeling.
2. Hyperparameter Optimization: We divided all samples into training and test sets in an 80:20 ratio. The neural network model was constructed using the “neuralnet” package in R software. The parameters to be optimized included the learning rate, loss function, activation function, number of hidden layers, and the number of nodes in each layer. Hyperparameter optimization was conducted using a grid search approach, with the final model's parameters chosen based on the combination that yielded the highest accuracy on the test set.
3. Model Validation: Model performance was assessed using confusion matrices on the training set, the test set, and the entire dataset.

### Identify the key miRNA–mRNA interaction pair in the 12 miRNA COCA classifier

The procedure flow chart is shown in Fig. 5a. Firstly, the 12-miRNA-related mRNA with potential binding ability is screened from the previously acquired Supplementary Table S9. We identified 2750 miRNA–mRNA in this procedure (Supplementary Table S14). Then, based on our results of whole transcriptome sequencing, 31 miRNA–mRNA pairs with correlation coefficients (*R* value, Pearson correlation coefficient) meeting the criteria of FDR < 0.05 and R < –0.3 among the control group, *COCA1*, *COCA2*, and *COCA3* were identified (Supplementary Table S15). Next, the miRNAs with consistent trends (*P* < 0.05 for three comparisons at least in pairwise comparisons between adjacent groups, including the normal group, COCA1–3 groups) were screened. Finally, mRNA interacted with the screened miRNA and with consistent trends (*P* < 0.05 for three comparisons at least in pairwise comparisons between adjacent group, including the normal group, COCA1–3 groups) were identified (S100A8 and miR-24-3p).

### Cell culture

HL-60 cell line (RCB3683, RIKEN BioResource Center, Ibaraki, Japan) was cultured in an RPMI 1640 medium containing 10% (v/v) FBS and 1% penicillin/streptomycin (PS). To differentiate HL-60 cells into neutrophil-like cells, the cells were cultured with 1.25% DMSO for 3 days. HPSCs were generously provided by Prof. Logsdon CD from the Anderson Cancer Center’s Department of Cancer Biology, Houston, Texas, USA^68^. Raw 264.7 cells were sourced from the American Type Culture Collection (ATCC, USA). HEK-293 cells were also sourced from the American Type Culture Collection. Both HPSCs, HEK-293 T cells, and Raw 264.7 cells were cultured in DMEM supplemented with 10% FBS and 1% PS. Islet β-cell MIN6 cells (ATCC, USA) were cultivated in DMEM (25 mmol/L glucose) containing 1% PS, 10% FBS, and 1% β-mercaptoethanol. All cells are cultured in a 5% CO_2_ environment at 37 °C, with medium changes every 2 days.

### Cell transfection

We collected cells at the exponential growth phase for further assays. Specifically, HL-60 cells were treated with 1.25% DMSO for 3 days. The hsa-miR-24-3p mimics, hsa-miR-24-3p inhibitor, NC mimic, and NC inhibitor (Hanbio, Shanghai, China) were transfected into ADSCs using Lipofectamine 2000 (Thermo Fisher Scientific, USA) following the manufacturer's instructions. For HPSCs, small interfering RNA (siRNA)-to knock down CD36 (si-36), RAGE siRNA (si-RAGE), TLR4 siRNA (si-TLR4), and negative control NC (si-NC) were designed and synthesized by RiboBio (Guangzhou, China). For RAW264.7 cell, TLR4 siRNA (si-TLR4) and negative control NC (si-NC) were also designed and synthesized by RiboBio (Guangzhou, China). HPSCs and RAW264.7 were transfected with the siRNAs using Lipofectamine 2000 (Thermo Fisher Scientific, USA). Cells were seeded in complete medium approximately 12 h before transfection. Then, siRNAs mixed with Lipofectamine 2000 were added to the cells with fresh Opti-MEM medium (Gibco, USA). SiRNAs were transfected at a concentration of 50 nM. After 6 h, the medium containing siRNAs and Lipofectamine 2000 was replaced with complete medium. Different level of S100A8 protein (HY-P70531, MCE company, USA) was used to stimulate the HPSC cells.

### Neutrophil-panceatic stellate cell co-culture assay

For neutrophil-pancreatic stellar cell co-culture system, the schematic diagrams are shown in Figure 7F. In a 6-well transwell system, HPSCs (1 × 10^6^) were plated on the lower chamber, whereas different groups of HL-60 cells (1 × 10^6^) after DMSO induction were added to the upper chamber; the pore size was 0.4 μm. All cells were grown in RPMI 1640 supplemented with 10% FBS, 50 U/mL penicillin (Gibco, USA), and 50 μg/mL streptomycin (Gibco, USA). After 2 h of co-culture, paquinimod (HY-100442, MCE company, USA) at a dose of 10 μg/mL was added to two groups of the co-culture system. Then, each group of the HPSCs was harvested from the lower chamber after 48 h.

### Macrophage-islet cell co-culture assay

For the Macrophage-islet cell co-culture system, the schematic diagrams are shown in Fig. 7B. In a six-well transwell system, mini6 cells (1 × 10^6^) were plated on the lower chamber, whereas different groups of RAW264.7 were added to the upper chamber; the pore size was 0.4 μm. All cells were grown in DMEM (25 mmol/L glucose) supplemented with 1% PS, 10% FBS, as well as 1% β-mercaptoethanol. After 2 h of co-culture, paquinimod (HY-100442, MCE company, USA) at a dose of 10 μg/mL was added to two groups of the co-culture system. Then, each group of the mini6 cells was harvested from the lower chamber after 48 h.

### Data source and identification of diagnostic value

The blood exosomal miRNA expression of 15 osteoarthritis patients and 3 rheumatoid arthritis patients was downloaded from GSE185059 and GSE218599, respectively. To remove the impact of batch effect of the two GEO datasets and our local cohort, SVA package was used in R software^69^. Then, the miRNA value was input into the COCA algorithm, and the diagnostic value was calculated.

### scRNA-seq data processing, cluster annotation, and data integration for mouse pancreas, exosome secretion activity index inference

According to the manufacturer’s introduction, scRNA-seq libraries were constructed using Single Cell 5′ Library and Gel Bead Kit. The libraries were sequenced using an Illumina Novaseq 6000 sequencer with a sequencing depth of at least 77,618 reads per cell with a pair-end 150 bp reading strategy (performed by Oyi company, China). Chromium Single Cell 3′ Reagent v3 kits were used to prepare libraries according to the manufacturer’s protocol. Single-cell suspensions were loaded onto the Chromium Single Cell Controller Instrument (10X Genomics, Pleasanton, CA, USA) to generate single-cell gel beads in emulsions (GEMs). After the generation of GEMs, reverse transcription reactions were performed. Then, cDNA was amplified, fragmented, end-repaired, A-tailed, index adapter ligated, and subjected to library amplification. Every library was sequenced on a NovaSeq 6000 platform (Illumina, San Diego, CA, USA), and 150-bp paired-end reads were generated. The Cell Ranger software pipeline (version 3.1.0) provided by 10X Genomics was used to demultiplex cellular barcodes, map reads to the genome and transcriptome using the STAR aligner, and downsample reads as required to generate normalized aggregate data across samples, producing a matrix of gene counts versus cells^70^. Then, the three files (barcodes, features, and matrix) for each case were generated. The three files were processed through EVtrans algorithm^71^ in Python 3.9. Through “EVtras.sEV_recognizer()” and “SEVtras.ESAI_calculator()”, the exosome-related matrix in h5ad format is generated. At the same time, the matrix was analyzed using Scanpy (version 1.9.1)^72^.

We downloaded the three files (barcodes, features, and matrix) for each case and employed them to generate the object of Scanpy^72^. Considering the immune cells employed in one dataset (GSE159977), we only extracted the immune cells for further analyses. Genes expressed in fewer than three cells in a sample were excluded, as were cells that expressed fewer than 200 genes. Further quality control was performed on cells based on the number of genes expressed in the count matrix and the percentage of mitochondrial gene counts. Cells with >1000 RNA transcripts and <25,000 RNA transcripts, and mitochondrial gene counts <15% were filtered. Cells with >200 genes and <55,000 genes were filtered into the next step. We then applied the library-size correction method to normalize the data matrix using the “scanpy.pp.normalize_total” function in Scanpy. A logarithmized normalized data matrix was employed for downstream analysis.

### Dimension reduction and unsupervised clustering

Dimension reduction and unsupervised clustering were performed based on the workflow in Scanpy. We selected highly variable genes for downstream analysis by using the “scanpy.pp.highly_variable_gene” function with parameter “highly_variable_nbatches ≥ 4”. A total of 2303 highly variable genes were identified. To investigate the effect of the cell cycle, we calculated the “S_score” and “G2M_score” by using “sc.tl.score_genes_cell_cycle”. Then, the effects of total counts per cell, the percentage of mitochondrial genes expressed, the “S_score” and “G2M_score” were regressed out using the “scanpy.pp.regress_out” function. We also scaled each gene to unit variance using “scanpy.pp.scale” with parameter “max_value = 10”. After data preprocessing, we reduced the dimensionality of the data by performing a PCA. A PCA matrix was calculated to reveal the main axes of variation and denoise the data through the “scanpy.tl.pca” function with parameter “svd_solver = “arpack”, n_pcs = 11”. Furthermore, the dimensionality of merged datasets was reduced using UMAP implemented by the “scanpy.tl.umap” function. Then, to cluster the neighborhood graph of the cells, we employed the Leiden graph-clustering method. The marker genes of each cluster were identified using the “scanpy.tl.rank_genes_groups” function. The marker genes of each cluster is identified from previous single-cell stuies, specifically, C1qa and Mrc1 were used to identify macrophage, Nkg7, Cd3d, Gata3 and Il7r were used to identify T or NK cells, Prss2 and Cpa1 were used to identify Acinar cell; Sdc4, Adamtsl1, Col1a1 and Col6a1 were used to identify Fibroblast; S100A9 and S100A8 were used to identify Neutrophil; Plbd1 and Cd83 were used to identify moDC; Ighd and Ms4a1 were used to identify Naïve B cell; Jchain were used to identify Plasma; Hbb-bs were used to identify Red cell. Then, sEVtrans was used to combine the exosome data and single-cell data, and exosome secretion activity of each cell type is calculated through “SEVtras.ESAI_calculator”. Then, the data were converted from h5ad to Seurat objects. Finally, the data were visualized by Seurat V5 and SCP package (https://zhanghao-njmu.github.io/SCP/) in R software 4.0.2 (The R Foundation for Statistical Computing, Vienna, Austria).

### Schematic drawing and statistical analyses

All experimental data processing was performed using Python 3.9 (Python Software Foundation, USA), R software 4.0.2 (The R Foundation for Statistical Computing, Austria), and GraphPad Prism 8.01 software (GraphPad Company, USA). The results are shown as the mean ± SD. The significance of differences between the two groups was determined with the *t*-test and one-way analysis of variance. Significance was accepted at a value of *P* < 0.05. The schematic diagrams presented in this study were created using Biorender (available at https://app.biorender.com/), with the appropriate permissions obtained for its use.

## Supplementary information

Supplementary information

Table S10.

Table S7

Table S6

Table S5

Table S4

Table S3.

Table S2

Table S9.

## Data Availability

The whole-transcriptome sequencing data and mouse single-cell sequencing data used in the current study is publicly available in the GSA database (https://ngdc.cncb.ac.cn/), with submission numbers CRA018391 and CRA021519. Single-cell sequencing data can be acquired from public databases (http://singlecell.charite.de/cellbrowser/pancreas/?ds=Chronic_Pancreatitis). The developed COCA chronic pancreatitis classifier can be downloaded from https://gitee.com/haojiehuangSMMU/COCA-classifier-of-chronic-pancreatitis and used with R software.
