# Supplementary material for: Integrated transcriptome profiling of plasma exosomes reveals molecular stratification of exocrine and endocrine disorders and S100A8-mediated cell interactions in chronic pancreatitis
Source: Cell Discov. 2025 Nov 18;11:91. doi: 10.1038/s41421-025-00832-x (PMC12623818; doi:10.1038/s41421-025-00832-x)
Supplement: Supplementary file 1 — Supplementary information [file 41421_2025_832_MOESM1_ESM.pdf]

SUPPLEMENTARY INFORMATION

Supplementary Material and Methods

Isolation and characterized of exosomes

Exosomes were isolated from plasma samples by Exosome Isolation Reagent (CGS Exo-spin, Cell Guidance Company, USA), following the manufacturer's instructions. Then, exosomes were characterized by transmission electron microscopy (TEM), NanoSight, and western blot analysis. For NanoSight, exosomes were resuspended in 30 μL PBS (Gibco, USA). The particle size was measured by the NanoSight NS300 system (Malvern, USA). For western blot analysis, proteins of plasma exosomes were extracted by using RIPA buffer (Cell Signaling Technology, USA) and quantified by a bicinchoninic acid (BCA) protein quantification kit (Beyotime, China). Protein was separated by using 10% SDS-PAGE and electrophoretically transferred to PVDF membranes (Millipore). Membranes were incubated overnight at 4 °C with a 1:1000 dilution of primary Abs (Calnexin, Tsg101, and CD63 (No.2433, No.72312, No. 52090, Cell Signaling Technology, USA)). After incubation with a 1:1000 dilution of anti-Ig HRP-linked Ab for 1 h at room temperature, the immunoreactive bands were visualized by Pierce ECL Western Blotting Substrate (Bio-Rad, USA). For TEM, exosomes were resuspended with 50 μL PBS, then added to the paper, covered with copper mesh, and incubated at room temperature for 2 min. Then 1% acetic glaze dye was added for 30 s and observed by TEM (H7650 Hitachi, Japan).

Exosome tracing

Exosomes were stained using PKH26 dye (MIDI26-1KT, Sigma-Aldrich, USA), prepared by mixing 1 μL of the dye with 9 μL of Diluent C, followed by incubation with exosomes for 10 min at room temperature, protected from light. After centrifugation at 100,000× *g* for 17 min using an Optima MAX-XP ultracentrifuge with a TLA120.2 rotor, the exosomes from plasma of CP patients were washed three times with sterile PBS and resuspended in 100 μL of PBS. The stained exosomes were then co-incubated with adherent cells at a concentration of 15 μg/mL for 24 h. Following incubation, cells were washed, fixed with 4% paraformaldehyde for 30 min, and stained for nuclei. The samples were examined under a confocal microscope to assess exosome uptake.

Human neutrophil isolation and flow cytometry analysis

Peripheral blood samples collected from three chronic pancreatitis patients and three healthy donors were subjected to neutrophil isolation through density gradient centrifugation using Ficoll-Paque Plus (17144002, Cytiva, USA), followed by positive selection of CD16-expressing cells with magnetic-activated cell sorting (MACS) technology employing anti-human CD66b MicroBeads (130-111-552, Miltenyi Biotec,

Germany). The supernatant was then aspirated and centrifuged at 400× *g* for 5 min to obtain neutrophils. Neutrophils were extracted and stored in RPMI-1640 medium (Gibco, USA) containing 10% fetal bovine serum (FBS). The isolated neutrophils were subsequently incubated with fluorochrome-conjugated monoclonal antibodies specific for surface markers, including APC-CD45, FITC-CD66b (all from BioLegend, USA). Cellular fluorescence was quantified using a BD FACS Canto II flow cytometer (BD Biosciences, USA). Neutrophil population identification employed a dual gating strategy: primary selection based on characteristic forward scatter/side scatter (FSC/SSC) parameters, followed by confirmatory staining with anti-CD66b antibodies to ensure granulocyte lineage specificity.

Immunofluorescence and immunohistochemistry

For Immunofluorescence (IF) or immunohistochemistry (IHC), multiple antibodies are used in these experiemnts, including anti-Ly6G (ab303467, Abcam, Cambridge, UK), anti-S100A8(A1688, Abclonal company, China), anti-F4/80 (ab6640, Cambridge, UK), anti-CD206 (A26948, Abclonal company, China), anti-TNF-α (52B83, Abclonal company, China), IL-1(A1316, Abclonal company, China), IL-6(A21264, Abclonal company, China) and anti-Insulin(ab181547, Abcam, Cambridge, UK). The antibodies were used to detect the cell markers. Then, the slides of the paraffin block were incubated with multiple antibodies and counterstained with 4',6-diamidino-2-phenylindole (DAPI) (ab228549, Abcam, Cambridge, UK). Finally, the slides were observed under a fluorescence microscope.

Real-time quantitative PCR

For exosome from plasma and cell experiment related to qPCR of miRNA, miRNA 1st Strand cDNA Synthesis Kit (Yeasen company, Shanghai, China) was used to perform genomic DNA removal, cDNA synthesis, qPCR reaction, following manufacturer's instructions. The primer sequence is listed in Supplementary Table S9. For cell experiments, cells were suspended in Trizol reagent (Invitrogen, USA), and the total RNA was extracted according to the manufacturer's instructions. Reverse transcription was performed using a Reverse Transcription Kit (Toyobo, Osaka, Japan). A SYBRGreen RT-PCR Kit from Toyobo was used for quantitative real-time PCR analysis with the Step One PLUS Real-time PCRsystem (Applied Biosystems, USA), according to the manufacturer's instructions. Gene-specific primers were used to detect human S100A8 (forward primer:5'-CCAGGAGUUCCUCAUUCUGTT3-3' 5'-CAGAAUGAGGAACUCCUGGTT-3'), mouse Il1b (forward primer: 5-CAACCAACAAGTGATATTCTCCATG-3; reverse primer: 5-GATCCACACTCTCCAGCTGCA-3) , mouse Il6 (forward primer: 5-ACCTGTCTATACCACTTCACAAGT-3; reverse primer: 5-

TCTGCAAGTGCATCATCGTTGTTC-3), mouse Tnfa (forward primer: 5-CCAGACCCTCACACTCAGATC-3; reverse primer: 5-AGTTGGTTGTCTTTGAGATCCATG-3), mouse S100A8 (forward primer: 5-CAAGGAAATCACCATGCCCTCTA; reverse primer: 5-ACCATCGCAAGGAACTCCTCGA-3) and fold changes were calculated using the formula $2^{-\triangle Ct}$.

Luciferase reporter assays

293T cells were seeded into 96-well plates 24 h before transfection. Subsequently, the cells were transiently co-transfected with 0.16 ng wild-type or mutant reporter plasmid (Hanbio company, China) and 5 pmol miR-24b-3p or negative control using Lipofectamine 2000 (Thermo Fisher, USA). Firefly luciferase activities were measured 48 h after transfection using the Dual Luciferase Assay (Promega, Madison, USA), according to the manufacturer's protocol. Firefly luciferase activity was normalized to Renilla luciferase activity and the ratio was analyzed.

Intracellular ROS assay

Cell climbing sheets were prepared, and the cells were subsequently cultured in treated medium. Afterwards, the old medium was discarded and replaced with fresh medium infused with 0.1% 2′, 7′-dichlorofluorescin diacetate (DCFH-DA). These sheets were then incubated at 37 °C, shielded from light, for 20 minutes. To finalize the process, the cells adhering to the sheets were washed three times with PBS, and images were captured using a fluorescence microscope.

Mice and CP model

Six-week-old C57BL/6 J mice were purchased from Shanghai JieSiJie Laboratory Animal Co. Ltd. Mice model of chronic pancreatitis was induced by repeated intraperitoneal injection of caerulein (60321ES03, Yeasen, China). The procedure is shown in Figure 6k. Briefly, six-week-old mice were injected intraperitoneally with caerulein three times a week on alternate days for 6 consecutive weeks, at a dose of 50 μg/ kg. Mice were sacrificed 3 days after the last injection of caerulein. For paquinimod treatment, the treatment groups of chronic pancreatitis mice were administered by gavage every day at a dose of 5 mg/kg paquinimod (HY-100442, MCE company, USA) from the 3rd week until the mice were sacrificed, respectively, and the control or CP mice were given equal amounts of solvent (10% DMSO dissolved in 90% saline)).

Pancreas Collection

Mice were euthanized by CO2 inhalation followed by cervical dislocation. The abdominal cavity was opened, and the pancreas was carefully excised. The tissue was

immediately washed in ice-cold phosphate-buffered saline (PBS) to remove excess blood and then snap-frozen in liquid nitrogen for further analysis or fixed in 10% formalin for histological examination.

Blood and Plasma Collection

Blood samples were collected via cardiac puncture using a 1 mL syringe with a 25-gauge needle. The blood was transferred to EDTA-coated tubes and centrifuged at 1,500 x g for 15 minutes at 4 °C to separate the plasma. The plasma was carefully aspirated and stored at -80 °C until further use.

Exosome Isolation from Plasma

Exosomes were isolated from the plasma using a differential centrifugation method. Briefly, the plasma was first centrifuged at 2,000 x g for 10 minutes at 4 °C to remove cells and debris. The supernatant was then centrifuged at 10,000 x g for 30 minutes at 4 °C to pellet larger vesicles. Subsequently, the supernatant was filtered through a 0.22 μm filter to remove any remaining large particles. The filtered plasma was then ultracentrifuged at 100,000 x g for 70 minutes at 4 °C to pellet the exosomes. The exosome pellet was washed once with PBS and centrifuged again at 100,000 x g for 70 minutes. The final exosome pellet was resuspended in PBS and stored at -80 °C for further analysis.

Antibody-mediated neutrophil depletion

For deplete neutrophils in mice, the method is referred to previous papers (Gr-1+ Cells Other Than Ly6G$^+$ Neutrophils Limit Virus Replication and Promote Myocardial Inflammation and Fibrosis Following Coxsackievirus B3 Infection of Mice). Specifically, to analyze the RNA expression of S100a8 and miR-24-3p in exosome with or without neutrophils in mouse model of chronic pancreatitis, 12.5 mg/Kg of anti-Ly-6G (clone 1A8, Biolegend, USA) and isotype control (rat IgG2a, Biolegend, USA) Abs in 100 μl PBS were injected i.p. into chronic pancreatitis mice (n=5) at the last time of caerulein injection and 4 days after the last time of caerulein injection. Blood was collected by saphenous venous puncture. Total white blood cell counts (WBC) were determined by visual enumeration after trypan blue exclusion. The percentage of neutrophils was determined by flow cytometry using antibody cocktails (anti-CD45-APC/Cy7, anti-CD11b-FITC, and anti-Ly6G-PE/Cy7, BD PharMingen, USA). Fluorescent intensity was determined using a FACS CantoII flow cytometer (BD Bioscience, USA) and the data were analyzed using FlowJo v10.0 software. Ethical approval for this research was granted by the Animal Ethics Committee of Changhai Hospital (CHEC2021-283-M).

Tissue digestion

Complete media was prepared with RPMI-1640 (Hyclone, USA), 10% FBS (Gibco, USA), and 1% penicillin-streptomycin (Hyclone, USA). Mice pancreas were each minced with scissors and enzymatically digested in complete media supplemented with 1.0 mg/ml collagenase type IV (Sigma, USA), 30 U/ml DNase type I (Sigma, USA), and 0.5 mg/ml HAase type V (Sigma, USA) for 50 min at 37 °C. Then the cells were filtered through the 70 μm cell strainers (Miltenyi Biotec, Germany), washed with PBS, lysed in red blood cell buffer (BioTeke, China), and resuspended in PBS.

Histology

Fresh pancreas was taken immediately and fixed in 4% paraformaldehyde for 24 h. HE, and Masson staining were performed according to the standard protocols in Yuxiu biotechnology (Shanghai, China).

Western blot

RIPA buffers were used to extract total protein from cells or pancreatic tissue, and BCA protein assay kit was used to determine total protein concentration. The same mass of protein is added to the sample tank and then subjected to SDS-polyacrylamide gel electrophoresis. All the protein was transferred to polyvinylidene difluoride membranes and blocked with 3% BSA for 1h at room temperature. They are then incubated with an anti-RAGE(catalog #ab216329, Abcam, USA), anti-CD36(catalog # ab252922, Abcam, USA), anti-TLR4(catalog # AF7017, Affinity, China), anti-S100A8 (catalog # ab92331, Affinity, China), anti-FN(catalog # sc-8422, Santa Cruz, USA), anti-Collagen-1(catalog # 72026, Cell Signaling Technology, USA), anti-α-tublin (catalog # ab176560, Cell Signaling Technology, USA), anti-Bcl-2 (catalog # ab182858, Cell Signaling Technology, USA), anti-Bax (catalog # ab32503, Cell Signaling Technology, USA) , anti-GAPDH(catalog # ab8245, Cell Signaling Technology, USA). The gray value of the detected bands was quantified by using ImageJ software. The experiment was repeated three times.

Supplementary tables

Table S1. The characteristics of enrolled patients and healthy donor for EV-sequencing.

	Patient group		
Characteristics	Normal(n=22)	CP(n=89)	P value
Age, y	48.4±8.73	45.7±16.22	0.64
Sex			0.42
Female	9	39	

Male	13	50	
BMI	23.7±1.48	20.8±3.9	<0.001
Diabetes mellitus	0	27	<0.001
Steatorrhea	0	25	<0.001
Smoking status			<0.001
Never	11	38	
Past	3	16	
Current	8	35	
Drink status			<0.001
Never	9	47	
Past	4	16	
Current	9	26	
Clinical stages of chronic pancreatitis*			
Stage 0		9	
Stage I		42	
Stage II		24	
Stage III		8	
Stage IV		6	
Course of chronic pancreatitis(month)			<0.001
Stage 0		58.7±54.43	
Stage I		53.7±50.03	
Stage II		116.5±237.86	
Stage III		121.5±88.11	
Stage IV		79±31.89	

*Based on the "Zou WB, Ru N, Wu H, Hu LH, Ren X, Jin G, et al. Guidelines for the diagnosis and treatment of chronic pancreatitis in China (2018 edition). Hepatobiliary & pancreatic diseases international : HBPD INT. 2019;18(2):103-9."

Table S2. The differentially expressed genes between CP patients and healthy donors.

Please see Table S2.txt

Table S3. The differentially expressed miRNA between CP patients and healthy donors.

Please see Table S3.txt

Table S4. The differentially expressed lncRNA between CP patients and healthy donors.

Please see Table S4.txt

Table S5. The differentially expressed circRNA between CP patients and healthy donors.

Please see Table S5.txt

Table S6. The marker genes in exocrine signature and their expression in acinar REG+ cells and activated stellate cells.

Please see Table S6.txt

Table S7. The marker genes in endocrine signature and their expression in alpha cell or beta cells.

Please see Table S7.txt

Table S8. The characteristics of enrolled patients in each cluster (COCA1-COCA3).

Characteristics	COCA1(n=34)	COCA2(n=24)	COCA3(n=31)	P value
Age, y	46.32±16.39	43.42±18.78	46.87±14.43	0.46
Sex				0.12
Female	14	10	15	
Male	20	14	16	
BMI	22.04±2.72	19.71±5.92	20.36±2.71	0.033
Diabetes mellitus	5	6	16	<0.001
Steatorrhea	3	8	14	<0.001
Smoking status				0.006
Never	16	10	12	
Past	4	4	8	
Current	14	10	11	
Drink status				0.003
Never	17	12	18	
Past	7	4	5	
Current	10	8	8	
Course of chronic pancreatitis(month)	63.4±50.79	112.7±271.68	81.5±75.0	<0.001
Clinical stages of chronic pancreatitis				
Stage 0	4	3	2	
Stage I	23	10	9	
Stage II	6	8	10	
Stage III	1	1	6	
Stage IV	0	2	4	

Table S9. The potential miRNA–mRNA interaction networks with Experimental Evidences downloaded from the miRTarBase.

Please see Table S9.xlsx

Table S10. The miRNA primer name and primer sequence involved in this study.

Please see Table S10.txt

Table S11. The characteristics of enrolled patients and healthy donor for validation through EVs qPCR.

	Patient group		
Characteristics	Normal(n=10)	CP(n=90)	P value
Age, y	46.6±12.72	48.1±12.05	0.76
Sex			0.38
Female	5	44	
Male	5	46	
BMI	23.7±1.48	21.6±2.04	0.11
Diabetes mellitus	0	25	<0.001
Steatorrhea	0	23	<0.001
Smoking status			<0.001
Never	5	60	
Past	0	5	
Current	5	25	
Drink status			<0.001
Never	6	45	
Past	1	34	
Current	3	11	
Clinical stages of chronic pancreatitis			
Stage 0		20	
Stage I		36	
Stage II		20	
Stage III		8	
Stage IV		6	
Course of chronic pancreatitis(month)			<0.001
Stage 0		52.3±45.83	
Stage I		77.3±42.3	
Stage II		46.8±57.63	
Stage III		63.5±48.11	
Stage IV		59±41.28	

Table S12. The characteristics of validation-group patients in each cluster (COCA1-COCA3).

	Patient group			
Characteristics	COCA1(n=48)	COCA2(n=16)	COCA3(n=26)	P value
Age, y	54±11	42.9±12.6	50.85±11.49	0.023
Sex				0.286
Female	24	10	10	
Male	24	6	16	

BMI	23.62±2.52	22.35±3.86	20.93±2.39	0.045
Diabetes mellitus	3	7	15	<0.001
Steatorrhea	2	6	15	<0.001
Smoking status				0.041
Never	35	8	17	
Past	3	1	1	
Current	10	7	8	
Drink status				0.181
Never	20	6	19	
Past	17	7	10	
Current	6	3	2	
Course of chronic pancreatitis(month)	72.2±48.28	59.8±41.91	61.5±54.6	<0.001
Clinical stages of chronic pancreatitis				
Stage 0	16	2	2	
Stage I	28	4	4	
Stage II	3	7	10	
Stage III	1	1	6	
Stage IV	0	2	4	

Table S13. Diagnostic performance of the COCA algorithm for distinguishing chronic pancreatitis from other diseases.

Actual \ Predicted	Chronic pancreatitis	Other disease	Total
**Chronic Pancreatitis**	78 (TP)	11 (FN)	89
**Other Diseases**	4 (FP)	14 (TN)	18
**Total**	82	25	107

Sensitivity=*TP+FNTP*=78+1178≈87.6%

Specificity=*TN+FPTN*=14+414≈77.8%

Accuracy=Total*TP+TN*=10778+14≈86.0%

TP (True Positive): Correctly diagnosed CP cases.

FP (False Positive): Non-CP cases misclassified as CP.

FN (False Negative): CP cases missed by the algorithm.

TN (True Negative): Correctly excluded non-CP cases.

Data are presented as counts (n). Total sample size = 107 (89 CP, 18 other diseases).

Table S14 The potential mRNA-miRNA interaction matches in these 12 miRNAs from the current COCA diagnostic classifier.

miRTarBase.ID	miRNA	Species.(miRNA)	Target.Gene	GeneID
MIRT179447	hsa-miR-6134	Homo sapiens	TBRG1	
MIRT188339	hsa-miR-6134	Homo sapiens	ARID1A	
MIRT200724	hsa-miR-6134	Homo sapiens	ZNF473	
MIRT210190	hsa-miR-6134	Homo sapiens	ARIH2	
MIRT264224	hsa-miR-6134	Homo sapiens	DFFA	
MIRT336246	hsa-miR-6134	Homo sapiens	SKI	
MIRT405443	hsa-miR-6134	Homo sapiens	RAB5B	
MIRT450002	hsa-miR-6134	Homo sapiens	HAX1	
MIRT450052	hsa-miR-6134	Homo sapiens	IL17RA	
MIRT451102	hsa-miR-6134	Homo sapiens	ZNF584	
MIRT451573	hsa-miR-6134	Homo sapiens	CIAPIN1	
MIRT451589	hsa-miR-6134	Homo sapiens	HIRIP3	

MIRT451764	hsa-miR-6134	Homo sapiens	ZNF611
MIRT452578	hsa-miR-6134	Homo sapiens	ZFP69B
MIRT452869	hsa-miR-6134	Homo sapiens	LAX1
MIRT453077	hsa-miR-6134	Homo sapiens	SUMF2
MIRT453260	hsa-miR-6134	Homo sapiens	PARP11
MIRT453453	hsa-miR-6134	Homo sapiens	GLG1
MIRT453481	hsa-miR-6134	Homo sapiens	PITPNM3
MIRT453725	hsa-miR-6134	Homo sapiens	RAP1GDS1
MIRT454185	hsa-miR-6134	Homo sapiens	AP1S3
MIRT454431	hsa-miR-6134	Homo sapiens	GTF2F1
MIRT454667	hsa-miR-6134	Homo sapiens	FBXL18
MIRT454829	hsa-miR-6134	Homo sapiens	POLR2J3
MIRT454987	hsa-miR-6134	Homo sapiens	RECK
MIRT455133	hsa-miR-6134	Homo sapiens	TBC1D25
MIRT455838	hsa-miR-6134	Homo sapiens	MPL
MIRT456003	hsa-miR-6134	Homo sapiens	CYP2C19
MIRT456450	hsa-miR-6134	Homo sapiens	TMEM81
MIRT457160	hsa-miR-6134	Homo sapiens	MXRA7
MIRT457459	hsa-miR-6134	Homo sapiens	UNC119B
MIRT457623	hsa-miR-6134	Homo sapiens	UPK3BL1
MIRT457693	hsa-miR-6134	Homo sapiens	ZNF587
MIRT457794	hsa-miR-6134	Homo sapiens	VWA1
MIRT457912	hsa-miR-6134	Homo sapiens	ZNF212
MIRT458348	hsa-miR-6134	Homo sapiens	NOC2L
MIRT458397	hsa-miR-6134	Homo sapiens	ABCF1
MIRT458414	hsa-miR-6134	Homo sapiens	RNF185
MIRT458501	hsa-miR-6134	Homo sapiens	MARVELD2
MIRT459348	hsa-miR-6134	Homo sapiens	ZNF17
MIRT459370	hsa-miR-6134	Homo sapiens	MPLKIP
MIRT459576	hsa-miR-6134	Homo sapiens	NLGN2
MIRT459988	hsa-miR-6134	Homo sapiens	RFT1
MIRT460036	hsa-miR-6134	Homo sapiens	CDCP1
MIRT460800	hsa-miR-6134	Homo sapiens	VPS33A
MIRT461031	hsa-miR-6134	Homo sapiens	SDF4
MIRT461060	hsa-miR-6134	Homo sapiens	KCNK6
MIRT461575	hsa-miR-6134	Homo sapiens	SCO1
MIRT461957	hsa-miR-6134	Homo sapiens	C3
MIRT462282	hsa-miR-6134	Homo sapiens	KRR1
MIRT462748	hsa-miR-6134	Homo sapiens	EFNB1
MIRT462912	hsa-miR-6134	Homo sapiens	ZNRF3
MIRT463137	hsa-miR-6134	Homo sapiens	ZNF451
MIRT463940	hsa-miR-6134	Homo sapiens	WIZ
MIRT464327	hsa-miR-6134	Homo sapiens	UST
MIRT464797	hsa-miR-6134	Homo sapiens	UBE2F

MIRT464994	hsa-miR-6134	Homo sapiens	TUBB2A
MIRT465858	hsa-miR-6134	Homo sapiens	TMEM50B
MIRT467350	hsa-miR-6134	Homo sapiens	SP2
MIRT467662	hsa-miR-6134	Homo sapiens	SLC5A6
MIRT468035	hsa-miR-6134	Homo sapiens	SIKE1
MIRT468578	hsa-miR-6134	Homo sapiens	SERBP1
MIRT468971	hsa-miR-6134	Homo sapiens	RPRD2
MIRT470109	hsa-miR-6134	Homo sapiens	PTBP2
MIRT472591	hsa-miR-6134	Homo sapiens	NACC1
MIRT472822	hsa-miR-6134	Homo sapiens	MTMR10
MIRT472869	hsa-miR-6134	Homo sapiens	MTHFD2
MIRT472918	hsa-miR-6134	Homo sapiens	MSN
MIRT473475	hsa-miR-6134	Homo sapiens	MCFD2
MIRT474350	hsa-miR-6134	Homo sapiens	KMT2D
MIRT474560	hsa-miR-6134	Homo sapiens	KLHDC3
MIRT474819	hsa-miR-6134	Homo sapiens	RUBCN
MIRT475281	hsa-miR-6134	Homo sapiens	TOR1AIP2
MIRT475763	hsa-miR-6134	Homo sapiens	HDLBP
MIRT475788	hsa-miR-6134	Homo sapiens	HDGF
MIRT476214	hsa-miR-6134	Homo sapiens	GNS
MIRT476309	hsa-miR-6134	Homo sapiens	GM2A
MIRT476805	hsa-miR-6134	Homo sapiens	FNDC3B
MIRT476959	hsa-miR-6134	Homo sapiens	FAM83G
MIRT478816	hsa-miR-6134	Homo sapiens	CRTAP
MIRT478851	hsa-miR-6134	Homo sapiens	CRISPLD2
MIRT479302	hsa-miR-6134	Homo sapiens	CFL2
MIRT480323	hsa-miR-6134	Homo sapiens	C5orf51
MIRT480711	hsa-miR-6134	Homo sapiens	BRPF1
MIRT482101	hsa-miR-6134	Homo sapiens	AKT3
MIRT482393	hsa-miR-6134	Homo sapiens	AEN
MIRT482585	hsa-miR-6134	Homo sapiens	ABHD2
MIRT482707	hsa-miR-6134	Homo sapiens	XRCC3
MIRT482770	hsa-miR-6134	Homo sapiens	ANKHD1-EIF4EBP3
MIRT483367	hsa-miR-6134	Homo sapiens	CYP4A22
MIRT483609	hsa-miR-6134	Homo sapiens	GPX7
MIRT484056	hsa-miR-6134	Homo sapiens	CYP4A11
MIRT484096	hsa-miR-6134	Homo sapiens	EIF4EBP3
MIRT484344	hsa-miR-6134	Homo sapiens	EPN1
MIRT484473	hsa-miR-6134	Homo sapiens	DDX6
MIRT486652	hsa-miR-6134	Homo sapiens	ZNF28
MIRT489782	hsa-miR-6134	Homo sapiens	GRINA
MIRT490538	hsa-miR-6134	Homo sapiens	LNPK
MIRT492286	hsa-miR-6134	Homo sapiens	SHISA6
MIRT493879	hsa-miR-6134	Homo sapiens	MIGA2

MIRT497546	hsa-miR-6134	Homo sapiens	SLC13A4
MIRT498861	hsa-miR-6134	Homo sapiens	LITAF
MIRT500056	hsa-miR-6134	Homo sapiens	S100A11
MIRT501608	hsa-miR-6134	Homo sapiens	PLAGL2
MIRT503194	hsa-miR-6134	Homo sapiens	ACVR2B
MIRT507986	hsa-miR-6134	Homo sapiens	BCL2L13
MIRT508389	hsa-miR-6134	Homo sapiens	SPTBN2
MIRT508464	hsa-miR-6134	Homo sapiens	HOXB6
MIRT509558	hsa-miR-6134	Homo sapiens	ACTG1
MIRT511112	hsa-miR-6134	Homo sapiens	NFIB
MIRT512228	hsa-miR-6134	Homo sapiens	ATXN3
MIRT513798	hsa-miR-6134	Homo sapiens	NIPAL3
MIRT514188	hsa-miR-6134	Homo sapiens	PGPEP1
MIRT514914	hsa-miR-6134	Homo sapiens	FTO
MIRT515140	hsa-miR-6134	Homo sapiens	ZNF799
MIRT515454	hsa-miR-6134	Homo sapiens	ZNF747
MIRT515906	hsa-miR-6134	Homo sapiens	AGTPBP1
MIRT516132	hsa-miR-6134	Homo sapiens	MRPS16
MIRT516681	hsa-miR-6134	Homo sapiens	ZNF860
MIRT516752	hsa-miR-6134	Homo sapiens	ZNF100
MIRT516974	hsa-miR-6134	Homo sapiens	OR7D2
MIRT517494	hsa-miR-6134	Homo sapiens	NPAP1
MIRT517775	hsa-miR-6134	Homo sapiens	PROM2
MIRT517940	hsa-miR-6134	Homo sapiens	ZNF431
MIRT518389	hsa-miR-6134	Homo sapiens	ZNF250
MIRT518498	hsa-miR-6134	Homo sapiens	FAM161B
MIRT519273	hsa-miR-6134	Homo sapiens	ZNF141
MIRT520622	hsa-miR-6134	Homo sapiens	TMEM41B
MIRT521030	hsa-miR-6134	Homo sapiens	SLC30A5
MIRT521407	hsa-miR-6134	Homo sapiens	RDH11
MIRT521566	hsa-miR-6134	Homo sapiens	HACD2
MIRT522917	hsa-miR-6134	Homo sapiens	KCNE3
MIRT523033	hsa-miR-6134	Homo sapiens	IGF1
MIRT524678	hsa-miR-6134	Homo sapiens	TIGAR
MIRT530750	hsa-miR-6134	Homo sapiens	GPR82
MIRT532731	hsa-miR-6134	Homo sapiens	POLR2E
MIRT540694	hsa-miR-6134	Homo sapiens	BMP3
MIRT540980	hsa-miR-6134	Homo sapiens	NCBP3
MIRT545086	hsa-miR-6134	Homo sapiens	MRPL58
MIRT545635	hsa-miR-6134	Homo sapiens	GGCX
MIRT549395	hsa-miR-6134	Homo sapiens	AKIRIN1
MIRT550719	hsa-miR-6134	Homo sapiens	PMPCA
MIRT551207	hsa-miR-6134	Homo sapiens	NCR3LG1
MIRT552613	hsa-miR-6134	Homo sapiens	ZBTB8A

MIRT561020	hsa-miR-6134	Homo sapiens	LIN7C
MIRT561085	hsa-miR-6134	Homo sapiens	LLPH
MIRT565237	hsa-miR-6134	Homo sapiens	TRAF6
MIRT566623	hsa-miR-6134	Homo sapiens	NKAP
MIRT569529	hsa-miR-6134	Homo sapiens	AP5Z1
MIRT570254	hsa-miR-6134	Homo sapiens	SSPN
MIRT570576	hsa-miR-6134	Homo sapiens	OTUD7B
MIRT570728	hsa-miR-6134	Homo sapiens	CELSR2
MIRT608272	hsa-miR-6134	Homo sapiens	NOP14
MIRT626722	hsa-miR-6134	Homo sapiens	TRIM65
MIRT627675	hsa-miR-6134	Homo sapiens	RPL28
MIRT638122	hsa-miR-6134	Homo sapiens	WIPF2
MIRT643285	hsa-miR-6134	Homo sapiens	TRAPPC2
MIRT661487	hsa-miR-6134	Homo sapiens	CHMP1B
MIRT663000	hsa-miR-6134	Homo sapiens	TMEM59
MIRT665730	hsa-miR-6134	Homo sapiens	TMTC1
MIRT668963	hsa-miR-6134	Homo sapiens	CNBP
MIRT670926	hsa-miR-6134	Homo sapiens	DESI1
MIRT672836	hsa-miR-6134	Homo sapiens	PTPN3
MIRT673944	hsa-miR-6134	Homo sapiens	ZNF500
MIRT674680	hsa-miR-6134	Homo sapiens	PLCE1
MIRT675235	hsa-miR-6134	Homo sapiens	MAK
MIRT677880	hsa-miR-6134	Homo sapiens	ABI2
MIRT678189	hsa-miR-6134	Homo sapiens	CRCP
MIRT680166	hsa-miR-6134	Homo sapiens	ZDHHC20
MIRT680869	hsa-miR-6134	Homo sapiens	MACC1
MIRT681051	hsa-miR-6134	Homo sapiens	ZDBF2
MIRT681109	hsa-miR-6134	Homo sapiens	CEP57L1
MIRT684511	hsa-miR-6134	Homo sapiens	C1orf174
MIRT685059	hsa-miR-6134	Homo sapiens	GEMIN4
MIRT685391	hsa-miR-6134	Homo sapiens	TNFRSF13C
MIRT685821	hsa-miR-6134	Homo sapiens	SLC27A1
MIRT685985	hsa-miR-6134	Homo sapiens	CCDC77
MIRT686710	hsa-miR-6134	Homo sapiens	TBC1D19
MIRT686872	hsa-miR-6134	Homo sapiens	SLC25A32
MIRT688575	hsa-miR-6134	Homo sapiens	DARS2
MIRT689523	hsa-miR-6134	Homo sapiens	ORC6
MIRT692301	hsa-miR-6134	Homo sapiens	CNNM3
MIRT693105	hsa-miR-6134	Homo sapiens	SCNM1
MIRT694383	hsa-miR-6134	Homo sapiens	MTA1
MIRT694465	hsa-miR-6134	Homo sapiens	LRTOMT
MIRT694747	hsa-miR-6134	Homo sapiens	LLGL1
MIRT694839	hsa-miR-6134	Homo sapiens	KRT80
MIRT695228	hsa-miR-6134	Homo sapiens	SCAMP3

MIRT695774	hsa-miR-6134	Homo sapiens	DENR
MIRT696500	hsa-miR-6134	Homo sapiens	COX6B1
MIRT698652	hsa-miR-6134	Homo sapiens	TERF2
MIRT700339	hsa-miR-6134	Homo sapiens	RAB4A
MIRT701052	hsa-miR-6134	Homo sapiens	PARP2
MIRT702069	hsa-miR-6134	Homo sapiens	MED28
MIRT703378	hsa-miR-6134	Homo sapiens	GAPVD1
MIRT703613	hsa-miR-6134	Homo sapiens	FBXO45
MIRT703711	hsa-miR-6134	Homo sapiens	RETREG2
MIRT704897	hsa-miR-6134	Homo sapiens	CCNB1
MIRT706190	hsa-miR-6134	Homo sapiens	SAR1B
MIRT706649	hsa-miR-6134	Homo sapiens	SMIM19
MIRT708849	hsa-miR-6134	Homo sapiens	ZBED9
MIRT709711	hsa-miR-6134	Homo sapiens	DNAJC11
MIRT710694	hsa-miR-6134	Homo sapiens	LYRM4
MIRT718108	hsa-miR-6134	Homo sapiens	CRTC1
MIRT752406	hsa-miR-6134	Homo sapiens	ACBD5
MIRT752407	hsa-miR-6134	Homo sapiens	ADAMTSL1
MIRT752408	hsa-miR-6134	Homo sapiens	ADIRF
MIRT752409	hsa-miR-6134	Homo sapiens	AMBRA1
MIRT752410	hsa-miR-6134	Homo sapiens	ANKRD52
MIRT752411	hsa-miR-6134	Homo sapiens	ANXA6
MIRT752412	hsa-miR-6134	Homo sapiens	ARPP19
MIRT752413	hsa-miR-6134	Homo sapiens	ATP8B2
MIRT752414	hsa-miR-6134	Homo sapiens	CBS
MIRT752415	hsa-miR-6134	Homo sapiens	CHD4
MIRT752416	hsa-miR-6134	Homo sapiens	CPSF7
MIRT752417	hsa-miR-6134	Homo sapiens	CRY2
MIRT752419	hsa-miR-6134	Homo sapiens	DUSP4
MIRT752420	hsa-miR-6134	Homo sapiens	ELL
MIRT752421	hsa-miR-6134	Homo sapiens	EPN2
MIRT752422	hsa-miR-6134	Homo sapiens	EVI5
MIRT752423	hsa-miR-6134	Homo sapiens	FAT3
MIRT752425	hsa-miR-6134	Homo sapiens	FGFR1
MIRT752426	hsa-miR-6134	Homo sapiens	FOXK1
MIRT752427	hsa-miR-6134	Homo sapiens	GPS1
MIRT752428	hsa-miR-6134	Homo sapiens	HIC2
MIRT752429	hsa-miR-6134	Homo sapiens	HNRNPA0
MIRT752430	hsa-miR-6134	Homo sapiens	HP1BP3
MIRT752431	hsa-miR-6134	Homo sapiens	JUNB
MIRT752432	hsa-miR-6134	Homo sapiens	KIAA0513
MIRT752433	hsa-miR-6134	Homo sapiens	KMT2A
MIRT752434	hsa-miR-6134	Homo sapiens	LDHA
MIRT752435	hsa-miR-6134	Homo sapiens	LNX2

MIRT752436	hsa-miR-6134	Homo sapiens	LUZP1
MIRT752437	hsa-miR-6134	Homo sapiens	MAOB
MIRT752438	hsa-miR-6134	Homo sapiens	MEF2D
MIRT752439	hsa-miR-6134	Homo sapiens	MRO
MIRT752440	hsa-miR-6134	Homo sapiens	MRPL44
MIRT752441	hsa-miR-6134	Homo sapiens	MSI2
MIRT752442	hsa-miR-6134	Homo sapiens	NFIC
MIRT752443	hsa-miR-6134	Homo sapiens	NLE1
MIRT752444	hsa-miR-6134	Homo sapiens	NPR1
MIRT752445	hsa-miR-6134	Homo sapiens	NR1H2
MIRT752446	hsa-miR-6134	Homo sapiens	NUGGC
MIRT752447	hsa-miR-6134	Homo sapiens	NUP62
MIRT752448	hsa-miR-6134	Homo sapiens	P4HB
MIRT752449	hsa-miR-6134	Homo sapiens	PAPOLG
MIRT752450	hsa-miR-6134	Homo sapiens	PDCL3
MIRT752451	hsa-miR-6134	Homo sapiens	PGBD4
MIRT752452	hsa-miR-6134	Homo sapiens	PHACTR4
MIRT752453	hsa-miR-6134	Homo sapiens	PHLDA3
MIRT752454	hsa-miR-6134	Homo sapiens	PIGW
MIRT752455	hsa-miR-6134	Homo sapiens	PISD
MIRT752456	hsa-miR-6134	Homo sapiens	PLD3
MIRT752457	hsa-miR-6134	Homo sapiens	PLEKHB2
MIRT752458	hsa-miR-6134	Homo sapiens	PLEKHM1
MIRT752459	hsa-miR-6134	Homo sapiens	PLXNA1
MIRT752460	hsa-miR-6134	Homo sapiens	PPP2R1A
MIRT752462	hsa-miR-6134	Homo sapiens	PSAP
MIRT752463	hsa-miR-6134	Homo sapiens	PTPN1
MIRT752464	hsa-miR-6134	Homo sapiens	PTPN6
MIRT752465	hsa-miR-6134	Homo sapiens	SCAMP4
MIRT752466	hsa-miR-6134	Homo sapiens	SETDB1
MIRT752468	hsa-miR-6134	Homo sapiens	SLC9A7
MIRT752469	hsa-miR-6134	Homo sapiens	STK35
MIRT752470	hsa-miR-6134	Homo sapiens	SUPT4H1
MIRT752471	hsa-miR-6134	Homo sapiens	TFAP4
MIRT752472	hsa-miR-6134	Homo sapiens	TUBB
MIRT752473	hsa-miR-6134	Homo sapiens	WBP2
MIRT752474	hsa-miR-6134	Homo sapiens	ZFP36
MIRT752475	hsa-miR-6134	Homo sapiens	ZMIZ2
MIRT752476	hsa-miR-6134	Homo sapiens	ZNF251
MIRT775922	hsa-miR-6134	Homo sapiens	ABCF3
MIRT775923	hsa-miR-6134	Homo sapiens	ACOX1
MIRT775927	hsa-miR-6134	Homo sapiens	BMPR1A
MIRT775928	hsa-miR-6134	Homo sapiens	CEP97
MIRT775929	hsa-miR-6134	Homo sapiens	CLN8

MIRT775930	hsa-miR-6134	Homo sapiens	CYTH2
MIRT775931	hsa-miR-6134	Homo sapiens	DNAL1
MIRT775932	hsa-miR-6134	Homo sapiens	DYNC1LI2
MIRT775933	hsa-miR-6134	Homo sapiens	ESCO2
MIRT775935	hsa-miR-6134	Homo sapiens	FBXO48
MIRT775936	hsa-miR-6134	Homo sapiens	FDXACB1
MIRT775937	hsa-miR-6134	Homo sapiens	FUT1
MIRT775938	hsa-miR-6134	Homo sapiens	GABARAP
MIRT775939	hsa-miR-6134	Homo sapiens	GDPGP1
MIRT775940	hsa-miR-6134	Homo sapiens	GIPC1
MIRT775941	hsa-miR-6134	Homo sapiens	GLP2R
MIRT775942	hsa-miR-6134	Homo sapiens	HACE1
MIRT775943	hsa-miR-6134	Homo sapiens	HARBI1
MIRT775944	hsa-miR-6134	Homo sapiens	HSD17B12
MIRT775946	hsa-miR-6134	Homo sapiens	IMP4
MIRT775948	hsa-miR-6134	Homo sapiens	LAT2
MIRT775950	hsa-miR-6134	Homo sapiens	LYZ
MIRT775951	hsa-miR-6134	Homo sapiens	MAN1C1
MIRT775952	hsa-miR-6134	Homo sapiens	MDM2
MIRT775953	hsa-miR-6134	Homo sapiens	NEURL4
MIRT775954	hsa-miR-6134	Homo sapiens	NFAT5
MIRT775955	hsa-miR-6134	Homo sapiens	NUDT19
MIRT775956	hsa-miR-6134	Homo sapiens	OPRK1
MIRT775957	hsa-miR-6134	Homo sapiens	PKNOX1
MIRT775958	hsa-miR-6134	Homo sapiens	PLEKHG3
MIRT775959	hsa-miR-6134	Homo sapiens	PPP1R12B
MIRT775960	hsa-miR-6134	Homo sapiens	PRPS1
MIRT775961	hsa-miR-6134	Homo sapiens	QTRT2
MIRT775962	hsa-miR-6134	Homo sapiens	RBSN
MIRT775963	hsa-miR-6134	Homo sapiens	RC3H1
MIRT775964	hsa-miR-6134	Homo sapiens	RHOF
MIRT775965	hsa-miR-6134	Homo sapiens	RNF40
MIRT775966	hsa-miR-6134	Homo sapiens	SLC25A16
MIRT775967	hsa-miR-6134	Homo sapiens	SLC41A2
MIRT775968	hsa-miR-6134	Homo sapiens	SNTB2
MIRT775969	hsa-miR-6134	Homo sapiens	SPTLC3
MIRT775970	hsa-miR-6134	Homo sapiens	TACO1
MIRT775971	hsa-miR-6134	Homo sapiens	TANK
MIRT775972	hsa-miR-6134	Homo sapiens	TAT
MIRT775973	hsa-miR-6134	Homo sapiens	TGIF1
MIRT775974	hsa-miR-6134	Homo sapiens	TGS1
MIRT775975	hsa-miR-6134	Homo sapiens	TXNL4B
MIRT775976	hsa-miR-6134	Homo sapiens	ZDHHC24
MIRT775977	hsa-miR-6134	Homo sapiens	ZNF329

MIRT788084	hsa-miR-6134	Homo sapiens	FXYD6
MIRT788085	hsa-miR-6134	Homo sapiens	MAPKAPK5
MIRT788086	hsa-miR-6134	Homo sapiens	NRIP3
MIRT788087	hsa-miR-6134	Homo sapiens	PNPLA3
MIRT788088	hsa-miR-6134	Homo sapiens	PORCN
MIRT788089	hsa-miR-6134	Homo sapiens	RAB21
MIRT788090	hsa-miR-6134	Homo sapiens	RAB9A
MIRT038997	hsa-let-7b-3p	Homo sapiens	LNPK
MIRT055419	hsa-let-7b-3p	Homo sapiens	SHOC2
MIRT057682	hsa-let-7b-3p	Homo sapiens	LCOR
MIRT061352	hsa-let-7b-3p	Homo sapiens	WEE1
MIRT062175	hsa-let-7b-3p	Homo sapiens	WNK1
MIRT071814	hsa-let-7b-3p	Homo sapiens	RNF11
MIRT091375	hsa-let-7b-3p	Homo sapiens	EIF4A2
MIRT095108	hsa-let-7b-3p	Homo sapiens	SEC24A
MIRT098815	hsa-let-7b-3p	Homo sapiens	PPIL4
MIRT109535	hsa-let-7b-3p	Homo sapiens	KLHL15
MIRT120264	hsa-let-7b-3p	Homo sapiens	GSK3B
MIRT149841	hsa-let-7b-3p	Homo sapiens	LDLR
MIRT164520	hsa-let-7b-3p	Homo sapiens	MSMO1
MIRT165880	hsa-let-7b-3p	Homo sapiens	CREBRF
MIRT169900	hsa-let-7b-3p	Homo sapiens	HBP1
MIRT182780	hsa-let-7b-3p	Homo sapiens	TOR1AIP2
MIRT193467	hsa-let-7b-3p	Homo sapiens	RORA
MIRT226422	hsa-let-7b-3p	Homo sapiens	TP53INP1
MIRT334411	hsa-let-7b-3p	Homo sapiens	CREBZF
MIRT338287	hsa-let-7b-3p	Homo sapiens	SYF2
MIRT356254	hsa-let-7b-3p	Homo sapiens	CASP3
MIRT361628	hsa-let-7b-3p	Homo sapiens	TES
MIRT406688	hsa-let-7b-3p	Homo sapiens	ZNF181
MIRT407769	hsa-let-7b-3p	Homo sapiens	MRPL35
MIRT449466	hsa-let-7b-3p	Homo sapiens	HAT1
MIRT467109	hsa-let-7b-3p	Homo sapiens	SRI
MIRT475098	hsa-let-7b-3p	Homo sapiens	IRF2BP2
MIRT481670	hsa-let-7b-3p	Homo sapiens	ARAP2
MIRT493060	hsa-let-7b-3p	Homo sapiens	MTFR1
MIRT497922	hsa-let-7b-3p	Homo sapiens	BTG1
MIRT498201	hsa-let-7b-3p	Homo sapiens	ACVR2B
MIRT503861	hsa-let-7b-3p	Homo sapiens	UBXN2B
MIRT504366	hsa-let-7b-3p	Homo sapiens	ARID1B
MIRT504990	hsa-let-7b-3p	Homo sapiens	ZNF652
MIRT505753	hsa-let-7b-3p	Homo sapiens	SENP1
MIRT518101	hsa-let-7b-3p	Homo sapiens	ADH1B
MIRT521929	hsa-let-7b-3p	Homo sapiens	PHF8

MIRT522138	hsa-let-7b-3p	Homo sapiens	NRBF2
MIRT522400	hsa-let-7b-3p	Homo sapiens	MYADM
MIRT523592	hsa-let-7b-3p	Homo sapiens	FZD5
MIRT523943	hsa-let-7b-3p	Homo sapiens	E2F8
MIRT524354	hsa-let-7b-3p	Homo sapiens	CREB1
MIRT525139	hsa-let-7b-3p	Homo sapiens	ZNF256
MIRT527069	hsa-let-7b-3p	Homo sapiens	ABCC4
MIRT527485	hsa-let-7b-3p	Homo sapiens	OCIAD1
MIRT528128	hsa-let-7b-3p	Homo sapiens	PPP1R10
MIRT530527	hsa-let-7b-3p	Homo sapiens	ALG10B
MIRT531269	hsa-let-7b-3p	Homo sapiens	PPIL3
MIRT538897	hsa-let-7b-3p	Homo sapiens	BRI3BP
MIRT541370	hsa-let-7b-3p	Homo sapiens	CDKN1B
MIRT541525	hsa-let-7b-3p	Homo sapiens	MGAT4C
MIRT543775	hsa-let-7b-3p	Homo sapiens	RBM12B
MIRT543945	hsa-let-7b-3p	Homo sapiens	NCOA7
MIRT545847	hsa-let-7b-3p	Homo sapiens	ZNF264
MIRT546063	hsa-let-7b-3p	Homo sapiens	VEZF1
MIRT546480	hsa-let-7b-3p	Homo sapiens	SLC16A14
MIRT551834	hsa-let-7b-3p	Homo sapiens	AASDHPPT
MIRT551900	hsa-let-7b-3p	Homo sapiens	ACP1
MIRT552489	hsa-let-7b-3p	Homo sapiens	ZNF136
MIRT554205	hsa-let-7b-3p	Homo sapiens	SLC35A5
MIRT554294	hsa-let-7b-3p	Homo sapiens	SIPA1L2
MIRT555764	hsa-let-7b-3p	Homo sapiens	PCTP
MIRT558314	hsa-let-7b-3p	Homo sapiens	DSG2
MIRT563146	hsa-let-7b-3p	Homo sapiens	NOLC1
MIRT566463	hsa-let-7b-3p	Homo sapiens	PGGT1B
MIRT567321	hsa-let-7b-3p	Homo sapiens	HMGB2
MIRT567891	hsa-let-7b-3p	Homo sapiens	CSTF2
MIRT570086	hsa-let-7b-3p	Homo sapiens	KANSL1L
MIRT571135	hsa-let-7b-3p	Homo sapiens	TTC33
MIRT573533	hsa-let-7b-3p	Homo sapiens	MDM2
MIRT574464	hsa-let-7b-3p	Homo sapiens	RPS16
MIRT610201	hsa-let-7b-3p	Homo sapiens	CD99
MIRT612925	hsa-let-7b-3p	Homo sapiens	GPRIN3
MIRT615026	hsa-let-7b-3p	Homo sapiens	DUSP6
MIRT617199	hsa-let-7b-3p	Homo sapiens	GREM1
MIRT628719	hsa-let-7b-3p	Homo sapiens	ZNF585A
MIRT641490	hsa-let-7b-3p	Homo sapiens	POLA2
MIRT641662	hsa-let-7b-3p	Homo sapiens	PAPOLG
MIRT642215	hsa-let-7b-3p	Homo sapiens	RUVBL2
MIRT654588	hsa-let-7b-3p	Homo sapiens	PURA
MIRT656135	hsa-let-7b-3p	Homo sapiens	MSH6

MIRT656898	hsa-let-7b-3p	Homo sapiens	USF3
MIRT660135	hsa-let-7b-3p	Homo sapiens	BRPF3
MIRT660860	hsa-let-7b-3p	Homo sapiens	AFAP1
MIRT676848	hsa-let-7b-3p	Homo sapiens	PHKA1
MIRT681478	hsa-let-7b-3p	Homo sapiens	DIP2A
MIRT685601	hsa-let-7b-3p	Homo sapiens	MYOM2
MIRT686942	hsa-let-7b-3p	Homo sapiens	SFT2D3
MIRT694301	hsa-let-7b-3p	Homo sapiens	COPB2
MIRT694406	hsa-let-7b-3p	Homo sapiens	ALDH1A3
MIRT697279	hsa-let-7b-3p	Homo sapiens	ZNF800
MIRT698413	hsa-let-7b-3p	Homo sapiens	TM4SF1
MIRT698988	hsa-let-7b-3p	Homo sapiens	SPAG9
MIRT699765	hsa-let-7b-3p	Homo sapiens	SEMA4D
MIRT699926	hsa-let-7b-3p	Homo sapiens	RUFY2
MIRT702112	hsa-let-7b-3p	Homo sapiens	MBNL1
MIRT702372	hsa-let-7b-3p	Homo sapiens	KLF10
MIRT702645	hsa-let-7b-3p	Homo sapiens	ITGA3
MIRT705712	hsa-let-7b-3p	Homo sapiens	ANAPC16
MIRT717924	hsa-let-7b-3p	Homo sapiens	ZNF546
MIRT720838	hsa-let-7b-3p	Homo sapiens	C1orf52
MIRT725032	hsa-let-7b-3p	Homo sapiens	NDUFAF7
MIRT735590	hsa-let-7b-3p	Homo sapiens	HNRNPU
MIRT735591	hsa-let-7b-3p	Homo sapiens	SGMS1
MIRT760978	hsa-let-7b-3p	Homo sapiens	LRRC75A
MIRT760979	hsa-let-7b-3p	Homo sapiens	LUZP1
MIRT760980	hsa-let-7b-3p	Homo sapiens	SMAD2
MIRT783024	hsa-let-7b-3p	Homo sapiens	CNKSR2
MIRT783025	hsa-let-7b-3p	Homo sapiens	LANCL3
MIRT053329	hsa-miR-486-3p	Homo sapiens	BCL11A
MIRT076570	hsa-miR-486-3p	Homo sapiens	PHF12
MIRT082389	hsa-miR-486-3p	Homo sapiens	HNRNPUL1
MIRT110058	hsa-miR-486-3p	Homo sapiens	OGT
MIRT292184	hsa-miR-486-3p	Homo sapiens	MKNK2
MIRT348823	hsa-miR-486-3p	Homo sapiens	U2AF2
MIRT374316	hsa-miR-486-3p	Homo sapiens	MBD6
MIRT451187	hsa-miR-486-3p	Homo sapiens	PIN1
MIRT451538	hsa-miR-486-3p	Homo sapiens	CIAPIN1
MIRT452313	hsa-miR-486-3p	Homo sapiens	EIF5AL1
MIRT452378	hsa-miR-486-3p	Homo sapiens	LY6E
MIRT452602	hsa-miR-486-3p	Homo sapiens	REPIN1
MIRT453050	hsa-miR-486-3p	Homo sapiens	TANGO2
MIRT453372	hsa-miR-486-3p	Homo sapiens	CACNA2D2
MIRT453631	hsa-miR-486-3p	Homo sapiens	SLC4A2
MIRT454471	hsa-miR-486-3p	Homo sapiens	SLC29A1

MIRT455054	hsa-miR-486-3p	Homo sapiens	MEN1
MIRT455927	hsa-miR-486-3p	Homo sapiens	HECTD3
MIRT456580	hsa-miR-486-3p	Homo sapiens	NID1
MIRT456775	hsa-miR-486-3p	Homo sapiens	MTHFSD
MIRT457515	hsa-miR-486-3p	Homo sapiens	ZMAT5
MIRT458421	hsa-miR-486-3p	Homo sapiens	KLHL38
MIRT460362	hsa-miR-486-3p	Homo sapiens	TXNDC16
MIRT461798	hsa-miR-486-3p	Homo sapiens	FXR2
MIRT462606	hsa-miR-486-3p	Homo sapiens	C20orf27
MIRT462830	hsa-miR-486-3p	Homo sapiens	BCL3
MIRT463324	hsa-miR-486-3p	Homo sapiens	ZFHX3
MIRT463433	hsa-miR-486-3p	Homo sapiens	ZC3HAV1L
MIRT464056	hsa-miR-486-3p	Homo sapiens	WAC
MIRT464314	hsa-miR-486-3p	Homo sapiens	UST
MIRT464864	hsa-miR-486-3p	Homo sapiens	UBB
MIRT464929	hsa-miR-486-3p	Homo sapiens	TXLNA
MIRT464988	hsa-miR-486-3p	Homo sapiens	TUBB2A
MIRT465578	hsa-miR-486-3p	Homo sapiens	TNRC6B
MIRT467148	hsa-miR-486-3p	Homo sapiens	SREBF2
MIRT467224	hsa-miR-486-3p	Homo sapiens	SPRED1
MIRT467842	hsa-miR-486-3p	Homo sapiens	SLC25A34
MIRT468046	hsa-miR-486-3p	Homo sapiens	SIK1
MIRT468128	hsa-miR-486-3p	Homo sapiens	SH3PXD2A
MIRT469025	hsa-miR-486-3p	Homo sapiens	RNF41
MIRT469958	hsa-miR-486-3p	Homo sapiens	PTPRF
MIRT471299	hsa-miR-486-3p	Homo sapiens	PGAM4
MIRT472392	hsa-miR-486-3p	Homo sapiens	NDRG3
MIRT472803	hsa-miR-486-3p	Homo sapiens	MTMR12
MIRT473377	hsa-miR-486-3p	Homo sapiens	MBD4
MIRT473431	hsa-miR-486-3p	Homo sapiens	MDM4
MIRT474347	hsa-miR-486-3p	Homo sapiens	KMT2D
MIRT477442	hsa-miR-486-3p	Homo sapiens	ELOVL5
MIRT478083	hsa-miR-486-3p	Homo sapiens	DLGAP4
MIRT478438	hsa-miR-486-3p	Homo sapiens	DAZAP2
MIRT478625	hsa-miR-486-3p	Homo sapiens	CTDNEP1
MIRT480084	hsa-miR-486-3p	Homo sapiens	CALR
MIRT480197	hsa-miR-486-3p	Homo sapiens	CAD
MIRT481031	hsa-miR-486-3p	Homo sapiens	BAZ2A
MIRT481187	hsa-miR-486-3p	Homo sapiens	ATXN7L3B
MIRT481224	hsa-miR-486-3p	Homo sapiens	ATXN7L3
MIRT482728	hsa-miR-486-3p	Homo sapiens	COPZ1
MIRT483271	hsa-miR-486-3p	Homo sapiens	HIVEP3
MIRT485980	hsa-miR-486-3p	Homo sapiens	PPP1R14A
MIRT486418	hsa-miR-486-3p	Homo sapiens	RXRA

MIRT487109	hsa-miR-486-3p	Homo sapiens	SCARF2
MIRT487635	hsa-miR-486-3p	Homo sapiens	BRSK2
MIRT487871	hsa-miR-486-3p	Homo sapiens	CASZ1
MIRT488348	hsa-miR-486-3p	Homo sapiens	PAX2
MIRT488785	hsa-miR-486-3p	Homo sapiens	POFUT2
MIRT488916	hsa-miR-486-3p	Homo sapiens	PTGES
MIRT489296	hsa-miR-486-3p	Homo sapiens	B4GALNT4
MIRT489352	hsa-miR-486-3p	Homo sapiens	SYNGR1
MIRT489658	hsa-miR-486-3p	Homo sapiens	SHMT1
MIRT489790	hsa-miR-486-3p	Homo sapiens	KRT80
MIRT490748	hsa-miR-486-3p	Homo sapiens	SRCIN1
MIRT490910	hsa-miR-486-3p	Homo sapiens	STRN4
MIRT491216	hsa-miR-486-3p	Homo sapiens	MRPL34
MIRT491325	hsa-miR-486-3p	Homo sapiens	GFER
MIRT491680	hsa-miR-486-3p	Homo sapiens	MNT
MIRT492986	hsa-miR-486-3p	Homo sapiens	NAV1
MIRT493957	hsa-miR-486-3p	Homo sapiens	ENG
MIRT494364	hsa-miR-486-3p	Homo sapiens	CAPN15
MIRT494529	hsa-miR-486-3p	Homo sapiens	BCL7A
MIRT494765	hsa-miR-486-3p	Homo sapiens	AP1G1
MIRT496588	hsa-miR-486-3p	Homo sapiens	TAGLN
MIRT497388	hsa-miR-486-3p	Homo sapiens	RALY
MIRT498588	hsa-miR-486-3p	Homo sapiens	KRT8
MIRT499359	hsa-miR-486-3p	Homo sapiens	RFWD3
MIRT501740	hsa-miR-486-3p	Homo sapiens	NSD1
MIRT506450	hsa-miR-486-3p	Homo sapiens	NACC2
MIRT510285	hsa-miR-486-3p	Homo sapiens	MED28
MIRT513441	hsa-miR-486-3p	Homo sapiens	EMP1
MIRT516501	hsa-miR-486-3p	Homo sapiens	SYTL3
MIRT533341	hsa-miR-486-3p	Homo sapiens	UNC119B
MIRT541102	hsa-miR-486-3p	Homo sapiens	RAF1
MIRT543667	hsa-miR-486-3p	Homo sapiens	RPL10
MIRT561448	hsa-miR-486-3p	Homo sapiens	TMCC1
MIRT569309	hsa-miR-486-3p	Homo sapiens	CC2D1B
MIRT571636	hsa-miR-486-3p	Homo sapiens	SKI
MIRT573418	hsa-miR-486-3p	Homo sapiens	RPL18A
MIRT607775	hsa-miR-486-3p	Homo sapiens	HS6ST3
MIRT608177	hsa-miR-486-3p	Homo sapiens	ERBB2
MIRT622680	hsa-miR-486-3p	Homo sapiens	PLXNA4
MIRT625928	hsa-miR-486-3p	Homo sapiens	ARHGAP18
MIRT627885	hsa-miR-486-3p	Homo sapiens	OLFML2A
MIRT637857	hsa-miR-486-3p	Homo sapiens	SC5D
MIRT638570	hsa-miR-486-3p	Homo sapiens	IER5
MIRT647387	hsa-miR-486-3p	Homo sapiens	ZNF616

MIRT650758	hsa-miR-486-3p	Homo sapiens	WNT16
MIRT658440	hsa-miR-486-3p	Homo sapiens	FAM167B
MIRT658963	hsa-miR-486-3p	Homo sapiens	DNAJC6
MIRT659667	hsa-miR-486-3p	Homo sapiens	CDC42EP4
MIRT677970	hsa-miR-486-3p	Homo sapiens	ITGB3
MIRT688992	hsa-miR-486-3p	Homo sapiens	ATP6AP1
MIRT690703	hsa-miR-486-3p	Homo sapiens	WDR73
MIRT694377	hsa-miR-486-3p	Homo sapiens	MTA1
MIRT696355	hsa-miR-486-3p	Homo sapiens	EIF2S3
MIRT696606	hsa-miR-486-3p	Homo sapiens	CRIPT
MIRT701682	hsa-miR-486-3p	Homo sapiens	MYADM
MIRT706048	hsa-miR-486-3p	Homo sapiens	PKD1
MIRT710215	hsa-miR-486-3p	Homo sapiens	JMJD4
MIRT711002	hsa-miR-486-3p	Homo sapiens	CCDC106
MIRT712419	hsa-miR-486-3p	Homo sapiens	MACROD2
MIRT712522	hsa-miR-486-3p	Homo sapiens	CYTH2
MIRT732986	hsa-miR-486-3p	Homo sapiens	ECM1
MIRT733024	hsa-miR-486-3p	Homo sapiens	MAF
MIRT734475	hsa-miR-486-3p	Homo sapiens	SYK
MIRT734477	hsa-miR-486-3p	Homo sapiens	FASN
MIRT734493	hsa-miR-486-3p	Homo sapiens	PRKCD
MIRT749087	hsa-miR-486-3p	Homo sapiens	ADIRF
MIRT749088	hsa-miR-486-3p	Homo sapiens	AGAP1
MIRT749089	hsa-miR-486-3p	Homo sapiens	AHDC1
MIRT749091	hsa-miR-486-3p	Homo sapiens	ARL4C
MIRT749092	hsa-miR-486-3p	Homo sapiens	CALM3
MIRT749093	hsa-miR-486-3p	Homo sapiens	CD276
MIRT749094	hsa-miR-486-3p	Homo sapiens	CDKN1A
MIRT749095	hsa-miR-486-3p	Homo sapiens	CEBPB
MIRT749096	hsa-miR-486-3p	Homo sapiens	CFL1
MIRT749097	hsa-miR-486-3p	Homo sapiens	CHD3
MIRT749098	hsa-miR-486-3p	Homo sapiens	COL18A1
MIRT749100	hsa-miR-486-3p	Homo sapiens	CTSA
MIRT749101	hsa-miR-486-3p	Homo sapiens	DDX39B
MIRT749103	hsa-miR-486-3p	Homo sapiens	EPHA2
MIRT749104	hsa-miR-486-3p	Homo sapiens	FZD7
MIRT749105	hsa-miR-486-3p	Homo sapiens	GPR107
MIRT749106	hsa-miR-486-3p	Homo sapiens	HCN2
MIRT749107	hsa-miR-486-3p	Homo sapiens	HMGA1
MIRT749108	hsa-miR-486-3p	Homo sapiens	IFITM3
MIRT749109	hsa-miR-486-3p	Homo sapiens	IP6K1
MIRT749110	hsa-miR-486-3p	Homo sapiens	LASP1
MIRT749111	hsa-miR-486-3p	Homo sapiens	LUZP1
MIRT749112	hsa-miR-486-3p	Homo sapiens	MAFK

MIRT749113	hsa-miR-486-3p	Homo sapiens	MAP4
MIRT749114	hsa-miR-486-3p	Homo sapiens	MYH9
MIRT749115	hsa-miR-486-3p	Homo sapiens	NACC1
MIRT749116	hsa-miR-486-3p	Homo sapiens	NCS1
MIRT749117	hsa-miR-486-3p	Homo sapiens	NFIC
MIRT749118	hsa-miR-486-3p	Homo sapiens	NPTXR
MIRT749119	hsa-miR-486-3p	Homo sapiens	NR6A1
MIRT749120	hsa-miR-486-3p	Homo sapiens	PEA15
MIRT749121	hsa-miR-486-3p	Homo sapiens	PGAM1
MIRT749122	hsa-miR-486-3p	Homo sapiens	POLL
MIRT749123	hsa-miR-486-3p	Homo sapiens	POLR2F
MIRT749124	hsa-miR-486-3p	Homo sapiens	PRAG1
MIRT749125	hsa-miR-486-3p	Homo sapiens	PTMS
MIRT749126	hsa-miR-486-3p	Homo sapiens	REXO1
MIRT749127	hsa-miR-486-3p	Homo sapiens	RNF187
MIRT749129	hsa-miR-486-3p	Homo sapiens	SBK1
MIRT749131	hsa-miR-486-3p	Homo sapiens	SLC25A22
MIRT749132	hsa-miR-486-3p	Homo sapiens	SPATA2
MIRT749133	hsa-miR-486-3p	Homo sapiens	STIP1
MIRT749134	hsa-miR-486-3p	Homo sapiens	SZRD1
MIRT749135	hsa-miR-486-3p	Homo sapiens	THY1
MIRT749136	hsa-miR-486-3p	Homo sapiens	TIMM50
MIRT749137	hsa-miR-486-3p	Homo sapiens	TUBB
MIRT749138	hsa-miR-486-3p	Homo sapiens	UBL5
MIRT749139	hsa-miR-486-3p	Homo sapiens	ZC3H7B
MIRT749140	hsa-miR-486-3p	Homo sapiens	ZNF787
MIRT772669	hsa-miR-486-3p	Homo sapiens	ATP6V1E1
MIRT772670	hsa-miR-486-3p	Homo sapiens	CRCP
MIRT772671	hsa-miR-486-3p	Homo sapiens	GDE1
MIRT772672	hsa-miR-486-3p	Homo sapiens	GPRC5C
MIRT772673	hsa-miR-486-3p	Homo sapiens	IL31RA
MIRT772674	hsa-miR-486-3p	Homo sapiens	KDM6B
MIRT772675	hsa-miR-486-3p	Homo sapiens	MFGE8
MIRT772676	hsa-miR-486-3p	Homo sapiens	NAA30
MIRT772677	hsa-miR-486-3p	Homo sapiens	OTUD4
MIRT790656	hsa-miR-486-3p	Homo sapiens	MRNIP
MIRT016142	hsa-miR-652-3p	Homo sapiens	HOXA9
MIRT039458	hsa-miR-652-3p	Homo sapiens	PRKAA1
MIRT039459	hsa-miR-652-3p	Homo sapiens	SLC12A6
MIRT039460	hsa-miR-652-3p	Homo sapiens	ZNF567
MIRT039461	hsa-miR-652-3p	Homo sapiens	NDE1
MIRT039463	hsa-miR-652-3p	Homo sapiens	TUBA1B
MIRT039464	hsa-miR-652-3p	Homo sapiens	ARCN1
MIRT039465	hsa-miR-652-3p	Homo sapiens	EEF1A1

MIRT039466	hsa-miR-652-3p	Homo sapiens	CSNK1A1
MIRT039468	hsa-miR-652-3p	Homo sapiens	SMAD2
MIRT039469	hsa-miR-652-3p	Homo sapiens	MSI2
MIRT039470	hsa-miR-652-3p	Homo sapiens	RPL26
MIRT039471	hsa-miR-652-3p	Homo sapiens	KMT2C
MIRT039472	hsa-miR-652-3p	Homo sapiens	MXRA7
MIRT039473	hsa-miR-652-3p	Homo sapiens	HMGB1
MIRT039474	hsa-miR-652-3p	Homo sapiens	MRPL36
MIRT039475	hsa-miR-652-3p	Homo sapiens	PHF12
MIRT039476	hsa-miR-652-3p	Homo sapiens	CTC1
MIRT039478	hsa-miR-652-3p	Homo sapiens	LBHD1
MIRT039479	hsa-miR-652-3p	Homo sapiens	VPS37B
MIRT039480	hsa-miR-652-3p	Homo sapiens	TCP1
MIRT039481	hsa-miR-652-3p	Homo sapiens	RAC1
MIRT039482	hsa-miR-652-3p	Homo sapiens	CACNG8
MIRT039484	hsa-miR-652-3p	Homo sapiens	MGST1
MIRT039485	hsa-miR-652-3p	Homo sapiens	AP1G1
MIRT039486	hsa-miR-652-3p	Homo sapiens	RPS29
MIRT039487	hsa-miR-652-3p	Homo sapiens	UHRF1BP1
MIRT039488	hsa-miR-652-3p	Homo sapiens	RPL18A
MIRT039489	hsa-miR-652-3p	Homo sapiens	RPL27
MIRT039491	hsa-miR-652-3p	Homo sapiens	CDKN2AIP
MIRT039492	hsa-miR-652-3p	Homo sapiens	RPL21
MIRT039493	hsa-miR-652-3p	Homo sapiens	RAP1GAP2
MIRT039496	hsa-miR-652-3p	Homo sapiens	TAZ
MIRT039497	hsa-miR-652-3p	Homo sapiens	IPO13
MIRT039499	hsa-miR-652-3p	Homo sapiens	MCTS1
MIRT039501	hsa-miR-652-3p	Homo sapiens	TMEM107
MIRT039503	hsa-miR-652-3p	Homo sapiens	YBX3
MIRT039505	hsa-miR-652-3p	Homo sapiens	ATP11C
MIRT039506	hsa-miR-652-3p	Homo sapiens	RPL29
MIRT039507	hsa-miR-652-3p	Homo sapiens	ATP11B
MIRT039508	hsa-miR-652-3p	Homo sapiens	CEBPG
MIRT039509	hsa-miR-652-3p	Homo sapiens	TMED5
MIRT039510	hsa-miR-652-3p	Homo sapiens	YIPF6
MIRT039511	hsa-miR-652-3p	Homo sapiens	ZBTB44
MIRT039512	hsa-miR-652-3p	Homo sapiens	AGO2
MIRT039513	hsa-miR-652-3p	Homo sapiens	IMMT
MIRT039514	hsa-miR-652-3p	Homo sapiens	ZBTB4
MIRT039515	hsa-miR-652-3p	Homo sapiens	SERBP1
MIRT039516	hsa-miR-652-3p	Homo sapiens	SEC13
MIRT039517	hsa-miR-652-3p	Homo sapiens	GLMP
MIRT039518	hsa-miR-652-3p	Homo sapiens	RPS6
MIRT039519	hsa-miR-652-3p	Homo sapiens	RPL32

MIRT039520	hsa-miR-652-3p	Homo sapiens	EML4
MIRT039521	hsa-miR-652-3p	Homo sapiens	PCSK7
MIRT039522	hsa-miR-652-3p	Homo sapiens	GRPEL1
MIRT039523	hsa-miR-652-3p	Homo sapiens	HACD1
MIRT039524	hsa-miR-652-3p	Homo sapiens	CD46
MIRT039525	hsa-miR-652-3p	Homo sapiens	GTF3C5
MIRT039527	hsa-miR-652-3p	Homo sapiens	RNF152
MIRT039528	hsa-miR-652-3p	Homo sapiens	DDX39A
MIRT039529	hsa-miR-652-3p	Homo sapiens	MRAS
MIRT039530	hsa-miR-652-3p	Homo sapiens	CSNK2A1
MIRT039531	hsa-miR-652-3p	Homo sapiens	G6PC3
MIRT039532	hsa-miR-652-3p	Homo sapiens	CNN3
MIRT039533	hsa-miR-652-3p	Homo sapiens	AGO1
MIRT039534	hsa-miR-652-3p	Homo sapiens	NUP98
MIRT039535	hsa-miR-652-3p	Homo sapiens	RPL35A
MIRT039536	hsa-miR-652-3p	Homo sapiens	SUPT6H
MIRT039538	hsa-miR-652-3p	Homo sapiens	SNX5
MIRT039539	hsa-miR-652-3p	Homo sapiens	CDC42EP1
MIRT039540	hsa-miR-652-3p	Homo sapiens	BTF3
MIRT039541	hsa-miR-652-3p	Homo sapiens	IRS4
MIRT039542	hsa-miR-652-3p	Homo sapiens	SMAP2
MIRT039543	hsa-miR-652-3p	Homo sapiens	POM121
MIRT039544	hsa-miR-652-3p	Homo sapiens	CNOT3
MIRT039546	hsa-miR-652-3p	Homo sapiens	RPL4
MIRT039549	hsa-miR-652-3p	Homo sapiens	NFAT5
MIRT039550	hsa-miR-652-3p	Homo sapiens	DGCR8
MIRT039551	hsa-miR-652-3p	Homo sapiens	SAT1
MIRT039552	hsa-miR-652-3p	Homo sapiens	EIF4ENIF1
MIRT039553	hsa-miR-652-3p	Homo sapiens	MORF4L2
MIRT039554	hsa-miR-652-3p	Homo sapiens	QKI
MIRT039557	hsa-miR-652-3p	Homo sapiens	POLR2A
MIRT039559	hsa-miR-652-3p	Homo sapiens	SRPK1
MIRT039560	hsa-miR-652-3p	Homo sapiens	ACTG1
MIRT039561	hsa-miR-652-3p	Homo sapiens	KIF1A
MIRT039562	hsa-miR-652-3p	Homo sapiens	NXN
MIRT039563	hsa-miR-652-3p	Homo sapiens	USP10
MIRT039564	hsa-miR-652-3p	Homo sapiens	AGAP3
MIRT039565	hsa-miR-652-3p	Homo sapiens	ELOVL1
MIRT039566	hsa-miR-652-3p	Homo sapiens	HSPA1B
MIRT039567	hsa-miR-652-3p	Homo sapiens	GRK6
MIRT039568	hsa-miR-652-3p	Homo sapiens	ACTN4
MIRT039569	hsa-miR-652-3p	Homo sapiens	RPS16
MIRT039570	hsa-miR-652-3p	Homo sapiens	ISOC1
MIRT039571	hsa-miR-652-3p	Homo sapiens	CBS

MIRT115494	hsa-miR-652-3p	Homo sapiens	TNRC6A
MIRT510693	hsa-miR-652-3p	Homo sapiens	SRM
MIRT538410	hsa-miR-652-3p	Homo sapiens	COX20
MIRT562196	hsa-miR-652-3p	Homo sapiens	HNRNPAB
MIRT629414	hsa-miR-652-3p	Homo sapiens	ADM2
MIRT629877	hsa-miR-652-3p	Homo sapiens	NOM1
MIRT630160	hsa-miR-652-3p	Homo sapiens	ZBTB8A
MIRT631069	hsa-miR-652-3p	Homo sapiens	KDM2B
MIRT632050	hsa-miR-652-3p	Homo sapiens	ATF7IP
MIRT632253	hsa-miR-652-3p	Homo sapiens	VPS41
MIRT632707	hsa-miR-652-3p	Homo sapiens	MTA3
MIRT644918	hsa-miR-652-3p	Homo sapiens	SERF1B
MIRT654948	hsa-miR-652-3p	Homo sapiens	PNPT1
MIRT660830	hsa-miR-652-3p	Homo sapiens	AGO3
MIRT663529	hsa-miR-652-3p	Homo sapiens	MASTL
MIRT665166	hsa-miR-652-3p	Homo sapiens	SF3A1
MIRT666260	hsa-miR-652-3p	Homo sapiens	SLC31A1
MIRT667232	hsa-miR-652-3p	Homo sapiens	NFE2L1
MIRT674032	hsa-miR-652-3p	Homo sapiens	ANKRD9
MIRT674224	hsa-miR-652-3p	Homo sapiens	FAM120AOS
MIRT675272	hsa-miR-652-3p	Homo sapiens	ZNF431
MIRT688819	hsa-miR-652-3p	Homo sapiens	CAPZB
MIRT689239	hsa-miR-652-3p	Homo sapiens	RPS19
MIRT694025	hsa-miR-652-3p	Homo sapiens	PPIL4
MIRT694351	hsa-miR-652-3p	Homo sapiens	CHST6
MIRT698782	hsa-miR-652-3p	Homo sapiens	STK4
MIRT703337	hsa-miR-652-3p	Homo sapiens	GDPD5
MIRT732957	hsa-miR-652-3p	Homo sapiens	LLGL1
MIRT734659	hsa-miR-652-3p	Homo sapiens	ZEB1
MIRT753408	hsa-miR-652-3p	Homo sapiens	BSG
MIRT753410	hsa-miR-652-3p	Homo sapiens	CYTH2
MIRT753412	hsa-miR-652-3p	Homo sapiens	GEN1
MIRT753413	hsa-miR-652-3p	Homo sapiens	UBE2I
MIRT777267	hsa-miR-652-3p	Homo sapiens	GAN
MIRT777268	hsa-miR-652-3p	Homo sapiens	GGCX
MIRT777269	hsa-miR-652-3p	Homo sapiens	GPR161
MIRT777270	hsa-miR-652-3p	Homo sapiens	LAT2
MIRT777271	hsa-miR-652-3p	Homo sapiens	LRRC1
MIRT777272	hsa-miR-652-3p	Homo sapiens	PNPLA4
MIRT777273	hsa-miR-652-3p	Homo sapiens	PRPF38A
MIRT777274	hsa-miR-652-3p	Homo sapiens	QSOX1
MIRT777275	hsa-miR-652-3p	Homo sapiens	TMEM250
MIRT777276	hsa-miR-652-3p	Homo sapiens	TMEM33
MIRT097121	hsa-miR-640	Homo sapiens	TNPO1

MIRT115090	hsa-miR-640	Homo sapiens	ARIH1
MIRT204603	hsa-miR-640	Homo sapiens	HSPE1-MOB4
MIRT204634	hsa-miR-640	Homo sapiens	MOB4
MIRT344451	hsa-miR-640	Homo sapiens	MTRNR2L1
MIRT405772	hsa-miR-640	Homo sapiens	EIF5
MIRT445876	hsa-miR-640	Homo sapiens	SENP6
MIRT504691	hsa-miR-640	Homo sapiens	SLCO2B1
MIRT512386	hsa-miR-640	Homo sapiens	MTRNR2L3
MIRT513003	hsa-miR-640	Homo sapiens	MAN1A2
MIRT513084	hsa-miR-640	Homo sapiens	USP9X
MIRT519122	hsa-miR-640	Homo sapiens	ALDH2
MIRT565356	hsa-miR-640	Homo sapiens	TMCC1
MIRT613780	hsa-miR-640	Homo sapiens	RPS6
MIRT613934	hsa-miR-640	Homo sapiens	POLR3A
MIRT614348	hsa-miR-640	Homo sapiens	BORCS5
MIRT614534	hsa-miR-640	Homo sapiens	NOA1
MIRT618247	hsa-miR-640	Homo sapiens	MANEAL
MIRT619051	hsa-miR-640	Homo sapiens	TTC4
MIRT619442	hsa-miR-640	Homo sapiens	ZNF517
MIRT619723	hsa-miR-640	Homo sapiens	FPR2
MIRT620179	hsa-miR-640	Homo sapiens	TRIM72
MIRT621263	hsa-miR-640	Homo sapiens	RTN2
MIRT621463	hsa-miR-640	Homo sapiens	APOH
MIRT621571	hsa-miR-640	Homo sapiens	ZBTB43
MIRT621683	hsa-miR-640	Homo sapiens	TSPYL1
MIRT622866	hsa-miR-640	Homo sapiens	PDE7A
MIRT624669	hsa-miR-640	Homo sapiens	ARHGEF39
MIRT625607	hsa-miR-640	Homo sapiens	ZNF84
MIRT628380	hsa-miR-640	Homo sapiens	CACNB2
MIRT628553	hsa-miR-640	Homo sapiens	MELK
MIRT628747	hsa-miR-640	Homo sapiens	TRPV2
MIRT628776	hsa-miR-640	Homo sapiens	TMEM154
MIRT628918	hsa-miR-640	Homo sapiens	ZNF430
MIRT629174	hsa-miR-640	Homo sapiens	ALDOA
MIRT629209	hsa-miR-640	Homo sapiens	C12orf66
MIRT629267	hsa-miR-640	Homo sapiens	SLC5A8
MIRT629498	hsa-miR-640	Homo sapiens	AS3MT
MIRT629665	hsa-miR-640	Homo sapiens	USP1
MIRT629760	hsa-miR-640	Homo sapiens	STK25
MIRT630899	hsa-miR-640	Homo sapiens	GATAD1
MIRT631105	hsa-miR-640	Homo sapiens	SLC15A2
MIRT631112	hsa-miR-640	Homo sapiens	ATCAY
MIRT631580	hsa-miR-640	Homo sapiens	ITGAL
MIRT631836	hsa-miR-640	Homo sapiens	CMBL

MIRT632342	hsa-miR-640	Homo sapiens	SWSAP1
MIRT633214	hsa-miR-640	Homo sapiens	ZNF584
MIRT633223	hsa-miR-640	Homo sapiens	ZNF43
MIRT633511	hsa-miR-640	Homo sapiens	LRRC27
MIRT633615	hsa-miR-640	Homo sapiens	CWF19L1
MIRT633652	hsa-miR-640	Homo sapiens	SLC28A1
MIRT633677	hsa-miR-640	Homo sapiens	ZNF576
MIRT634196	hsa-miR-640	Homo sapiens	TMOD2
MIRT634388	hsa-miR-640	Homo sapiens	PLSCR1
MIRT635817	hsa-miR-640	Homo sapiens	OPA3
MIRT635962	hsa-miR-640	Homo sapiens	TTC31
MIRT636114	hsa-miR-640	Homo sapiens	YPEL1
MIRT636164	hsa-miR-640	Homo sapiens	TIMM8A
MIRT636768	hsa-miR-640	Homo sapiens	CLUAP1
MIRT637092	hsa-miR-640	Homo sapiens	BCLAF3
MIRT637540	hsa-miR-640	Homo sapiens	CHST6
MIRT637628	hsa-miR-640	Homo sapiens	ZNF431
MIRT637826	hsa-miR-640	Homo sapiens	CACNG8
MIRT637945	hsa-miR-640	Homo sapiens	IVD
MIRT637968	hsa-miR-640	Homo sapiens	IRF1
MIRT638321	hsa-miR-640	Homo sapiens	RNF11
MIRT638386	hsa-miR-640	Homo sapiens	RAB11FIP1
MIRT639244	hsa-miR-640	Homo sapiens	CRK
MIRT642793	hsa-miR-640	Homo sapiens	SLC1A5
MIRT643847	hsa-miR-640	Homo sapiens	LACTB
MIRT645148	hsa-miR-640	Homo sapiens	DIS3
MIRT646671	hsa-miR-640	Homo sapiens	CCDC69
MIRT647780	hsa-miR-640	Homo sapiens	ASB8
MIRT648554	hsa-miR-640	Homo sapiens	WDR92
MIRT648990	hsa-miR-640	Homo sapiens	MRPL49
MIRT649099	hsa-miR-640	Homo sapiens	KCNMB1
MIRT650141	hsa-miR-640	Homo sapiens	ZNF426
MIRT650791	hsa-miR-640	Homo sapiens	GSR
MIRT654967	hsa-miR-640	Homo sapiens	PLEKHA2
MIRT655099	hsa-miR-640	Homo sapiens	PHLDA3
MIRT655516	hsa-miR-640	Homo sapiens	PAG1
MIRT656289	hsa-miR-640	Homo sapiens	METTL14
MIRT656497	hsa-miR-640	Homo sapiens	MAP3K9
MIRT657074	hsa-miR-640	Homo sapiens	JPH2
MIRT657314	hsa-miR-640	Homo sapiens	HOOK3
MIRT657419	hsa-miR-640	Homo sapiens	HIF1AN
MIRT659032	hsa-miR-640	Homo sapiens	DHTKD1
MIRT659535	hsa-miR-640	Homo sapiens	CHCHD5
MIRT661532	hsa-miR-640	Homo sapiens	NWD1

MIRT662029	hsa-miR-640	Homo sapiens	FUT2
MIRT663206	hsa-miR-640	Homo sapiens	DARS2
MIRT663357	hsa-miR-640	Homo sapiens	ORAI2
MIRT663557	hsa-miR-640	Homo sapiens	CCR6
MIRT663650	hsa-miR-640	Homo sapiens	POLM
MIRT663694	hsa-miR-640	Homo sapiens	ABHD17B
MIRT664370	hsa-miR-640	Homo sapiens	CYB5A
MIRT664783	hsa-miR-640	Homo sapiens	LIAS
MIRT665561	hsa-miR-640	Homo sapiens	TXNL1
MIRT665950	hsa-miR-640	Homo sapiens	TAOK1
MIRT667338	hsa-miR-640	Homo sapiens	MSANTD3
MIRT667804	hsa-miR-640	Homo sapiens	ITIH5
MIRT668804	hsa-miR-640	Homo sapiens	CYP20A1
MIRT669470	hsa-miR-640	Homo sapiens	ARPC2
MIRT669599	hsa-miR-640	Homo sapiens	AGO3
MIRT669806	hsa-miR-640	Homo sapiens	STOML1
MIRT669944	hsa-miR-640	Homo sapiens	FBXL2
MIRT670292	hsa-miR-640	Homo sapiens	RBBP4
MIRT670381	hsa-miR-640	Homo sapiens	EMP2
MIRT670481	hsa-miR-640	Homo sapiens	DCUN1D2
MIRT670531	hsa-miR-640	Homo sapiens	KIF1C
MIRT670565	hsa-miR-640	Homo sapiens	GLTP
MIRT670603	hsa-miR-640	Homo sapiens	NPHP1
MIRT670880	hsa-miR-640	Homo sapiens	CYTIP
MIRT670931	hsa-miR-640	Homo sapiens	LIPG
MIRT671794	hsa-miR-640	Homo sapiens	FLVCR1
MIRT671897	hsa-miR-640	Homo sapiens	GBP4
MIRT672000	hsa-miR-640	Homo sapiens	SLC35F6
MIRT672853	hsa-miR-640	Homo sapiens	RTL10
MIRT673537	hsa-miR-640	Homo sapiens	DEGS1
MIRT674285	hsa-miR-640	Homo sapiens	ZNF724
MIRT674494	hsa-miR-640	Homo sapiens	TIRAP
MIRT675460	hsa-miR-640	Homo sapiens	NUBPL
MIRT675569	hsa-miR-640	Homo sapiens	TRIP11
MIRT676068	hsa-miR-640	Homo sapiens	TIMM50
MIRT676377	hsa-miR-640	Homo sapiens	SEC24D
MIRT676758	hsa-miR-640	Homo sapiens	SNX2
MIRT676773	hsa-miR-640	Homo sapiens	NPHS1
MIRT676874	hsa-miR-640	Homo sapiens	ENSA
MIRT676944	hsa-miR-640	Homo sapiens	S1PR3
MIRT676967	hsa-miR-640	Homo sapiens	RNF19B
MIRT676972	hsa-miR-640	Homo sapiens	ZNF708
MIRT677042	hsa-miR-640	Homo sapiens	ZNF34
MIRT677070	hsa-miR-640	Homo sapiens	VMAC

MIRT677093	hsa-miR-640	Homo sapiens	MFSD11
MIRT677134	hsa-miR-640	Homo sapiens	P2RX7
MIRT677179	hsa-miR-640	Homo sapiens	ZNF786
MIRT677228	hsa-miR-640	Homo sapiens	C15orf40
MIRT677320	hsa-miR-640	Homo sapiens	PIGO
MIRT677455	hsa-miR-640	Homo sapiens	PDLIM3
MIRT677809	hsa-miR-640	Homo sapiens	MRPS10
MIRT677937	hsa-miR-640	Homo sapiens	ZNF519
MIRT678063	hsa-miR-640	Homo sapiens	UBN2
MIRT678072	hsa-miR-640	Homo sapiens	EIF2A
MIRT678252	hsa-miR-640	Homo sapiens	FXN
MIRT678315	hsa-miR-640	Homo sapiens	FBLIM1
MIRT678367	hsa-miR-640	Homo sapiens	XIAP
MIRT678372	hsa-miR-640	Homo sapiens	RNF115
MIRT678410	hsa-miR-640	Homo sapiens	ANKRD36
MIRT678518	hsa-miR-640	Homo sapiens	ZNF347
MIRT678566	hsa-miR-640	Homo sapiens	CDK4
MIRT678580	hsa-miR-640	Homo sapiens	PPP1R3B
MIRT678820	hsa-miR-640	Homo sapiens	PDE6A
MIRT678917	hsa-miR-640	Homo sapiens	XPOT
MIRT679217	hsa-miR-640	Homo sapiens	MAN2A2
MIRT679437	hsa-miR-640	Homo sapiens	TIMM29
MIRT679595	hsa-miR-640	Homo sapiens	HILPDA
MIRT679755	hsa-miR-640	Homo sapiens	TLR6
MIRT679800	hsa-miR-640	Homo sapiens	APOBEC3A
MIRT680057	hsa-miR-640	Homo sapiens	CD96
MIRT680116	hsa-miR-640	Homo sapiens	CCDC30
MIRT680181	hsa-miR-640	Homo sapiens	ZNF554
MIRT680439	hsa-miR-640	Homo sapiens	WDR12
MIRT680789	hsa-miR-640	Homo sapiens	ZNF578
MIRT692464	hsa-miR-640	Homo sapiens	APEX2
MIRT702952	hsa-miR-640	Homo sapiens	HIP1
MIRT706016	hsa-miR-640	Homo sapiens	ZSCAN2
MIRT706032	hsa-miR-640	Homo sapiens	F2R
MIRT706135	hsa-miR-640	Homo sapiens	MTRNR2L10
MIRT706394	hsa-miR-640	Homo sapiens	HAS2
MIRT713293	hsa-miR-640	Homo sapiens	DCP2
MIRT716583	hsa-miR-640	Homo sapiens	BRAP
MIRT720433	hsa-miR-640	Homo sapiens	C19orf47
MIRT752713	hsa-miR-640	Homo sapiens	ABHD15
MIRT752715	hsa-miR-640	Homo sapiens	POTEM
MIRT752716	hsa-miR-640	Homo sapiens	SOCS7
MIRT776153	hsa-miR-640	Homo sapiens	COX8A
MIRT776154	hsa-miR-640	Homo sapiens	EFCAB11

MIRT788168	hsa-miR-640	Homo sapiens	FAM241A
MIRT788169	hsa-miR-640	Homo sapiens	LRRC58
MIRT788170	hsa-miR-640	Homo sapiens	MPPE1
MIRT788171	hsa-miR-640	Homo sapiens	ZNF257
MIRT000116	hsa-miR-24-3p	Homo sapiens	FEN1
MIRT000117	hsa-miR-24-3p	Homo sapiens	CDK4
MIRT000119	hsa-miR-24-3p	Homo sapiens	CCNA2
MIRT000120	hsa-miR-24-3p	Homo sapiens	AURKB
MIRT000121	hsa-miR-24-3p	Homo sapiens	MYC
MIRT000122	hsa-miR-24-3p	Homo sapiens	E2F2
MIRT001773	hsa-miR-24-3p	Homo sapiens	NOTCH1
MIRT002018	hsa-miR-24-3p	Homo sapiens	DHFR
MIRT002950	hsa-miR-24-3p	Homo sapiens	MAPK14
MIRT003354	hsa-miR-24-3p	Homo sapiens	TRIB3
MIRT003355	hsa-miR-24-3p	Homo sapiens	HNF4A
MIRT003830	hsa-miR-24-3p	Homo sapiens	ACVR1B
MIRT003889	hsa-miR-24-3p	Homo sapiens	MLEC
MIRT004362	hsa-miR-24-3p	Homo sapiens	CDKN2A
MIRT004836	hsa-miR-24-3p	Homo sapiens	BRCA1
MIRT004837	hsa-miR-24-3p	Homo sapiens	POLD1
MIRT005063	hsa-miR-24-3p	Homo sapiens	CDKN1B
MIRT005397	hsa-miR-24-3p	Homo sapiens	KHSRP
MIRT005398	hsa-miR-24-3p	Homo sapiens	NFAT5
MIRT005766	hsa-miR-24-3p	Homo sapiens	DND1
MIRT005918	hsa-miR-24-3p	Homo sapiens	TGFB1
MIRT005919	hsa-miR-24-3p	Homo sapiens	FURIN
MIRT006507	hsa-miR-24-3p	Homo sapiens	FAF1
MIRT007012	hsa-miR-24-3p	Homo sapiens	ZNF217
MIRT007215	hsa-miR-24-3p	Homo sapiens	ST7L
MIRT030361	hsa-miR-24-3p	Homo sapiens	VRK1
MIRT030362	hsa-miR-24-3p	Homo sapiens	NOP56
MIRT030363	hsa-miR-24-3p	Homo sapiens	TBPL1
MIRT030364	hsa-miR-24-3p	Homo sapiens	TNPO3
MIRT030365	hsa-miR-24-3p	Homo sapiens	SNRPB2
MIRT030366	hsa-miR-24-3p	Homo sapiens	DDHD2
MIRT030367	hsa-miR-24-3p	Homo sapiens	THOP1
MIRT030369	hsa-miR-24-3p	Homo sapiens	UBE2C
MIRT030370	hsa-miR-24-3p	Homo sapiens	GNPAT
MIRT030371	hsa-miR-24-3p	Homo sapiens	METAP2
MIRT030372	hsa-miR-24-3p	Homo sapiens	PUM3
MIRT030373	hsa-miR-24-3p	Homo sapiens	UGDH
MIRT030374	hsa-miR-24-3p	Homo sapiens	URM1
MIRT030375	hsa-miR-24-3p	Homo sapiens	GTF2E1
MIRT030376	hsa-miR-24-3p	Homo sapiens	MED22

MIRT030377	hsa-miR-24-3p	Homo sapiens	USP10
MIRT030378	hsa-miR-24-3p	Homo sapiens	FKBP1B
MIRT030379	hsa-miR-24-3p	Homo sapiens	SCML1
MIRT030380	hsa-miR-24-3p	Homo sapiens	CNDP2
MIRT030381	hsa-miR-24-3p	Homo sapiens	MCM10
MIRT030382	hsa-miR-24-3p	Homo sapiens	TOP1
MIRT030384	hsa-miR-24-3p	Homo sapiens	TOMM22
MIRT030385	hsa-miR-24-3p	Homo sapiens	HBQ1
MIRT030386	hsa-miR-24-3p	Homo sapiens	PRIM1
MIRT030387	hsa-miR-24-3p	Homo sapiens	PSMD1
MIRT030388	hsa-miR-24-3p	Homo sapiens	AUNIP
MIRT030389	hsa-miR-24-3p	Homo sapiens	NARF
MIRT030390	hsa-miR-24-3p	Homo sapiens	MALSU1
MIRT030391	hsa-miR-24-3p	Homo sapiens	TCEA3
MIRT030392	hsa-miR-24-3p	Homo sapiens	ADRM1
MIRT030393	hsa-miR-24-3p	Homo sapiens	AGFG1
MIRT030394	hsa-miR-24-3p	Homo sapiens	ACD
MIRT030395	hsa-miR-24-3p	Homo sapiens	RCE1
MIRT030396	hsa-miR-24-3p	Homo sapiens	SUMO3
MIRT030397	hsa-miR-24-3p	Homo sapiens	CYP20A1
MIRT030399	hsa-miR-24-3p	Homo sapiens	MIS18A
MIRT030400	hsa-miR-24-3p	Homo sapiens	GLYR1
MIRT030401	hsa-miR-24-3p	Homo sapiens	NAE1
MIRT030402	hsa-miR-24-3p	Homo sapiens	ACTL6A
MIRT030403	hsa-miR-24-3p	Homo sapiens	NCBP2
MIRT030405	hsa-miR-24-3p	Homo sapiens	JADE3
MIRT030407	hsa-miR-24-3p	Homo sapiens	SLC7A2
MIRT030408	hsa-miR-24-3p	Homo sapiens	EXOSC8
MIRT030409	hsa-miR-24-3p	Homo sapiens	TDRP
MIRT030410	hsa-miR-24-3p	Homo sapiens	R3HDM4
MIRT030411	hsa-miR-24-3p	Homo sapiens	DCAF4
MIRT030412	hsa-miR-24-3p	Homo sapiens	TAF15
MIRT030413	hsa-miR-24-3p	Homo sapiens	VPS25
MIRT030415	hsa-miR-24-3p	Homo sapiens	NASP
MIRT030416	hsa-miR-24-3p	Homo sapiens	STRADB
MIRT030417	hsa-miR-24-3p	Homo sapiens	MTF2
MIRT030418	hsa-miR-24-3p	Homo sapiens	PHOSPHO2
MIRT030419	hsa-miR-24-3p	Homo sapiens	ATAD3A
MIRT030420	hsa-miR-24-3p	Homo sapiens	CCDC59
MIRT030421	hsa-miR-24-3p	Homo sapiens	VPS35
MIRT030422	hsa-miR-24-3p	Homo sapiens	PAF1
MIRT030423	hsa-miR-24-3p	Homo sapiens	CIRBP
MIRT030424	hsa-miR-24-3p	Homo sapiens	S100P
MIRT030425	hsa-miR-24-3p	Homo sapiens	ARHGEF7

MIRT030426	hsa-miR-24-3p	Homo sapiens	PROSER1
MIRT030427	hsa-miR-24-3p	Homo sapiens	PDLIM7
MIRT030428	hsa-miR-24-3p	Homo sapiens	KIAA0100
MIRT030429	hsa-miR-24-3p	Homo sapiens	TOMM34
MIRT030430	hsa-miR-24-3p	Homo sapiens	CTCF
MIRT030431	hsa-miR-24-3p	Homo sapiens	EIF4G3
MIRT030432	hsa-miR-24-3p	Homo sapiens	SLC52A2
MIRT030433	hsa-miR-24-3p	Homo sapiens	KHNYN
MIRT030434	hsa-miR-24-3p	Homo sapiens	ADD1
MIRT030435	hsa-miR-24-3p	Homo sapiens	ZBED1
MIRT030436	hsa-miR-24-3p	Homo sapiens	KLHL23
MIRT030438	hsa-miR-24-3p	Homo sapiens	LSM12
MIRT030439	hsa-miR-24-3p	Homo sapiens	PDPK1
MIRT030440	hsa-miR-24-3p	Homo sapiens	CARD10
MIRT030441	hsa-miR-24-3p	Homo sapiens	PPM1F
MIRT030442	hsa-miR-24-3p	Homo sapiens	NRIP1
MIRT030443	hsa-miR-24-3p	Homo sapiens	ARHGEF18
MIRT030444	hsa-miR-24-3p	Homo sapiens	FNTB
MIRT030445	hsa-miR-24-3p	Homo sapiens	PAK4
MIRT030446	hsa-miR-24-3p	Homo sapiens	KPNA6
MIRT030447	hsa-miR-24-3p	Homo sapiens	BCL2L2
MIRT030448	hsa-miR-24-3p	Homo sapiens	PSTPIP2
MIRT030449	hsa-miR-24-3p	Homo sapiens	ACACA
MIRT030450	hsa-miR-24-3p	Homo sapiens	MATR3
MIRT030451	hsa-miR-24-3p	Homo sapiens	HACD3
MIRT030452	hsa-miR-24-3p	Homo sapiens	TSPAN14
MIRT030453	hsa-miR-24-3p	Homo sapiens	RAP2C
MIRT030454	hsa-miR-24-3p	Homo sapiens	MIDN
MIRT030456	hsa-miR-24-3p	Homo sapiens	LAPTM4B
MIRT030457	hsa-miR-24-3p	Homo sapiens	PPP3R1
MIRT030458	hsa-miR-24-3p	Homo sapiens	ZNF813
MIRT030459	hsa-miR-24-3p	Homo sapiens	MAGI1
MIRT030460	hsa-miR-24-3p	Homo sapiens	ZCCHC14
MIRT030461	hsa-miR-24-3p	Homo sapiens	PTGFRN
MIRT030462	hsa-miR-24-3p	Homo sapiens	SCML2
MIRT030463	hsa-miR-24-3p	Homo sapiens	DNAJB12
MIRT030464	hsa-miR-24-3p	Homo sapiens	NUP54
MIRT030465	hsa-miR-24-3p	Homo sapiens	SESN1
MIRT030466	hsa-miR-24-3p	Homo sapiens	SLC35B2
MIRT030467	hsa-miR-24-3p	Homo sapiens	AGPAT3
MIRT030468	hsa-miR-24-3p	Homo sapiens	UBD
MIRT030469	hsa-miR-24-3p	Homo sapiens	RRM2
MIRT030470	hsa-miR-24-3p	Homo sapiens	BCL2L12
MIRT030471	hsa-miR-24-3p	Homo sapiens	MBD6

MIRT030472	hsa-miR-24-3p	Homo sapiens	OXSR1
MIRT030473	hsa-miR-24-3p	Homo sapiens	PER2
MIRT030474	hsa-miR-24-3p	Homo sapiens	UNG
MIRT030475	hsa-miR-24-3p	Homo sapiens	RRP12
MIRT030476	hsa-miR-24-3p	Homo sapiens	CWC27
MIRT030477	hsa-miR-24-3p	Homo sapiens	SRRT
MIRT030480	hsa-miR-24-3p	Homo sapiens	ALG5
MIRT030481	hsa-miR-24-3p	Homo sapiens	SLC2A3
MIRT030482	hsa-miR-24-3p	Homo sapiens	MRPS24
MIRT030483	hsa-miR-24-3p	Homo sapiens	EIF2S3
MIRT030484	hsa-miR-24-3p	Homo sapiens	GTF3C2
MIRT030485	hsa-miR-24-3p	Homo sapiens	FAH
MIRT030486	hsa-miR-24-3p	Homo sapiens	ARL1
MIRT030487	hsa-miR-24-3p	Homo sapiens	MED16
MIRT030488	hsa-miR-24-3p	Homo sapiens	MED24
MIRT030489	hsa-miR-24-3p	Homo sapiens	CCAR1
MIRT030490	hsa-miR-24-3p	Homo sapiens	DUS1L
MIRT030491	hsa-miR-24-3p	Homo sapiens	BEX1
MIRT030492	hsa-miR-24-3p	Homo sapiens	MRPL40
MIRT030493	hsa-miR-24-3p	Homo sapiens	EIF4H
MIRT030494	hsa-miR-24-3p	Homo sapiens	DCP2
MIRT030495	hsa-miR-24-3p	Homo sapiens	ABCE1
MIRT030496	hsa-miR-24-3p	Homo sapiens	UBE3A
MIRT030497	hsa-miR-24-3p	Homo sapiens	TYW3
MIRT030498	hsa-miR-24-3p	Homo sapiens	UQCC1
MIRT030499	hsa-miR-24-3p	Homo sapiens	NKD1
MIRT030500	hsa-miR-24-3p	Homo sapiens	NFKBIA
MIRT030501	hsa-miR-24-3p	Homo sapiens	AK3
MIRT030503	hsa-miR-24-3p	Homo sapiens	CDK1
MIRT030504	hsa-miR-24-3p	Homo sapiens	HSF2
MIRT030505	hsa-miR-24-3p	Homo sapiens	CDCA7
MIRT030507	hsa-miR-24-3p	Homo sapiens	TNIP2
MIRT030508	hsa-miR-24-3p	Homo sapiens	AKAP7
MIRT030509	hsa-miR-24-3p	Homo sapiens	TUBGCP2
MIRT030510	hsa-miR-24-3p	Homo sapiens	POLR2D
MIRT030511	hsa-miR-24-3p	Homo sapiens	FBXO34
MIRT030512	hsa-miR-24-3p	Homo sapiens	STK35
MIRT030513	hsa-miR-24-3p	Homo sapiens	CTDSP2
MIRT030514	hsa-miR-24-3p	Homo sapiens	C8orf33
MIRT030516	hsa-miR-24-3p	Homo sapiens	IQCB1
MIRT030517	hsa-miR-24-3p	Homo sapiens	RHOT2
MIRT030518	hsa-miR-24-3p	Homo sapiens	ALDH5A1
MIRT030519	hsa-miR-24-3p	Homo sapiens	VHL
MIRT030520	hsa-miR-24-3p	Homo sapiens	SLC11A2

MIRT030521	hsa-miR-24-3p	Homo sapiens	NIPSNAP2
MIRT030522	hsa-miR-24-3p	Homo sapiens	SLC5A6
MIRT030523	hsa-miR-24-3p	Homo sapiens	NEK6
MIRT030525	hsa-miR-24-3p	Homo sapiens	GGA2
MIRT030526	hsa-miR-24-3p	Homo sapiens	ADPGK
MIRT030528	hsa-miR-24-3p	Homo sapiens	FZD4
MIRT030529	hsa-miR-24-3p	Homo sapiens	AAMP
MIRT030530	hsa-miR-24-3p	Homo sapiens	PLIN3
MIRT030531	hsa-miR-24-3p	Homo sapiens	LLGL1
MIRT030532	hsa-miR-24-3p	Homo sapiens	KLHDC3
MIRT030533	hsa-miR-24-3p	Homo sapiens	C15orf39
MIRT030534	hsa-miR-24-3p	Homo sapiens	MARCKSL1
MIRT030536	hsa-miR-24-3p	Homo sapiens	ADD3
MIRT030537	hsa-miR-24-3p	Homo sapiens	SNTB1
MIRT030538	hsa-miR-24-3p	Homo sapiens	CMTM4
MIRT030539	hsa-miR-24-3p	Homo sapiens	TMTC4
MIRT030540	hsa-miR-24-3p	Homo sapiens	GLUL
MIRT030541	hsa-miR-24-3p	Homo sapiens	TMEM209
MIRT030542	hsa-miR-24-3p	Homo sapiens	LBR
MIRT030543	hsa-miR-24-3p	Homo sapiens	LIMD1
MIRT030544	hsa-miR-24-3p	Homo sapiens	SPIN4
MIRT030545	hsa-miR-24-3p	Homo sapiens	ZNF264
MIRT030546	hsa-miR-24-3p	Homo sapiens	VGLL3
MIRT030547	hsa-miR-24-3p	Homo sapiens	BCL2L11
MIRT030548	hsa-miR-24-3p	Homo sapiens	DEDD
MIRT030549	hsa-miR-24-3p	Homo sapiens	CD34
MIRT030550	hsa-miR-24-3p	Homo sapiens	TMED7
MIRT030551	hsa-miR-24-3p	Homo sapiens	E2F3
MIRT030552	hsa-miR-24-3p	Homo sapiens	ZNF317
MIRT030553	hsa-miR-24-3p	Homo sapiens	STX16
MIRT030555	hsa-miR-24-3p	Homo sapiens	CHEK1
MIRT030557	hsa-miR-24-3p	Homo sapiens	ABCB10
MIRT030558	hsa-miR-24-3p	Homo sapiens	CCL2
MIRT030559	hsa-miR-24-3p	Homo sapiens	OARD1
MIRT030560	hsa-miR-24-3p	Homo sapiens	PCGF6
MIRT030561	hsa-miR-24-3p	Homo sapiens	SNRPD3
MIRT030562	hsa-miR-24-3p	Homo sapiens	CTDSP1
MIRT030563	hsa-miR-24-3p	Homo sapiens	MAK16
MIRT030564	hsa-miR-24-3p	Homo sapiens	RNF144A
MIRT030565	hsa-miR-24-3p	Homo sapiens	TM9SF3
MIRT030566	hsa-miR-24-3p	Homo sapiens	CCDC32
MIRT030567	hsa-miR-24-3p	Homo sapiens	COMMD9
MIRT030568	hsa-miR-24-3p	Homo sapiens	SLC25A15
MIRT030569	hsa-miR-24-3p	Homo sapiens	NOP14

MIRT030570	hsa-miR-24-3p	Homo sapiens	CHD8
MIRT030571	hsa-miR-24-3p	Homo sapiens	OGFR
MIRT030572	hsa-miR-24-3p	Homo sapiens	ALG1
MIRT030573	hsa-miR-24-3p	Homo sapiens	PCK2
MIRT030574	hsa-miR-24-3p	Homo sapiens	SERGEF
MIRT030575	hsa-miR-24-3p	Homo sapiens	ANPEP
MIRT030576	hsa-miR-24-3p	Homo sapiens	FAM234A
MIRT030577	hsa-miR-24-3p	Homo sapiens	PSME3
MIRT030578	hsa-miR-24-3p	Homo sapiens	THAP12
MIRT030579	hsa-miR-24-3p	Homo sapiens	AP5M1
MIRT030580	hsa-miR-24-3p	Homo sapiens	HMGB2
MIRT030581	hsa-miR-24-3p	Homo sapiens	TNFAIP3
MIRT030582	hsa-miR-24-3p	Homo sapiens	CSTF3
MIRT030583	hsa-miR-24-3p	Homo sapiens	FOXQ1
MIRT030584	hsa-miR-24-3p	Homo sapiens	BTBD3
MIRT030585	hsa-miR-24-3p	Homo sapiens	PA2G4
MIRT030586	hsa-miR-24-3p	Homo sapiens	ZMYND19
MIRT030587	hsa-miR-24-3p	Homo sapiens	CHFR
MIRT030588	hsa-miR-24-3p	Homo sapiens	CCNB1
MIRT030589	hsa-miR-24-3p	Homo sapiens	ERBB3
MIRT030590	hsa-miR-24-3p	Homo sapiens	NEDD4L
MIRT030591	hsa-miR-24-3p	Homo sapiens	NETO2
MIRT030592	hsa-miR-24-3p	Homo sapiens	LAMTOR3
MIRT030593	hsa-miR-24-3p	Homo sapiens	MRPL27
MIRT030594	hsa-miR-24-3p	Homo sapiens	COPS7A
MIRT030595	hsa-miR-24-3p	Homo sapiens	DSC2
MIRT030596	hsa-miR-24-3p	Homo sapiens	DHCR24
MIRT030597	hsa-miR-24-3p	Homo sapiens	RPL7L1
MIRT030598	hsa-miR-24-3p	Homo sapiens	TMEM94
MIRT030599	hsa-miR-24-3p	Homo sapiens	CCAR2
MIRT030600	hsa-miR-24-3p	Homo sapiens	HIC2
MIRT030601	hsa-miR-24-3p	Homo sapiens	DTL
MIRT030602	hsa-miR-24-3p	Homo sapiens	NUBPL
MIRT030603	hsa-miR-24-3p	Homo sapiens	GMFB
MIRT030604	hsa-miR-24-3p	Homo sapiens	PLAGL2
MIRT030605	hsa-miR-24-3p	Homo sapiens	CMTM3
MIRT030606	hsa-miR-24-3p	Homo sapiens	CSK
MIRT030607	hsa-miR-24-3p	Homo sapiens	MMS19
MIRT030608	hsa-miR-24-3p	Homo sapiens	MRPS22
MIRT030610	hsa-miR-24-3p	Homo sapiens	NET1
MIRT030611	hsa-miR-24-3p	Homo sapiens	GUCD1
MIRT030612	hsa-miR-24-3p	Homo sapiens	RALA
MIRT030613	hsa-miR-24-3p	Homo sapiens	AK4
MIRT030614	hsa-miR-24-3p	Homo sapiens	KCNJ14

MIRT030615	hsa-miR-24-3p	Homo sapiens	JARID2
MIRT030616	hsa-miR-24-3p	Homo sapiens	BRD8
MIRT030617	hsa-miR-24-3p	Homo sapiens	PIM2
MIRT030618	hsa-miR-24-3p	Homo sapiens	GFOD1
MIRT030619	hsa-miR-24-3p	Homo sapiens	HDAC1
MIRT030621	hsa-miR-24-3p	Homo sapiens	NEMP1
MIRT030622	hsa-miR-24-3p	Homo sapiens	YOD1
MIRT030623	hsa-miR-24-3p	Homo sapiens	RNF11
MIRT030624	hsa-miR-24-3p	Homo sapiens	RNF2
MIRT030625	hsa-miR-24-3p	Homo sapiens	FZD5
MIRT030626	hsa-miR-24-3p	Homo sapiens	E2F1
MIRT030627	hsa-miR-24-3p	Homo sapiens	CORO1A
MIRT030628	hsa-miR-24-3p	Homo sapiens	UBC
MIRT030629	hsa-miR-24-3p	Homo sapiens	MCM4
MIRT030630	hsa-miR-24-3p	Homo sapiens	PDXK
MIRT030631	hsa-miR-24-3p	Homo sapiens	PCNA
MIRT035526	hsa-miR-24-3p	Homo sapiens	PTPN9
MIRT035527	hsa-miR-24-3p	Homo sapiens	PTPRF
MIRT035542	hsa-miR-24-3p	Homo sapiens	SH3PXD2A
MIRT035543	hsa-miR-24-3p	Homo sapiens	ARHGAP19
MIRT050365	hsa-miR-24-3p	Homo sapiens	RPS7
MIRT050366	hsa-miR-24-3p	Homo sapiens	EXOSC1
MIRT050367	hsa-miR-24-3p	Homo sapiens	AMPD2
MIRT050368	hsa-miR-24-3p	Homo sapiens	RANBP1
MIRT050369	hsa-miR-24-3p	Homo sapiens	RPRD2
MIRT050370	hsa-miR-24-3p	Homo sapiens	DARS2
MIRT050371	hsa-miR-24-3p	Homo sapiens	RPS16
MIRT050372	hsa-miR-24-3p	Homo sapiens	DEPDC1
MIRT050373	hsa-miR-24-3p	Homo sapiens	DDX5
MIRT050374	hsa-miR-24-3p	Homo sapiens	BCL7A
MIRT050375	hsa-miR-24-3p	Homo sapiens	SNX12
MIRT050376	hsa-miR-24-3p	Homo sapiens	GORASP2
MIRT050377	hsa-miR-24-3p	Homo sapiens	RIF1
MIRT050378	hsa-miR-24-3p	Homo sapiens	EEF1A1
MIRT050379	hsa-miR-24-3p	Homo sapiens	CCNG1
MIRT050380	hsa-miR-24-3p	Homo sapiens	ELP1
MIRT050382	hsa-miR-24-3p	Homo sapiens	GCLM
MIRT050383	hsa-miR-24-3p	Homo sapiens	DCAF10
MIRT052953	hsa-miR-24-3p	Homo sapiens	S100A8
MIRT053042	hsa-miR-24-3p	Homo sapiens	MXI1
MIRT053061	hsa-miR-24-3p	Homo sapiens	XIAP
MIRT053134	hsa-miR-24-3p	Homo sapiens	NOS3
MIRT053161	hsa-miR-24-3p	Homo sapiens	INSIG1
MIRT054288	hsa-miR-24-3p	Homo sapiens	MEN1

MIRT054320	hsa-miR-24-3p	Homo sapiens	LDHA
MIRT054323	hsa-miR-24-3p	Homo sapiens	LDHB
MIRT054386	hsa-miR-24-3p	Homo sapiens	JPH2
MIRT054393	hsa-miR-24-3p	Homo sapiens	DYRK2
MIRT054474	hsa-miR-24-3p	Homo sapiens	MAP3K9
MIRT054754	hsa-miR-24-3p	Homo sapiens	HMOX1
MIRT054828	hsa-miR-24-3p	Homo sapiens	PSAP
MIRT115232	hsa-miR-24-3p	Homo sapiens	ABHD2
MIRT123977	hsa-miR-24-3p	Homo sapiens	POLR3D
MIRT196600	hsa-miR-24-3p	Homo sapiens	TAOK1
MIRT249228	hsa-miR-24-3p	Homo sapiens	EIF5
MIRT256046	hsa-miR-24-3p	Homo sapiens	UBE2K
MIRT324457	hsa-miR-24-3p	Homo sapiens	ASB6
MIRT327634	hsa-miR-24-3p	Homo sapiens	ZXDB
MIRT338141	hsa-miR-24-3p	Homo sapiens	SP1
MIRT352061	hsa-miR-24-3p	Homo sapiens	BZW1
MIRT395244	hsa-miR-24-3p	Homo sapiens	MT1E
MIRT437500	hsa-miR-24-3p	Homo sapiens	MMP14
MIRT437826	hsa-miR-24-3p	Homo sapiens	AGPAT2
MIRT437970	hsa-miR-24-3p	Homo sapiens	IFNG
MIRT438418	hsa-miR-24-3p	Homo sapiens	FGFR3
MIRT438421	hsa-miR-24-3p	Homo sapiens	TACC3
MIRT438424	hsa-miR-24-3p	Homo sapiens	MAFB
MIRT438427	hsa-miR-24-3p	Homo sapiens	CCND1
MIRT438454	hsa-miR-24-3p	Homo sapiens	NCSTN
MIRT438608	hsa-miR-24-3p	Homo sapiens	WNT4
MIRT438644	hsa-miR-24-3p	Homo sapiens	NDST1
MIRT447723	hsa-miR-24-3p	Homo sapiens	RPS6KA5
MIRT447760	hsa-miR-24-3p	Homo sapiens	TTLL7
MIRT455064	hsa-miR-24-3p	Homo sapiens	ARHGAP39
MIRT455750	hsa-miR-24-3p	Homo sapiens	YRDC
MIRT457051	hsa-miR-24-3p	Homo sapiens	NEGR1
MIRT462325	hsa-miR-24-3p	Homo sapiens	SETX
MIRT464501	hsa-miR-24-3p	Homo sapiens	UCK2
MIRT465466	hsa-miR-24-3p	Homo sapiens	TOR2A
MIRT471227	hsa-miR-24-3p	Homo sapiens	PHAX
MIRT476066	hsa-miR-24-3p	Homo sapiens	GRINA
MIRT476461	hsa-miR-24-3p	Homo sapiens	GBA2
MIRT476706	hsa-miR-24-3p	Homo sapiens	FSCN1
MIRT476916	hsa-miR-24-3p	Homo sapiens	FBLIM1
MIRT477425	hsa-miR-24-3p	Homo sapiens	EMP1
MIRT477908	hsa-miR-24-3p	Homo sapiens	DVL3
MIRT478801	hsa-miR-24-3p	Homo sapiens	CRTAP
MIRT479899	hsa-miR-24-3p	Homo sapiens	CCDC117

MIRT480435	hsa-miR-24-3p	Homo sapiens	C17orf49
MIRT481095	hsa-miR-24-3p	Homo sapiens	B3GNT2
MIRT481990	hsa-miR-24-3p	Homo sapiens	AMOTL2
MIRT486982	hsa-miR-24-3p	Homo sapiens	STEAP3
MIRT489236	hsa-miR-24-3p	Homo sapiens	CLN8
MIRT490739	hsa-miR-24-3p	Homo sapiens	SRCIN1
MIRT492019	hsa-miR-24-3p	Homo sapiens	UGCG
MIRT493589	hsa-miR-24-3p	Homo sapiens	HNRNPA1
MIRT494058	hsa-miR-24-3p	Homo sapiens	DUSP7
MIRT499970	hsa-miR-24-3p	Homo sapiens	NCOA5
MIRT501272	hsa-miR-24-3p	Homo sapiens	NHS
MIRT501542	hsa-miR-24-3p	Homo sapiens	POGZ
MIRT527729	hsa-miR-24-3p	Homo sapiens	TMEM241
MIRT528420	hsa-miR-24-3p	Homo sapiens	MRPS16
MIRT528870	hsa-miR-24-3p	Homo sapiens	ATF3
MIRT530340	hsa-miR-24-3p	Homo sapiens	GABRB3
MIRT540115	hsa-miR-24-3p	Homo sapiens	KLF17
MIRT540603	hsa-miR-24-3p	Homo sapiens	CD3D
MIRT540636	hsa-miR-24-3p	Homo sapiens	SUMO1
MIRT541054	hsa-miR-24-3p	Homo sapiens	SEPHS1
MIRT541713	hsa-miR-24-3p	Homo sapiens	TMEM33
MIRT541892	hsa-miR-24-3p	Homo sapiens	LY6G5B
MIRT542947	hsa-miR-24-3p	Homo sapiens	GIGYF1
MIRT545431	hsa-miR-24-3p	Homo sapiens	SCAMP2
MIRT549513	hsa-miR-24-3p	Homo sapiens	HDDC2
MIRT556737	hsa-miR-24-3p	Homo sapiens	KLHL15
MIRT559280	hsa-miR-24-3p	Homo sapiens	AURKA
MIRT564581	hsa-miR-24-3p	Homo sapiens	ZXDA
MIRT571909	hsa-miR-24-3p	Homo sapiens	LSM14A
MIRT607045	hsa-miR-24-3p	Homo sapiens	IDS
MIRT607713	hsa-miR-24-3p	Homo sapiens	LIMS1
MIRT608083	hsa-miR-24-3p	Homo sapiens	CRISPLD2
MIRT609645	hsa-miR-24-3p	Homo sapiens	PACS2
MIRT611112	hsa-miR-24-3p	Homo sapiens	NIPA1
MIRT613671	hsa-miR-24-3p	Homo sapiens	KIAA1210
MIRT615060	hsa-miR-24-3p	Homo sapiens	CRY2
MIRT617926	hsa-miR-24-3p	Homo sapiens	ZNF783
MIRT618465	hsa-miR-24-3p	Homo sapiens	GPR55
MIRT619409	hsa-miR-24-3p	Homo sapiens	NTPCR
MIRT620694	hsa-miR-24-3p	Homo sapiens	RFTN2
MIRT621035	hsa-miR-24-3p	Homo sapiens	POLA2
MIRT622583	hsa-miR-24-3p	Homo sapiens	PRRG4
MIRT623371	hsa-miR-24-3p	Homo sapiens	LRIG2
MIRT623402	hsa-miR-24-3p	Homo sapiens	LEPROTL1

MIRT625113	hsa-miR-24-3p	Homo sapiens	SLC1A5
MIRT625326	hsa-miR-24-3p	Homo sapiens	TNFRSF13B
MIRT625414	hsa-miR-24-3p	Homo sapiens	IMP4
MIRT626607	hsa-miR-24-3p	Homo sapiens	ACAA2
MIRT626675	hsa-miR-24-3p	Homo sapiens	CISD2
MIRT626815	hsa-miR-24-3p	Homo sapiens	PRR11
MIRT626828	hsa-miR-24-3p	Homo sapiens	ZNF430
MIRT627784	hsa-miR-24-3p	Homo sapiens	RAB11FIP1
MIRT628137	hsa-miR-24-3p	Homo sapiens	HM13
MIRT628610	hsa-miR-24-3p	Homo sapiens	ZBTB3
MIRT628683	hsa-miR-24-3p	Homo sapiens	TRAF3IP1
MIRT628787	hsa-miR-24-3p	Homo sapiens	GSDMA
MIRT629019	hsa-miR-24-3p	Homo sapiens	OSBPL10
MIRT629125	hsa-miR-24-3p	Homo sapiens	APPL1
MIRT629200	hsa-miR-24-3p	Homo sapiens	PAPOLA
MIRT629234	hsa-miR-24-3p	Homo sapiens	CINP
MIRT629398	hsa-miR-24-3p	Homo sapiens	CRCP
MIRT629517	hsa-miR-24-3p	Homo sapiens	ULBP3
MIRT629559	hsa-miR-24-3p	Homo sapiens	EMP2
MIRT629570	hsa-miR-24-3p	Homo sapiens	PIGR
MIRT629748	hsa-miR-24-3p	Homo sapiens	SCD5
MIRT629797	hsa-miR-24-3p	Homo sapiens	GPR82
MIRT629813	hsa-miR-24-3p	Homo sapiens	SNRPD1
MIRT629907	hsa-miR-24-3p	Homo sapiens	SPATA5
MIRT630004	hsa-miR-24-3p	Homo sapiens	PDE6B
MIRT630034	hsa-miR-24-3p	Homo sapiens	TESMIN
MIRT630075	hsa-miR-24-3p	Homo sapiens	GRWD1
MIRT630136	hsa-miR-24-3p	Homo sapiens	ZFYVE9
MIRT630208	hsa-miR-24-3p	Homo sapiens	SVIP
MIRT630219	hsa-miR-24-3p	Homo sapiens	SORD
MIRT630250	hsa-miR-24-3p	Homo sapiens	SMTNL2
MIRT630295	hsa-miR-24-3p	Homo sapiens	PRICKLE1
MIRT630400	hsa-miR-24-3p	Homo sapiens	MYH9
MIRT630411	hsa-miR-24-3p	Homo sapiens	MOB1A
MIRT630436	hsa-miR-24-3p	Homo sapiens	KIF1C
MIRT630475	hsa-miR-24-3p	Homo sapiens	DTD2
MIRT630517	hsa-miR-24-3p	Homo sapiens	CDC73
MIRT630809	hsa-miR-24-3p	Homo sapiens	XPNPEP3
MIRT630819	hsa-miR-24-3p	Homo sapiens	YTHDC1
MIRT630833	hsa-miR-24-3p	Homo sapiens	ZNF621
MIRT630913	hsa-miR-24-3p	Homo sapiens	ZMAT2
MIRT631166	hsa-miR-24-3p	Homo sapiens	APTX
MIRT631210	hsa-miR-24-3p	Homo sapiens	DENND6B
MIRT631569	hsa-miR-24-3p	Homo sapiens	TRAF3IP2

MIRT631648	hsa-miR-24-3p	Homo sapiens	PEAK3
MIRT631698	hsa-miR-24-3p	Homo sapiens	C1QTNF6
MIRT631997	hsa-miR-24-3p	Homo sapiens	POPDC2
MIRT632015	hsa-miR-24-3p	Homo sapiens	TAF1B
MIRT632034	hsa-miR-24-3p	Homo sapiens	SPPL2A
MIRT632219	hsa-miR-24-3p	Homo sapiens	YME1L1
MIRT632351	hsa-miR-24-3p	Homo sapiens	STRN3
MIRT632358	hsa-miR-24-3p	Homo sapiens	SRRD
MIRT632426	hsa-miR-24-3p	Homo sapiens	SHOC2
MIRT632456	hsa-miR-24-3p	Homo sapiens	SGTB
MIRT632493	hsa-miR-24-3p	Homo sapiens	RBM3
MIRT632631	hsa-miR-24-3p	Homo sapiens	PARP2
MIRT632637	hsa-miR-24-3p	Homo sapiens	OSMR
MIRT632718	hsa-miR-24-3p	Homo sapiens	MSANTD4
MIRT632753	hsa-miR-24-3p	Homo sapiens	MED28
MIRT632761	hsa-miR-24-3p	Homo sapiens	MDM4
MIRT632841	hsa-miR-24-3p	Homo sapiens	IGF1
MIRT633144	hsa-miR-24-3p	Homo sapiens	FAM241A
MIRT633256	hsa-miR-24-3p	Homo sapiens	ZNF581
MIRT633260	hsa-miR-24-3p	Homo sapiens	ZNF556
MIRT633327	hsa-miR-24-3p	Homo sapiens	PRPF6
MIRT633336	hsa-miR-24-3p	Homo sapiens	GRK4
MIRT633354	hsa-miR-24-3p	Homo sapiens	TFDP2
MIRT633460	hsa-miR-24-3p	Homo sapiens	DSN1
MIRT633498	hsa-miR-24-3p	Homo sapiens	ERO1A
MIRT633534	hsa-miR-24-3p	Homo sapiens	PGBD5
MIRT633621	hsa-miR-24-3p	Homo sapiens	R3HDM2
MIRT633748	hsa-miR-24-3p	Homo sapiens	MCM9
MIRT633874	hsa-miR-24-3p	Homo sapiens	ATP6V1A
MIRT634001	hsa-miR-24-3p	Homo sapiens	SSR1
MIRT634106	hsa-miR-24-3p	Homo sapiens	ZNF8
MIRT634121	hsa-miR-24-3p	Homo sapiens	ZMYM1
MIRT634135	hsa-miR-24-3p	Homo sapiens	YWHAZ
MIRT634182	hsa-miR-24-3p	Homo sapiens	TXNDC16
MIRT634205	hsa-miR-24-3p	Homo sapiens	TMEM192
MIRT634277	hsa-miR-24-3p	Homo sapiens	TIAL1
MIRT634362	hsa-miR-24-3p	Homo sapiens	RASSF9
MIRT634446	hsa-miR-24-3p	Homo sapiens	PDE7A
MIRT634467	hsa-miR-24-3p	Homo sapiens	PAFAH1B2
MIRT634482	hsa-miR-24-3p	Homo sapiens	OR7D2
MIRT634612	hsa-miR-24-3p	Homo sapiens	IKZF3
MIRT634627	hsa-miR-24-3p	Homo sapiens	TOR1AIP2
MIRT635035	hsa-miR-24-3p	Homo sapiens	WWTR1
MIRT635140	hsa-miR-24-3p	Homo sapiens	PLEKHA2

MIRT635801	hsa-miR-24-3p	Homo sapiens	DNAJC10
MIRT636093	hsa-miR-24-3p	Homo sapiens	ZFP30
MIRT636334	hsa-miR-24-3p	Homo sapiens	PI4K2B
MIRT636711	hsa-miR-24-3p	Homo sapiens	ARSK
MIRT636762	hsa-miR-24-3p	Homo sapiens	C17orf75
MIRT636840	hsa-miR-24-3p	Homo sapiens	MBOAT1
MIRT636938	hsa-miR-24-3p	Homo sapiens	CCDC122
MIRT637028	hsa-miR-24-3p	Homo sapiens	SPTLC3
MIRT637079	hsa-miR-24-3p	Homo sapiens	SELPLG
MIRT637426	hsa-miR-24-3p	Homo sapiens	ZC3H12B
MIRT637959	hsa-miR-24-3p	Homo sapiens	NECAB3
MIRT638184	hsa-miR-24-3p	Homo sapiens	TLN1
MIRT638344	hsa-miR-24-3p	Homo sapiens	RBMS2
MIRT638571	hsa-miR-24-3p	Homo sapiens	IER5
MIRT638607	hsa-miR-24-3p	Homo sapiens	HINT1
MIRT638657	hsa-miR-24-3p	Homo sapiens	GGCX
MIRT638768	hsa-miR-24-3p	Homo sapiens	EPB41
MIRT638773	hsa-miR-24-3p	Homo sapiens	EMC7
MIRT639018	hsa-miR-24-3p	Homo sapiens	AAK1
MIRT639091	hsa-miR-24-3p	Homo sapiens	ALDOA
MIRT639575	hsa-miR-24-3p	Homo sapiens	AVL9
MIRT639828	hsa-miR-24-3p	Homo sapiens	ZKSCAN1
MIRT640336	hsa-miR-24-3p	Homo sapiens	AP5B1
MIRT640568	hsa-miR-24-3p	Homo sapiens	CPE
MIRT641826	hsa-miR-24-3p	Homo sapiens	TSC22D2
MIRT642059	hsa-miR-24-3p	Homo sapiens	KCNK2
MIRT643080	hsa-miR-24-3p	Homo sapiens	HACD4
MIRT643278	hsa-miR-24-3p	Homo sapiens	ZNF566
MIRT643443	hsa-miR-24-3p	Homo sapiens	LAX1
MIRT643866	hsa-miR-24-3p	Homo sapiens	COX20
MIRT645034	hsa-miR-24-3p	Homo sapiens	ATAD3C
MIRT645089	hsa-miR-24-3p	Homo sapiens	SLC35E2B
MIRT645155	hsa-miR-24-3p	Homo sapiens	NOL9
MIRT645990	hsa-miR-24-3p	Homo sapiens	ACP6
MIRT646444	hsa-miR-24-3p	Homo sapiens	XRCC2
MIRT646505	hsa-miR-24-3p	Homo sapiens	FAM217B
MIRT647713	hsa-miR-24-3p	Homo sapiens	NFX1
MIRT647978	hsa-miR-24-3p	Homo sapiens	PDE12
MIRT648112	hsa-miR-24-3p	Homo sapiens	PTDSS2
MIRT649180	hsa-miR-24-3p	Homo sapiens	DNPEP
MIRT649311	hsa-miR-24-3p	Homo sapiens	IGSF6
MIRT649384	hsa-miR-24-3p	Homo sapiens	TMEM19
MIRT649625	hsa-miR-24-3p	Homo sapiens	EHD2
MIRT649716	hsa-miR-24-3p	Homo sapiens	TWSG1

MIRT649845	hsa-miR-24-3p	Homo sapiens	IRAK3
MIRT650369	hsa-miR-24-3p	Homo sapiens	MOCS3
MIRT651388	hsa-miR-24-3p	Homo sapiens	ZBTB16
MIRT651610	hsa-miR-24-3p	Homo sapiens	WDFY2
MIRT652860	hsa-miR-24-3p	Homo sapiens	TAB1
MIRT653271	hsa-miR-24-3p	Homo sapiens	SNAP29
MIRT653829	hsa-miR-24-3p	Homo sapiens	SHROOM3
MIRT654023	hsa-miR-24-3p	Homo sapiens	SAMD5
MIRT654435	hsa-miR-24-3p	Homo sapiens	RASGRP3
MIRT654489	hsa-miR-24-3p	Homo sapiens	RAD54L2
MIRT654642	hsa-miR-24-3p	Homo sapiens	PTAFR
MIRT654712	hsa-miR-24-3p	Homo sapiens	PRR13
MIRT655096	hsa-miR-24-3p	Homo sapiens	PHLDA3
MIRT655326	hsa-miR-24-3p	Homo sapiens	PCYOX1
MIRT655669	hsa-miR-24-3p	Homo sapiens	NUP43
MIRT657725	hsa-miR-24-3p	Homo sapiens	GPC4
MIRT658423	hsa-miR-24-3p	Homo sapiens	FAM177A1
MIRT658703	hsa-miR-24-3p	Homo sapiens	EMC2
MIRT658849	hsa-miR-24-3p	Homo sapiens	DUSP19
MIRT658887	hsa-miR-24-3p	Homo sapiens	DRAXIN
MIRT659312	hsa-miR-24-3p	Homo sapiens	CSTF1
MIRT660234	hsa-miR-24-3p	Homo sapiens	BMP7
MIRT661045	hsa-miR-24-3p	Homo sapiens	RABAC1
MIRT661236	hsa-miR-24-3p	Homo sapiens	ARL17B
MIRT661501	hsa-miR-24-3p	Homo sapiens	EIF1AD
MIRT661659	hsa-miR-24-3p	Homo sapiens	ZNF623
MIRT661872	hsa-miR-24-3p	Homo sapiens	PDLIM5
MIRT662783	hsa-miR-24-3p	Homo sapiens	CNNM3
MIRT663096	hsa-miR-24-3p	Homo sapiens	THEM4
MIRT663668	hsa-miR-24-3p	Homo sapiens	TMEM216
MIRT663971	hsa-miR-24-3p	Homo sapiens	ZNF786
MIRT664170	hsa-miR-24-3p	Homo sapiens	APOBEC3F
MIRT664664	hsa-miR-24-3p	Homo sapiens	HEXA
MIRT664692	hsa-miR-24-3p	Homo sapiens	DBF4
MIRT664759	hsa-miR-24-3p	Homo sapiens	MESD
MIRT664772	hsa-miR-24-3p	Homo sapiens	LIAS
MIRT664862	hsa-miR-24-3p	Homo sapiens	SLC19A3
MIRT665091	hsa-miR-24-3p	Homo sapiens	CRIPT
MIRT665197	hsa-miR-24-3p	Homo sapiens	ESF1
MIRT665344	hsa-miR-24-3p	Homo sapiens	YES1
MIRT665357	hsa-miR-24-3p	Homo sapiens	XKR4
MIRT665426	hsa-miR-24-3p	Homo sapiens	WDR55
MIRT665452	hsa-miR-24-3p	Homo sapiens	WDR17
MIRT665902	hsa-miR-24-3p	Homo sapiens	TBCCD1

MIRT666256	hsa-miR-24-3p	Homo sapiens	SLC33A1
MIRT666709	hsa-miR-24-3p	Homo sapiens	RBL1
MIRT667207	hsa-miR-24-3p	Homo sapiens	NIPAL1
MIRT667223	hsa-miR-24-3p	Homo sapiens	NFE2L1
MIRT667333	hsa-miR-24-3p	Homo sapiens	MTHFD1L
MIRT667586	hsa-miR-24-3p	Homo sapiens	LONRF2
MIRT667907	hsa-miR-24-3p	Homo sapiens	ING1
MIRT668074	hsa-miR-24-3p	Homo sapiens	GMPS
MIRT668086	hsa-miR-24-3p	Homo sapiens	GMEB1
MIRT668476	hsa-miR-24-3p	Homo sapiens	EXOSC2
MIRT668507	hsa-miR-24-3p	Homo sapiens	ESYT2
MIRT668539	hsa-miR-24-3p	Homo sapiens	ERGIC1
MIRT668639	hsa-miR-24-3p	Homo sapiens	DYNLL2
MIRT669521	hsa-miR-24-3p	Homo sapiens	APOOL
MIRT669986	hsa-miR-24-3p	Homo sapiens	SSR3
MIRT670217	hsa-miR-24-3p	Homo sapiens	BAZ2B
MIRT670223	hsa-miR-24-3p	Homo sapiens	WIZ
MIRT670255	hsa-miR-24-3p	Homo sapiens	ZKSCAN3
MIRT670306	hsa-miR-24-3p	Homo sapiens	TAF8
MIRT670318	hsa-miR-24-3p	Homo sapiens	CEP57L1
MIRT670394	hsa-miR-24-3p	Homo sapiens	KCNK5
MIRT670428	hsa-miR-24-3p	Homo sapiens	ELP2
MIRT670437	hsa-miR-24-3p	Homo sapiens	REPS2
MIRT670442	hsa-miR-24-3p	Homo sapiens	SYNRG
MIRT670504	hsa-miR-24-3p	Homo sapiens	LYRM4
MIRT670512	hsa-miR-24-3p	Homo sapiens	ZSCAN22
MIRT670528	hsa-miR-24-3p	Homo sapiens	SLC9A7
MIRT670554	hsa-miR-24-3p	Homo sapiens	SHISA2
MIRT670638	hsa-miR-24-3p	Homo sapiens	BVES
MIRT670656	hsa-miR-24-3p	Homo sapiens	STX4
MIRT670685	hsa-miR-24-3p	Homo sapiens	SUGT1
MIRT670703	hsa-miR-24-3p	Homo sapiens	SLC16A13
MIRT670744	hsa-miR-24-3p	Homo sapiens	HOOK3
MIRT670798	hsa-miR-24-3p	Homo sapiens	UHRF1BP1L
MIRT670843	hsa-miR-24-3p	Homo sapiens	SFT2D2
MIRT670976	hsa-miR-24-3p	Homo sapiens	MED17
MIRT670996	hsa-miR-24-3p	Homo sapiens	PTGIS
MIRT671013	hsa-miR-24-3p	Homo sapiens	RBM22
MIRT671040	hsa-miR-24-3p	Homo sapiens	SS18
MIRT671092	hsa-miR-24-3p	Homo sapiens	DNAJC3
MIRT671168	hsa-miR-24-3p	Homo sapiens	MAPKAPK5
MIRT671222	hsa-miR-24-3p	Homo sapiens	CLSTN1
MIRT671247	hsa-miR-24-3p	Homo sapiens	TMEM41B
MIRT671251	hsa-miR-24-3p	Homo sapiens	ATP6V0E1

MIRT671287	hsa-miR-24-3p	Homo sapiens	RPL37A
MIRT671447	hsa-miR-24-3p	Homo sapiens	DNA2
MIRT671467	hsa-miR-24-3p	Homo sapiens	GPAT4
MIRT671574	hsa-miR-24-3p	Homo sapiens	FOSL2
MIRT671598	hsa-miR-24-3p	Homo sapiens	RILPL1
MIRT671609	hsa-miR-24-3p	Homo sapiens	MPIG6B
MIRT671637	hsa-miR-24-3p	Homo sapiens	FBXO36
MIRT671721	hsa-miR-24-3p	Homo sapiens	PMPCA
MIRT671766	hsa-miR-24-3p	Homo sapiens	PLA2G4A
MIRT671821	hsa-miR-24-3p	Homo sapiens	TRPM6
MIRT671831	hsa-miR-24-3p	Homo sapiens	STIL
MIRT671853	hsa-miR-24-3p	Homo sapiens	APOL2
MIRT671888	hsa-miR-24-3p	Homo sapiens	MOB3A
MIRT671912	hsa-miR-24-3p	Homo sapiens	PCDHB11
MIRT672028	hsa-miR-24-3p	Homo sapiens	ZNF70
MIRT672085	hsa-miR-24-3p	Homo sapiens	AEN
MIRT672121	hsa-miR-24-3p	Homo sapiens	ATP6V0A2
MIRT672148	hsa-miR-24-3p	Homo sapiens	PLEKHH1
MIRT672168	hsa-miR-24-3p	Homo sapiens	FANCF
MIRT672205	hsa-miR-24-3p	Homo sapiens	ZNF490
MIRT672214	hsa-miR-24-3p	Homo sapiens	DCAF7
MIRT672249	hsa-miR-24-3p	Homo sapiens	SIK2
MIRT672357	hsa-miR-24-3p	Homo sapiens	VPS8
MIRT672455	hsa-miR-24-3p	Homo sapiens	POU2F3
MIRT672519	hsa-miR-24-3p	Homo sapiens	CRX
MIRT672544	hsa-miR-24-3p	Homo sapiens	BRMS1L
MIRT672617	hsa-miR-24-3p	Homo sapiens	IGF2R
MIRT672722	hsa-miR-24-3p	Homo sapiens	S1PR2
MIRT672724	hsa-miR-24-3p	Homo sapiens	KIF18B
MIRT672826	hsa-miR-24-3p	Homo sapiens	GJD3
MIRT672879	hsa-miR-24-3p	Homo sapiens	ZSCAN29
MIRT672944	hsa-miR-24-3p	Homo sapiens	AKAP5
MIRT673004	hsa-miR-24-3p	Homo sapiens	TAF1
MIRT673027	hsa-miR-24-3p	Homo sapiens	RBBP4
MIRT673038	hsa-miR-24-3p	Homo sapiens	SGPL1
MIRT673070	hsa-miR-24-3p	Homo sapiens	AGO3
MIRT673383	hsa-miR-24-3p	Homo sapiens	ZNF124
MIRT673414	hsa-miR-24-3p	Homo sapiens	RBBP9
MIRT673420	hsa-miR-24-3p	Homo sapiens	RNF24
MIRT673425	hsa-miR-24-3p	Homo sapiens	APAF1
MIRT673481	hsa-miR-24-3p	Homo sapiens	GTF3C6
MIRT673622	hsa-miR-24-3p	Homo sapiens	VCPIP1
MIRT673629	hsa-miR-24-3p	Homo sapiens	PPM1D
MIRT673675	hsa-miR-24-3p	Homo sapiens	NUDCD2

MIRT673686	hsa-miR-24-3p	Homo sapiens	NDUFA7
MIRT673709	hsa-miR-24-3p	Homo sapiens	SLU7
MIRT673735	hsa-miR-24-3p	Homo sapiens	TCF23
MIRT673786	hsa-miR-24-3p	Homo sapiens	CDKAL1
MIRT673797	hsa-miR-24-3p	Homo sapiens	MALL
MIRT673969	hsa-miR-24-3p	Homo sapiens	INMT
MIRT674025	hsa-miR-24-3p	Homo sapiens	ANKRD9
MIRT674257	hsa-miR-24-3p	Homo sapiens	ZNF284
MIRT674319	hsa-miR-24-3p	Homo sapiens	POLR1B
MIRT674456	hsa-miR-24-3p	Homo sapiens	ULK2
MIRT674549	hsa-miR-24-3p	Homo sapiens	GREB1
MIRT674571	hsa-miR-24-3p	Homo sapiens	KIF3A
MIRT674621	hsa-miR-24-3p	Homo sapiens	TRUB2
MIRT674624	hsa-miR-24-3p	Homo sapiens	HECTD3
MIRT674837	hsa-miR-24-3p	Homo sapiens	GLRX2
MIRT674859	hsa-miR-24-3p	Homo sapiens	GINM1
MIRT674947	hsa-miR-24-3p	Homo sapiens	PEX2
MIRT675004	hsa-miR-24-3p	Homo sapiens	PPTC7
MIRT675028	hsa-miR-24-3p	Homo sapiens	SNX1
MIRT675151	hsa-miR-24-3p	Homo sapiens	NDRG1
MIRT675209	hsa-miR-24-3p	Homo sapiens	TTC9C
MIRT675427	hsa-miR-24-3p	Homo sapiens	CLEC7A
MIRT675450	hsa-miR-24-3p	Homo sapiens	SRP19
MIRT675483	hsa-miR-24-3p	Homo sapiens	SLC1A2
MIRT675508	hsa-miR-24-3p	Homo sapiens	HSD17B12
MIRT675656	hsa-miR-24-3p	Homo sapiens	COL8A1
MIRT675709	hsa-miR-24-3p	Homo sapiens	EMC3
MIRT675759	hsa-miR-24-3p	Homo sapiens	RDH10
MIRT675920	hsa-miR-24-3p	Homo sapiens	CYP51A1
MIRT675934	hsa-miR-24-3p	Homo sapiens	RAP2B
MIRT675945	hsa-miR-24-3p	Homo sapiens	NAV1
MIRT676053	hsa-miR-24-3p	Homo sapiens	ATL3
MIRT676277	hsa-miR-24-3p	Homo sapiens	ZNF260
MIRT676414	hsa-miR-24-3p	Homo sapiens	MRO
MIRT676822	hsa-miR-24-3p	Homo sapiens	TNFSF15
MIRT676866	hsa-miR-24-3p	Homo sapiens	ZNF451
MIRT676985	hsa-miR-24-3p	Homo sapiens	ZNF708
MIRT677031	hsa-miR-24-3p	Homo sapiens	ZNF107
MIRT677310	hsa-miR-24-3p	Homo sapiens	CPSF2
MIRT677401	hsa-miR-24-3p	Homo sapiens	PCNP
MIRT677430	hsa-miR-24-3p	Homo sapiens	PDF
MIRT677587	hsa-miR-24-3p	Homo sapiens	GATA6
MIRT677977	hsa-miR-24-3p	Homo sapiens	ITGB3
MIRT678135	hsa-miR-24-3p	Homo sapiens	KLLN

MIRT678398	hsa-miR-24-3p	Homo sapiens	MYPN
MIRT678593	hsa-miR-24-3p	Homo sapiens	ANAPC16
MIRT678675	hsa-miR-24-3p	Homo sapiens	SCUBE3
MIRT679058	hsa-miR-24-3p	Homo sapiens	RMDN1
MIRT679472	hsa-miR-24-3p	Homo sapiens	RHOF
MIRT679591	hsa-miR-24-3p	Homo sapiens	HUS1
MIRT680041	hsa-miR-24-3p	Homo sapiens	OSBPL2
MIRT680163	hsa-miR-24-3p	Homo sapiens	ZDHHC20
MIRT680290	hsa-miR-24-3p	Homo sapiens	AKIP1
MIRT680342	hsa-miR-24-3p	Homo sapiens	ZNF281
MIRT681090	hsa-miR-24-3p	Homo sapiens	GSTO2
MIRT686602	hsa-miR-24-3p	Homo sapiens	TMEM70
MIRT687128	hsa-miR-24-3p	Homo sapiens	QPCTL
MIRT687298	hsa-miR-24-3p	Homo sapiens	OTUD7B
MIRT689577	hsa-miR-24-3p	Homo sapiens	NUDT7
MIRT695082	hsa-miR-24-3p	Homo sapiens	ZNF17
MIRT696483	hsa-miR-24-3p	Homo sapiens	COX6B1
MIRT698752	hsa-miR-24-3p	Homo sapiens	STK4
MIRT699564	hsa-miR-24-3p	Homo sapiens	SIT1
MIRT700607	hsa-miR-24-3p	Homo sapiens	PRKCA
MIRT703925	hsa-miR-24-3p	Homo sapiens	EPG5
MIRT704352	hsa-miR-24-3p	Homo sapiens	DBR1
MIRT705787	hsa-miR-24-3p	Homo sapiens	ALDH6A1
MIRT705927	hsa-miR-24-3p	Homo sapiens	ADAM17
MIRT710028	hsa-miR-24-3p	Homo sapiens	POLL
MIRT711298	hsa-miR-24-3p	Homo sapiens	ACOX1
MIRT711504	hsa-miR-24-3p	Homo sapiens	ESCO1
MIRT711640	hsa-miR-24-3p	Homo sapiens	LIPG
MIRT716157	hsa-miR-24-3p	Homo sapiens	RBM48
MIRT719195	hsa-miR-24-3p	Homo sapiens	CASP10
MIRT720517	hsa-miR-24-3p	Homo sapiens	PCLAF
MIRT722377	hsa-miR-24-3p	Homo sapiens	KAZALD1
MIRT723133	hsa-miR-24-3p	Homo sapiens	YPEL1
MIRT725568	hsa-miR-24-3p	Homo sapiens	CPT1A
MIRT731610	hsa-miR-24-3p	Homo sapiens	NCAN
MIRT731966	hsa-miR-24-3p	Homo sapiens	COPS5
MIRT732350	hsa-miR-24-3p	Homo sapiens	PRKCH
MIRT732580	hsa-miR-24-3p	Homo sapiens	GATA3
MIRT732981	hsa-miR-24-3p	Homo sapiens	MAPK7
MIRT732998	hsa-miR-24-3p	Homo sapiens	SLC6A4
MIRT733549	hsa-miR-24-3p	Homo sapiens	PRDX6
MIRT733591	hsa-miR-24-3p	Homo sapiens	TRIM11
MIRT733625	hsa-miR-24-3p	Homo sapiens	ABCB9
MIRT733637	hsa-miR-24-3p	Homo sapiens	ATG4A

MIRT733710	hsa-miR-24-3p	Homo sapiens	IL18
MIRT733711	hsa-miR-24-3p	Homo sapiens	IL1B
MIRT733712	hsa-miR-24-3p	Homo sapiens	TNF
MIRT733713	hsa-miR-24-3p	Homo sapiens	CCL3
MIRT733714	hsa-miR-24-3p	Homo sapiens	CCL4
MIRT734491	hsa-miR-24-3p	Homo sapiens	BCAR1
MIRT734631	hsa-miR-24-3p	Homo sapiens	MT1M
MIRT734677	hsa-miR-24-3p	Homo sapiens	TP53
MIRT735050	hsa-miR-24-3p	Homo sapiens	TNK2
MIRT735355	hsa-miR-24-3p	Homo sapiens	PDGFRB
MIRT735427	hsa-miR-24-3p	Homo sapiens	FBXW7
MIRT739219	hsa-miR-24-3p	Homo sapiens	AMBRA1
MIRT739220	hsa-miR-24-3p	Homo sapiens	APOBEC3C
MIRT739221	hsa-miR-24-3p	Homo sapiens	BBC3
MIRT739222	hsa-miR-24-3p	Homo sapiens	C8orf58
MIRT739223	hsa-miR-24-3p	Homo sapiens	CAPN15
MIRT739224	hsa-miR-24-3p	Homo sapiens	DBNDD1
MIRT739225	hsa-miR-24-3p	Homo sapiens	EREG
MIRT739226	hsa-miR-24-3p	Homo sapiens	LMNB2
MIRT739227	hsa-miR-24-3p	Homo sapiens	LRRC58
MIRT739228	hsa-miR-24-3p	Homo sapiens	MAP2K7
MIRT739229	hsa-miR-24-3p	Homo sapiens	PKMYT1
MIRT739230	hsa-miR-24-3p	Homo sapiens	PRSS8
MIRT739231	hsa-miR-24-3p	Homo sapiens	RRAS
MIRT739232	hsa-miR-24-3p	Homo sapiens	S100A16
MIRT739233	hsa-miR-24-3p	Homo sapiens	SETD5
MIRT739234	hsa-miR-24-3p	Homo sapiens	SETD7
MIRT739235	hsa-miR-24-3p	Homo sapiens	SLIT3
MIRT739236	hsa-miR-24-3p	Homo sapiens	TOMM40
MIRT739237	hsa-miR-24-3p	Homo sapiens	ZNF516
MIRT764049	hsa-miR-24-3p	Homo sapiens	ALOX5AP
MIRT764050	hsa-miR-24-3p	Homo sapiens	DCP1A
MIRT764051	hsa-miR-24-3p	Homo sapiens	FAM210B
MIRT764052	hsa-miR-24-3p	Homo sapiens	FLCN
MIRT764053	hsa-miR-24-3p	Homo sapiens	MIEF2
MIRT764054	hsa-miR-24-3p	Homo sapiens	RAB5C
MIRT764055	hsa-miR-24-3p	Homo sapiens	SMYD4
MIRT764056	hsa-miR-24-3p	Homo sapiens	TBC1D14
MIRT764057	hsa-miR-24-3p	Homo sapiens	WASF2
MIRT764058	hsa-miR-24-3p	Homo sapiens	ZNF106
MIRT784309	hsa-miR-24-3p	Homo sapiens	CCDC69
MIRT784310	hsa-miR-24-3p	Homo sapiens	FPR1
MIRT784311	hsa-miR-24-3p	Homo sapiens	KCNJ5
MIRT784312	hsa-miR-24-3p	Homo sapiens	ZC3H15

MIRT790198	hsa-miR-24-3p	Homo sapiens	RASSF4
MIRT000002	hsa-miR-20a-5p	Homo sapiens	HIF1A
MIRT000178	hsa-miR-20a-5p	Homo sapiens	TCEAL1
MIRT000179	hsa-miR-20a-5p	Homo sapiens	CCND1
MIRT000180	hsa-miR-20a-5p	Homo sapiens	E2F1
MIRT000181	hsa-miR-20a-5p	Homo sapiens	BMPR2
MIRT000597	hsa-miR-20a-5p	Homo sapiens	CDKN1A
MIRT001785	hsa-miR-20a-5p	Homo sapiens	TGFBR2
MIRT003010	hsa-miR-20a-5p	Homo sapiens	MAP3K12
MIRT003011	hsa-miR-20a-5p	Homo sapiens	BCL2
MIRT003012	hsa-miR-20a-5p	Homo sapiens	MEF2D
MIRT003369	hsa-miR-20a-5p	Homo sapiens	PTEN
MIRT003382	hsa-miR-20a-5p	Homo sapiens	APP
MIRT003742	hsa-miR-20a-5p	Homo sapiens	RUNX1
MIRT003903	hsa-miR-20a-5p	Homo sapiens	NRAS
MIRT004450	hsa-miR-20a-5p	Homo sapiens	VEGFA
MIRT004570	hsa-miR-20a-5p	Homo sapiens	BCL2L11
MIRT004711	hsa-miR-20a-5p	Homo sapiens	MUC17
MIRT005289	hsa-miR-20a-5p	Homo sapiens	MYC
MIRT005481	hsa-miR-20a-5p	Homo sapiens	BNIP2
MIRT005627	hsa-miR-20a-5p	Homo sapiens	THBS1
MIRT005631	hsa-miR-20a-5p	Homo sapiens	SMAD4
MIRT005854	hsa-miR-20a-5p	Homo sapiens	CCND2
MIRT005855	hsa-miR-20a-5p	Homo sapiens	E2F3
MIRT005856	hsa-miR-20a-5p	Homo sapiens	MAPK9
MIRT005857	hsa-miR-20a-5p	Homo sapiens	RB1
MIRT005858	hsa-miR-20a-5p	Homo sapiens	RBL1
MIRT005859	hsa-miR-20a-5p	Homo sapiens	RBL2
MIRT005860	hsa-miR-20a-5p	Homo sapiens	WEE1
MIRT006178	hsa-miR-20a-5p	Homo sapiens	IRF2
MIRT006180	hsa-miR-20a-5p	Homo sapiens	KIT
MIRT006289	hsa-miR-20a-5p	Homo sapiens	EGLN3
MIRT006754	hsa-miR-20a-5p	Homo sapiens	PPARG
MIRT006755	hsa-miR-20a-5p	Homo sapiens	BAMBI
MIRT006756	hsa-miR-20a-5p	Homo sapiens	CRIM1
MIRT006772	hsa-miR-20a-5p	Homo sapiens	MAP2K3
MIRT007002	hsa-miR-20a-5p	Homo sapiens	PURA
MIRT031081	hsa-miR-20a-5p	Homo sapiens	JAK1
MIRT031082	hsa-miR-20a-5p	Homo sapiens	ARHGAP12
MIRT031083	hsa-miR-20a-5p	Homo sapiens	TSG101
MIRT035531	hsa-miR-20a-5p	Homo sapiens	SIRPA
MIRT050476	hsa-miR-20a-5p	Homo sapiens	PHF8

MIRT050477	hsa-miR-20a-5p	Homo sapiens	GPATCH11
MIRT050478	hsa-miR-20a-5p	Homo sapiens	RPRD1A
MIRT050479	hsa-miR-20a-5p	Homo sapiens	ATP8B2
MIRT050480	hsa-miR-20a-5p	Homo sapiens	PSMD2
MIRT050481	hsa-miR-20a-5p	Homo sapiens	INSIG1
MIRT050482	hsa-miR-20a-5p	Homo sapiens	RTN2
MIRT050483	hsa-miR-20a-5p	Homo sapiens	TCEA1
MIRT050484	hsa-miR-20a-5p	Homo sapiens	PLEKHM3
MIRT050485	hsa-miR-20a-5p	Homo sapiens	RPS10
MIRT050486	hsa-miR-20a-5p	Homo sapiens	ALDH18A1
MIRT050487	hsa-miR-20a-5p	Homo sapiens	UEVLD
MIRT050488	hsa-miR-20a-5p	Homo sapiens	FGF7
MIRT050489	hsa-miR-20a-5p	Homo sapiens	SSRP1
MIRT050490	hsa-miR-20a-5p	Homo sapiens	COX5A
MIRT050491	hsa-miR-20a-5p	Homo sapiens	AGO1
MIRT050492	hsa-miR-20a-5p	Homo sapiens	KIAA0100
MIRT050493	hsa-miR-20a-5p	Homo sapiens	FHL3
MIRT050494	hsa-miR-20a-5p	Homo sapiens	GDI2
MIRT050495	hsa-miR-20a-5p	Homo sapiens	INTS3
MIRT050496	hsa-miR-20a-5p	Homo sapiens	HMG20A
MIRT050497	hsa-miR-20a-5p	Homo sapiens	NAXE
MIRT050498	hsa-miR-20a-5p	Homo sapiens	PRKD3
MIRT050499	hsa-miR-20a-5p	Homo sapiens	AP3S2
MIRT050500	hsa-miR-20a-5p	Homo sapiens	PCNX4
MIRT050501	hsa-miR-20a-5p	Homo sapiens	IKZF5
MIRT050502	hsa-miR-20a-5p	Homo sapiens	TMEM97
MIRT050504	hsa-miR-20a-5p	Homo sapiens	RPA2
MIRT050505	hsa-miR-20a-5p	Homo sapiens	PHYH
MIRT050506	hsa-miR-20a-5p	Homo sapiens	KIF2C
MIRT050507	hsa-miR-20a-5p	Homo sapiens	DDX5
MIRT050508	hsa-miR-20a-5p	Homo sapiens	DTX2
MIRT050509	hsa-miR-20a-5p	Homo sapiens	MPHOSPH8
MIRT050510	hsa-miR-20a-5p	Homo sapiens	HAUS2
MIRT050511	hsa-miR-20a-5p	Homo sapiens	ZNF331
MIRT050512	hsa-miR-20a-5p	Homo sapiens	PPP6R3
MIRT050513	hsa-miR-20a-5p	Homo sapiens	METTL22
MIRT050514	hsa-miR-20a-5p	Homo sapiens	PPAN
MIRT050515	hsa-miR-20a-5p	Homo sapiens	FBL
MIRT050516	hsa-miR-20a-5p	Homo sapiens	RPL31
MIRT050518	hsa-miR-20a-5p	Homo sapiens	TOMM20
MIRT050519	hsa-miR-20a-5p	Homo sapiens	NCOR2
MIRT050520	hsa-miR-20a-5p	Homo sapiens	PSD3
MIRT050521	hsa-miR-20a-5p	Homo sapiens	AP3D1
MIRT050522	hsa-miR-20a-5p	Homo sapiens	TBC1D15

MIRT050523	hsa-miR-20a-5p	Homo sapiens	DPY19L4
MIRT050524	hsa-miR-20a-5p	Homo sapiens	PTPN23
MIRT050525	hsa-miR-20a-5p	Homo sapiens	C11orf68
MIRT050526	hsa-miR-20a-5p	Homo sapiens	CTR9
MIRT050527	hsa-miR-20a-5p	Homo sapiens	PAIP1
MIRT050528	hsa-miR-20a-5p	Homo sapiens	L2HGDH
MIRT050529	hsa-miR-20a-5p	Homo sapiens	CDT1
MIRT050530	hsa-miR-20a-5p	Homo sapiens	RPL30
MIRT050531	hsa-miR-20a-5p	Homo sapiens	MRS2
MIRT050532	hsa-miR-20a-5p	Homo sapiens	TMX4
MIRT050533	hsa-miR-20a-5p	Homo sapiens	LAMTOR1
MIRT050534	hsa-miR-20a-5p	Homo sapiens	ADGRL3
MIRT050535	hsa-miR-20a-5p	Homo sapiens	RBM10
MIRT050536	hsa-miR-20a-5p	Homo sapiens	ADGRE2
MIRT050537	hsa-miR-20a-5p	Homo sapiens	FBXO3
MIRT050538	hsa-miR-20a-5p	Homo sapiens	MLXIP
MIRT050539	hsa-miR-20a-5p	Homo sapiens	RNGTT
MIRT050540	hsa-miR-20a-5p	Homo sapiens	MAD1L1
MIRT050541	hsa-miR-20a-5p	Homo sapiens	DLC1
MIRT050542	hsa-miR-20a-5p	Homo sapiens	NUP214
MIRT050543	hsa-miR-20a-5p	Homo sapiens	PAQR5
MIRT050544	hsa-miR-20a-5p	Homo sapiens	BTBD2
MIRT050545	hsa-miR-20a-5p	Homo sapiens	XYLT2
MIRT050546	hsa-miR-20a-5p	Homo sapiens	ZNF398
MIRT050547	hsa-miR-20a-5p	Homo sapiens	CEP120
MIRT050548	hsa-miR-20a-5p	Homo sapiens	IL17RC
MIRT050549	hsa-miR-20a-5p	Homo sapiens	UBE2C
MIRT050550	hsa-miR-20a-5p	Homo sapiens	PGK1
MIRT050551	hsa-miR-20a-5p	Homo sapiens	ORMDL3
MIRT050552	hsa-miR-20a-5p	Homo sapiens	TUBB
MIRT050553	hsa-miR-20a-5p	Homo sapiens	TDRD3
MIRT050554	hsa-miR-20a-5p	Homo sapiens	DLG5
MIRT050555	hsa-miR-20a-5p	Homo sapiens	VEZF1
MIRT050556	hsa-miR-20a-5p	Homo sapiens	CCDC88C
MIRT050557	hsa-miR-20a-5p	Homo sapiens	USP10
MIRT050558	hsa-miR-20a-5p	Homo sapiens	KIAA1191
MIRT050559	hsa-miR-20a-5p	Homo sapiens	STAT3
MIRT050560	hsa-miR-20a-5p	Homo sapiens	GATA6
MIRT050561	hsa-miR-20a-5p	Homo sapiens	RPL18A
MIRT050562	hsa-miR-20a-5p	Homo sapiens	SARAF
MIRT050564	hsa-miR-20a-5p	Homo sapiens	CTSA
MIRT050565	hsa-miR-20a-5p	Homo sapiens	ABCA3
MIRT050566	hsa-miR-20a-5p	Homo sapiens	MRPL13
MIRT050567	hsa-miR-20a-5p	Homo sapiens	MAN1C1

MIRT050568	hsa-miR-20a-5p	Homo sapiens	AGO4
MIRT050569	hsa-miR-20a-5p	Homo sapiens	BACH1
MIRT050570	hsa-miR-20a-5p	Homo sapiens	RFC3
MIRT050571	hsa-miR-20a-5p	Homo sapiens	ARHGEF7
MIRT050572	hsa-miR-20a-5p	Homo sapiens	GPN2
MIRT050573	hsa-miR-20a-5p	Homo sapiens	LDHB
MIRT050574	hsa-miR-20a-5p	Homo sapiens	PTPRS
MIRT050575	hsa-miR-20a-5p	Homo sapiens	PPP2R1A
MIRT050576	hsa-miR-20a-5p	Homo sapiens	CDK16
MIRT050577	hsa-miR-20a-5p	Homo sapiens	WBP4
MIRT050578	hsa-miR-20a-5p	Homo sapiens	CCNB1
MIRT050579	hsa-miR-20a-5p	Homo sapiens	POGZ
MIRT050580	hsa-miR-20a-5p	Homo sapiens	KLHL15
MIRT050582	hsa-miR-20a-5p	Homo sapiens	FLNA
MIRT050583	hsa-miR-20a-5p	Homo sapiens	PLXNA1
MIRT050585	hsa-miR-20a-5p	Homo sapiens	MANEAL
MIRT050586	hsa-miR-20a-5p	Homo sapiens	NUP188
MIRT050587	hsa-miR-20a-5p	Homo sapiens	ECI1
MIRT050588	hsa-miR-20a-5p	Homo sapiens	NCOA3
MIRT050589	hsa-miR-20a-5p	Homo sapiens	MORF4L2
MIRT050590	hsa-miR-20a-5p	Homo sapiens	ATL3
MIRT050591	hsa-miR-20a-5p	Homo sapiens	FOXJ3
MIRT050592	hsa-miR-20a-5p	Homo sapiens	PRRC2C
MIRT050593	hsa-miR-20a-5p	Homo sapiens	RPL21
MIRT050594	hsa-miR-20a-5p	Homo sapiens	SELENBP1
MIRT050595	hsa-miR-20a-5p	Homo sapiens	YBX1
MIRT050596	hsa-miR-20a-5p	Homo sapiens	B4GALT2
MIRT050597	hsa-miR-20a-5p	Homo sapiens	PYGB
MIRT050598	hsa-miR-20a-5p	Homo sapiens	AKR7A2
MIRT050599	hsa-miR-20a-5p	Homo sapiens	C9orf78
MIRT050600	hsa-miR-20a-5p	Homo sapiens	STIL
MIRT050601	hsa-miR-20a-5p	Homo sapiens	CDK19
MIRT050602	hsa-miR-20a-5p	Homo sapiens	KDM4D
MIRT050603	hsa-miR-20a-5p	Homo sapiens	UQCRC1
MIRT050604	hsa-miR-20a-5p	Homo sapiens	RUFY2
MIRT050605	hsa-miR-20a-5p	Homo sapiens	RPS27
MIRT050606	hsa-miR-20a-5p	Homo sapiens	BTN3A1
MIRT050607	hsa-miR-20a-5p	Homo sapiens	PBXIP1
MIRT050608	hsa-miR-20a-5p	Homo sapiens	ARFGEF2
MIRT050609	hsa-miR-20a-5p	Homo sapiens	NUDT21
MIRT050610	hsa-miR-20a-5p	Homo sapiens	NETO2
MIRT050611	hsa-miR-20a-5p	Homo sapiens	SLC25A28
MIRT050612	hsa-miR-20a-5p	Homo sapiens	NAP1L1
MIRT050613	hsa-miR-20a-5p	Homo sapiens	PHC1

MIRT050614	hsa-miR-20a-5p	Homo sapiens	ZNF706
MIRT050615	hsa-miR-20a-5p	Homo sapiens	CCDC47
MIRT050616	hsa-miR-20a-5p	Homo sapiens	ARPC2
MIRT050617	hsa-miR-20a-5p	Homo sapiens	EIF4G2
MIRT050618	hsa-miR-20a-5p	Homo sapiens	MAGOHB
MIRT050619	hsa-miR-20a-5p	Homo sapiens	ZNF598
MIRT050620	hsa-miR-20a-5p	Homo sapiens	P3H4
MIRT050621	hsa-miR-20a-5p	Homo sapiens	CERS2
MIRT050622	hsa-miR-20a-5p	Homo sapiens	LYPD6
MIRT050623	hsa-miR-20a-5p	Homo sapiens	HEXIM1
MIRT050624	hsa-miR-20a-5p	Homo sapiens	WAC
MIRT050625	hsa-miR-20a-5p	Homo sapiens	ZNFX1
MIRT050626	hsa-miR-20a-5p	Homo sapiens	RBM12B
MIRT052914	hsa-miR-20a-5p	Homo sapiens	LIMK1
MIRT052971	hsa-miR-20a-5p	Homo sapiens	PHLPP2
MIRT053007	hsa-miR-20a-5p	Homo sapiens	GJA1
MIRT053023	hsa-miR-20a-5p	Homo sapiens	DUSP2
MIRT053109	hsa-miR-20a-5p	Homo sapiens	ITGB8
MIRT053159	hsa-miR-20a-5p	Homo sapiens	SMAD7
MIRT053208	hsa-miR-20a-5p	Homo sapiens	MAP3K5
MIRT053332	hsa-miR-20a-5p	Homo sapiens	MCL1
MIRT053505	hsa-miR-20a-5p	Homo sapiens	TP53INP1
MIRT053563	hsa-miR-20a-5p	Homo sapiens	EGR2
MIRT054860	hsa-miR-20a-5p	Homo sapiens	ABL2
MIRT055020	hsa-miR-20a-5p	Homo sapiens	TPRG1L
MIRT055382	hsa-miR-20a-5p	Homo sapiens	SHOC2
MIRT055649	hsa-miR-20a-5p	Homo sapiens	WDR37
MIRT056476	hsa-miR-20a-5p	Homo sapiens	PFKP
MIRT056811	hsa-miR-20a-5p	Homo sapiens	REEP3
MIRT057384	hsa-miR-20a-5p	Homo sapiens	TNKS2
MIRT057822	hsa-miR-20a-5p	Homo sapiens	SLC30A7
MIRT059186	hsa-miR-20a-5p	Homo sapiens	CRY2
MIRT060075	hsa-miR-20a-5p	Homo sapiens	TMEM138
MIRT060678	hsa-miR-20a-5p	Homo sapiens	KLHL20
MIRT061181	hsa-miR-20a-5p	Homo sapiens	MED17
MIRT061789	hsa-miR-20a-5p	Homo sapiens	PPP1R15B
MIRT063054	hsa-miR-20a-5p	Homo sapiens	ULK1
MIRT063434	hsa-miR-20a-5p	Homo sapiens	SKI
MIRT064435	hsa-miR-20a-5p	Homo sapiens	GPR137B
MIRT064797	hsa-miR-20a-5p	Homo sapiens	ZBTB18
MIRT065364	hsa-miR-20a-5p	Homo sapiens	TMBIM6
MIRT065670	hsa-miR-20a-5p	Homo sapiens	ACVR1B
MIRT065858	hsa-miR-20a-5p	Homo sapiens	GDF11
MIRT065886	hsa-miR-20a-5p	Homo sapiens	RAB5B

MIRT067229	hsa-miR-20a-5p	Homo sapiens	FOXJ2
MIRT068492	hsa-miR-20a-5p	Homo sapiens	NHLRC3
MIRT070839	hsa-miR-20a-5p	Homo sapiens	EIF2S1
MIRT070995	hsa-miR-20a-5p	Homo sapiens	SMOC1
MIRT071324	hsa-miR-20a-5p	Homo sapiens	CMPK1
MIRT071903	hsa-miR-20a-5p	Homo sapiens	ZFYVE9
MIRT072247	hsa-miR-20a-5p	Homo sapiens	B2M
MIRT072567	hsa-miR-20a-5p	Homo sapiens	USP3
MIRT073117	hsa-miR-20a-5p	Homo sapiens	UBE2Q2
MIRT073374	hsa-miR-20a-5p	Homo sapiens	ABHD2
MIRT073407	hsa-miR-20a-5p	Homo sapiens	SEMA4B
MIRT074789	hsa-miR-20a-5p	Homo sapiens	CYLD
MIRT074891	hsa-miR-20a-5p	Homo sapiens	CHD9
MIRT075775	hsa-miR-20a-5p	Homo sapiens	KIAA0513
MIRT076177	hsa-miR-20a-5p	Homo sapiens	GID4
MIRT077064	hsa-miR-20a-5p	Homo sapiens	KRT10
MIRT077831	hsa-miR-20a-5p	Homo sapiens	MINK1
MIRT078811	hsa-miR-20a-5p	Homo sapiens	UNK
MIRT079344	hsa-miR-20a-5p	Homo sapiens	CCDC137
MIRT079411	hsa-miR-20a-5p	Homo sapiens	FOXK2
MIRT079772	hsa-miR-20a-5p	Homo sapiens	CABLES1
MIRT080178	hsa-miR-20a-5p	Homo sapiens	PRKACB
MIRT080848	hsa-miR-20a-5p	Homo sapiens	RAB12
MIRT081117	hsa-miR-20a-5p	Homo sapiens	LDLR
MIRT081198	hsa-miR-20a-5p	Homo sapiens	MIDN
MIRT081981	hsa-miR-20a-5p	Homo sapiens	GRAMD1A
MIRT082290	hsa-miR-20a-5p	Homo sapiens	FNBP1L
MIRT083739	hsa-miR-20a-5p	Homo sapiens	PARD6B
MIRT083960	hsa-miR-20a-5p	Homo sapiens	RAB22A
MIRT084344	hsa-miR-20a-5p	Homo sapiens	RRM2
MIRT085173	hsa-miR-20a-5p	Homo sapiens	SLC5A3
MIRT085375	hsa-miR-20a-5p	Homo sapiens	SPOPL
MIRT085867	hsa-miR-20a-5p	Homo sapiens	TANC1
MIRT086425	hsa-miR-20a-5p	Homo sapiens	NABP1
MIRT087604	hsa-miR-20a-5p	Homo sapiens	ATG16L1
MIRT088033	hsa-miR-20a-5p	Homo sapiens	UBXN2A
MIRT088337	hsa-miR-20a-5p	Homo sapiens	MAPRE3
MIRT090633	hsa-miR-20a-5p	Homo sapiens	U2SURP
MIRT092685	hsa-miR-20a-5p	Homo sapiens	C3orf38
MIRT093800	hsa-miR-20a-5p	Homo sapiens	KLF3
MIRT093941	hsa-miR-20a-5p	Homo sapiens	SLAIN2
MIRT095200	hsa-miR-20a-5p	Homo sapiens	SMAD5
MIRT095719	hsa-miR-20a-5p	Homo sapiens	ANKH
MIRT095997	hsa-miR-20a-5p	Homo sapiens	ATP6V0E1

MIRT096308	hsa-miR-20a-5p	Homo sapiens	SQSTM1
MIRT097128	hsa-miR-20a-5p	Homo sapiens	FCHO2
MIRT097603	hsa-miR-20a-5p	Homo sapiens	POLR3G
MIRT097649	hsa-miR-20a-5p	Homo sapiens	LYSMD3
MIRT099308	hsa-miR-20a-5p	Homo sapiens	QKI
MIRT099355	hsa-miR-20a-5p	Homo sapiens	C6orf120
MIRT099824	hsa-miR-20a-5p	Homo sapiens	SOX4
MIRT100277	hsa-miR-20a-5p	Homo sapiens	MICB
MIRT100454	hsa-miR-20a-5p	Homo sapiens	ZBTB9
MIRT100945	hsa-miR-20a-5p	Homo sapiens	CENPQ
MIRT102221	hsa-miR-20a-5p	Homo sapiens	HBP1
MIRT102294	hsa-miR-20a-5p	Homo sapiens	DNAJB9
MIRT103189	hsa-miR-20a-5p	Homo sapiens	SP4
MIRT104161	hsa-miR-20a-5p	Homo sapiens	PHTF2
MIRT104394	hsa-miR-20a-5p	Homo sapiens	ANKIB1
MIRT108651	hsa-miR-20a-5p	Homo sapiens	ZBTB33
MIRT108719	hsa-miR-20a-5p	Homo sapiens	XIAP
MIRT110266	hsa-miR-20a-5p	Homo sapiens	GBF1
MIRT112089	hsa-miR-20a-5p	Homo sapiens	TIMM17A
MIRT115779	hsa-miR-20a-5p	Homo sapiens	CAPN15
MIRT121800	hsa-miR-20a-5p	Homo sapiens	GRPEL2
MIRT122363	hsa-miR-20a-5p	Homo sapiens	RGMB
MIRT124125	hsa-miR-20a-5p	Homo sapiens	GINS4
MIRT125725	hsa-miR-20a-5p	Homo sapiens	TRIM8
MIRT126308	hsa-miR-20a-5p	Homo sapiens	ACADSB
MIRT126344	hsa-miR-20a-5p	Homo sapiens	ZRANB1
MIRT126550	hsa-miR-20a-5p	Homo sapiens	MASTL
MIRT127161	hsa-miR-20a-5p	Homo sapiens	VPS26A
MIRT129131	hsa-miR-20a-5p	Homo sapiens	ARCN1
MIRT130070	hsa-miR-20a-5p	Homo sapiens	TXNIP
MIRT132394	hsa-miR-20a-5p	Homo sapiens	PPP1R12B
MIRT133313	hsa-miR-20a-5p	Homo sapiens	ORAI1
MIRT134275	hsa-miR-20a-5p	Homo sapiens	DNM1L
MIRT135706	hsa-miR-20a-5p	Homo sapiens	PIP4K2C
MIRT135793	hsa-miR-20a-5p	Homo sapiens	GNS
MIRT136562	hsa-miR-20a-5p	Homo sapiens	TXLNA
MIRT138110	hsa-miR-20a-5p	Homo sapiens	BRMS1L
MIRT138348	hsa-miR-20a-5p	Homo sapiens	FRMD6
MIRT138792	hsa-miR-20a-5p	Homo sapiens	SUSD6
MIRT140626	hsa-miR-20a-5p	Homo sapiens	PLEKHO2
MIRT140794	hsa-miR-20a-5p	Homo sapiens	SMAD6
MIRT141126	hsa-miR-20a-5p	Homo sapiens	SCAMP5
MIRT141698	hsa-miR-20a-5p	Homo sapiens	RCCD1
MIRT142095	hsa-miR-20a-5p	Homo sapiens	CCP110

MIRT142381	hsa-miR-20a-5p	Homo sapiens	TNRC6A
MIRT144207	hsa-miR-20a-5p	Homo sapiens	SNTB2
MIRT144294	hsa-miR-20a-5p	Homo sapiens	NFAT5
MIRT144978	hsa-miR-20a-5p	Homo sapiens	PAFAH1B1
MIRT145019	hsa-miR-20a-5p	Homo sapiens	TNFAIP1
MIRT145597	hsa-miR-20a-5p	Homo sapiens	LASP1
MIRT146071	hsa-miR-20a-5p	Homo sapiens	RUNDC1
MIRT147118	hsa-miR-20a-5p	Homo sapiens	MAP3K3
MIRT147273	hsa-miR-20a-5p	Homo sapiens	KPNA2
MIRT147921	hsa-miR-20a-5p	Homo sapiens	CAMTA1
MIRT148868	hsa-miR-20a-5p	Homo sapiens	ANKRD12
MIRT151690	hsa-miR-20a-5p	Homo sapiens	CHAF1A
MIRT151799	hsa-miR-20a-5p	Homo sapiens	BLOC1S3
MIRT151853	hsa-miR-20a-5p	Homo sapiens	ARHGAP35
MIRT151932	hsa-miR-20a-5p	Homo sapiens	TBC1D17
MIRT152367	hsa-miR-20a-5p	Homo sapiens	ARHGEF18
MIRT152673	hsa-miR-20a-5p	Homo sapiens	POFUT1
MIRT153327	hsa-miR-20a-5p	Homo sapiens	MAVS
MIRT153454	hsa-miR-20a-5p	Homo sapiens	TTPAL
MIRT153969	hsa-miR-20a-5p	Homo sapiens	PRNP
MIRT155231	hsa-miR-20a-5p	Homo sapiens	IFNAR2
MIRT155333	hsa-miR-20a-5p	Homo sapiens	IFNAR1
MIRT155886	hsa-miR-20a-5p	Homo sapiens	SIK1
MIRT156404	hsa-miR-20a-5p	Homo sapiens	RAPGEF4
MIRT156640	hsa-miR-20a-5p	Homo sapiens	C2orf69
MIRT157145	hsa-miR-20a-5p	Homo sapiens	FAM117B
MIRT157585	hsa-miR-20a-5p	Homo sapiens	MTMR3
MIRT158288	hsa-miR-20a-5p	Homo sapiens	ASB1
MIRT158573	hsa-miR-20a-5p	Homo sapiens	TNRC6B
MIRT159105	hsa-miR-20a-5p	Homo sapiens	NRBP1
MIRT159399	hsa-miR-20a-5p	Homo sapiens	FEZ2
MIRT160004	hsa-miR-20a-5p	Homo sapiens	TET3
MIRT164198	hsa-miR-20a-5p	Homo sapiens	GAB1
MIRT164524	hsa-miR-20a-5p	Homo sapiens	MSMO1
MIRT164659	hsa-miR-20a-5p	Homo sapiens	NSD2
MIRT164720	hsa-miR-20a-5p	Homo sapiens	ADD1
MIRT166062	hsa-miR-20a-5p	Homo sapiens	FAF2
MIRT167840	hsa-miR-20a-5p	Homo sapiens	HECA
MIRT168216	hsa-miR-20a-5p	Homo sapiens	BTN3A2
MIRT169663	hsa-miR-20a-5p	Homo sapiens	AGFG2
MIRT170564	hsa-miR-20a-5p	Homo sapiens	CASP2
MIRT170849	hsa-miR-20a-5p	Homo sapiens	TAX1BP1
MIRT172197	hsa-miR-20a-5p	Homo sapiens	OXR1
MIRT173112	hsa-miR-20a-5p	Homo sapiens	E2F5

MIRT173688	hsa-miR-20a-5p	Homo sapiens	PRPF4
MIRT175379	hsa-miR-20a-5p	Homo sapiens	ACSL4
MIRT175594	hsa-miR-20a-5p	Homo sapiens	OCRL
MIRT175644	hsa-miR-20a-5p	Homo sapiens	PHF6
MIRT176079	hsa-miR-20a-5p	Homo sapiens	CHIC1
MIRT178062	hsa-miR-20a-5p	Homo sapiens	SAMD8
MIRT182517	hsa-miR-20a-5p	Homo sapiens	ZBTB37
MIRT187472	hsa-miR-20a-5p	Homo sapiens	PCBP2
MIRT188183	hsa-miR-20a-5p	Homo sapiens	DYRK2
MIRT194849	hsa-miR-20a-5p	Homo sapiens	UBFD1
MIRT199106	hsa-miR-20a-5p	Homo sapiens	ZNF532
MIRT199283	hsa-miR-20a-5p	Homo sapiens	SH3GLB1
MIRT200924	hsa-miR-20a-5p	Homo sapiens	ZNF264
MIRT201019	hsa-miR-20a-5p	Homo sapiens	ZNF805
MIRT205046	hsa-miR-20a-5p	Homo sapiens	CREB1
MIRT205281	hsa-miR-20a-5p	Homo sapiens	STK11IP
MIRT206192	hsa-miR-20a-5p	Homo sapiens	RAB10
MIRT208975	hsa-miR-20a-5p	Homo sapiens	SKIL
MIRT213203	hsa-miR-20a-5p	Homo sapiens	REST
MIRT213321	hsa-miR-20a-5p	Homo sapiens	KIAA0232
MIRT216423	hsa-miR-20a-5p	Homo sapiens	SERF1A
MIRT216448	hsa-miR-20a-5p	Homo sapiens	SERF1B
MIRT216661	hsa-miR-20a-5p	Homo sapiens	F2R
MIRT220118	hsa-miR-20a-5p	Homo sapiens	CAV1
MIRT222673	hsa-miR-20a-5p	Homo sapiens	EIF4H
MIRT222908	hsa-miR-20a-5p	Homo sapiens	CROT
MIRT224747	hsa-miR-20a-5p	Homo sapiens	DPYSL2
MIRT224885	hsa-miR-20a-5p	Homo sapiens	MAK16
MIRT227326	hsa-miR-20a-5p	Homo sapiens	TRIM32
MIRT230965	hsa-miR-20a-5p	Homo sapiens	PRRG4
MIRT238172	hsa-miR-20a-5p	Homo sapiens	ANKRD33B
MIRT241294	hsa-miR-20a-5p	Homo sapiens	ZC3H12C
MIRT242196	hsa-miR-20a-5p	Homo sapiens	TTC9
MIRT242657	hsa-miR-20a-5p	Homo sapiens	SALL3
MIRT243778	hsa-miR-20a-5p	Homo sapiens	AFF1
MIRT244588	hsa-miR-20a-5p	Homo sapiens	HOOK3
MIRT244945	hsa-miR-20a-5p	Homo sapiens	PRRG1
MIRT246959	hsa-miR-20a-5p	Homo sapiens	TSKU
MIRT247079	hsa-miR-20a-5p	Homo sapiens	CEP57
MIRT248850	hsa-miR-20a-5p	Homo sapiens	SESN2
MIRT250444	hsa-miR-20a-5p	Homo sapiens	NFATC2IP
MIRT254248	hsa-miR-20a-5p	Homo sapiens	TRAPPC10
MIRT257279	hsa-miR-20a-5p	Homo sapiens	FOXC1
MIRT266853	hsa-miR-20a-5p	Homo sapiens	SLC25A44

MIRT280207	hsa-miR-20a-5p	Homo sapiens	EIF2B2
MIRT280991	hsa-miR-20a-5p	Homo sapiens	SPRED1
MIRT286945	hsa-miR-20a-5p	Homo sapiens	SOCS7
MIRT289571	hsa-miR-20a-5p	Homo sapiens	KDM6B
MIRT291931	hsa-miR-20a-5p	Homo sapiens	TPM4
MIRT293645	hsa-miR-20a-5p	Homo sapiens	PVR
MIRT296868	hsa-miR-20a-5p	Homo sapiens	REV1
MIRT299337	hsa-miR-20a-5p	Homo sapiens	CYBRD1
MIRT302434	hsa-miR-20a-5p	Homo sapiens	CLIP4
MIRT303488	hsa-miR-20a-5p	Homo sapiens	NAGK
MIRT322520	hsa-miR-20a-5p	Homo sapiens	HMBOX1
MIRT325510	hsa-miR-20a-5p	Homo sapiens	PTPDC1
MIRT363922	hsa-miR-20a-5p	Homo sapiens	UBE2V2
MIRT368882	hsa-miR-20a-5p	Homo sapiens	BCL2L2
MIRT397684	hsa-miR-20a-5p	Homo sapiens	ATXN7L3B
MIRT400046	hsa-miR-20a-5p	Homo sapiens	GRK3
MIRT437765	hsa-miR-20a-5p	Homo sapiens	PRKG1
MIRT437944	hsa-miR-20a-5p	Homo sapiens	RGS5
MIRT438054	hsa-miR-20a-5p	Homo sapiens	ETV1
MIRT438160	hsa-miR-20a-5p	Homo sapiens	EPAS1
MIRT438351	hsa-miR-20a-5p	Homo sapiens	FBXO31
MIRT438791	hsa-miR-20a-5p	Homo sapiens	TP53
MIRT438806	hsa-miR-20a-5p	Homo sapiens	DNMT1
MIRT438812	hsa-miR-20a-5p	Homo sapiens	PKD1
MIRT439181	hsa-miR-20a-5p	Homo sapiens	ZNF800
MIRT439185	hsa-miR-20a-5p	Homo sapiens	ZNF770
MIRT439192	hsa-miR-20a-5p	Homo sapiens	ZNF597
MIRT439211	hsa-miR-20a-5p	Homo sapiens	ZNF280C
MIRT439214	hsa-miR-20a-5p	Homo sapiens	ZNF280B
MIRT439225	hsa-miR-20a-5p	Homo sapiens	ZNF12
MIRT439256	hsa-miR-20a-5p	Homo sapiens	ZBTB7A
MIRT439257	hsa-miR-20a-5p	Homo sapiens	ZBTB6
MIRT439259	hsa-miR-20a-5p	Homo sapiens	ZBTB4
MIRT439269	hsa-miR-20a-5p	Homo sapiens	YOD1
MIRT439286	hsa-miR-20a-5p	Homo sapiens	WDR89
MIRT439291	hsa-miR-20a-5p	Homo sapiens	WDR1
MIRT439295	hsa-miR-20a-5p	Homo sapiens	VTI1A
MIRT439300	hsa-miR-20a-5p	Homo sapiens	VPS13C
MIRT439306	hsa-miR-20a-5p	Homo sapiens	VDAC1
MIRT439319	hsa-miR-20a-5p	Homo sapiens	UXS1
MIRT439327	hsa-miR-20a-5p	Homo sapiens	USP32
MIRT439332	hsa-miR-20a-5p	Homo sapiens	USP28
MIRT439337	hsa-miR-20a-5p	Homo sapiens	USP16
MIRT439349	hsa-miR-20a-5p	Homo sapiens	UBR5

MIRT439366	hsa-miR-20a-5p	Homo sapiens	UBC
MIRT439372	hsa-miR-20a-5p	Homo sapiens	TWF1
MIRT439399	hsa-miR-20a-5p	Homo sapiens	TOPORS
MIRT439410	hsa-miR-20a-5p	Homo sapiens	TNFRSF21
MIRT439416	hsa-miR-20a-5p	Homo sapiens	TMX3
MIRT439423	hsa-miR-20a-5p	Homo sapiens	TMEM67
MIRT439427	hsa-miR-20a-5p	Homo sapiens	TMEM64
MIRT439435	hsa-miR-20a-5p	Homo sapiens	TMEM167A
MIRT439439	hsa-miR-20a-5p	Homo sapiens	TMEM127
MIRT439440	hsa-miR-20a-5p	Homo sapiens	TMEM123
MIRT439460	hsa-miR-20a-5p	Homo sapiens	TGOLN2
MIRT439478	hsa-miR-20a-5p	Homo sapiens	TCF4
MIRT439489	hsa-miR-20a-5p	Homo sapiens	TADA2B
MIRT439512	hsa-miR-20a-5p	Homo sapiens	STX6
MIRT439521	hsa-miR-20a-5p	Homo sapiens	STK17B
MIRT439535	hsa-miR-20a-5p	Homo sapiens	SSX2IP
MIRT439537	hsa-miR-20a-5p	Homo sapiens	SSH2
MIRT439566	hsa-miR-20a-5p	Homo sapiens	SOD2
MIRT439590	hsa-miR-20a-5p	Homo sapiens	SLK
MIRT439594	hsa-miR-20a-5p	Homo sapiens	SLC4A7
MIRT439606	hsa-miR-20a-5p	Homo sapiens	SLC35F5
MIRT439620	hsa-miR-20a-5p	Homo sapiens	SLC16A9
MIRT439633	hsa-miR-20a-5p	Homo sapiens	SIKE1
MIRT439644	hsa-miR-20a-5p	Homo sapiens	SGTB
MIRT439648	hsa-miR-20a-5p	Homo sapiens	PEAK1
MIRT439654	hsa-miR-20a-5p	Homo sapiens	SRSF2
MIRT439680	hsa-miR-20a-5p	Homo sapiens	SENP1
MIRT439688	hsa-miR-20a-5p	Homo sapiens	SEC23A
MIRT439692	hsa-miR-20a-5p	Homo sapiens	SEC16A
MIRT439703	hsa-miR-20a-5p	Homo sapiens	SCAMP2
MIRT439711	hsa-miR-20a-5p	Homo sapiens	SAMD9L
MIRT439715	hsa-miR-20a-5p	Homo sapiens	SACS
MIRT439742	hsa-miR-20a-5p	Homo sapiens	RPL17
MIRT439758	hsa-miR-20a-5p	Homo sapiens	RNF216
MIRT439776	hsa-miR-20a-5p	Homo sapiens	RFXANK
MIRT439786	hsa-miR-20a-5p	Homo sapiens	REEP5
MIRT439809	hsa-miR-20a-5p	Homo sapiens	RBBP7
MIRT439821	hsa-miR-20a-5p	Homo sapiens	RAN
MIRT439832	hsa-miR-20a-5p	Homo sapiens	RABEP1
MIRT439837	hsa-miR-20a-5p	Homo sapiens	RAB30
MIRT439848	hsa-miR-20a-5p	Homo sapiens	RAB11FIP1
MIRT439853	hsa-miR-20a-5p	Homo sapiens	PURB
MIRT439863	hsa-miR-20a-5p	Homo sapiens	PTPN4
MIRT439874	hsa-miR-20a-5p	Homo sapiens	PTGES3

MIRT439875	hsa-miR-20a-5p	Homo sapiens	PTGER4
MIRT439905	hsa-miR-20a-5p	Homo sapiens	PPP6C
MIRT439910	hsa-miR-20a-5p	Homo sapiens	PPP3R1
MIRT439915	hsa-miR-20a-5p	Homo sapiens	PPP1R3B
MIRT439933	hsa-miR-20a-5p	Homo sapiens	POLQ
MIRT439940	hsa-miR-20a-5p	Homo sapiens	PNPLA4
MIRT439954	hsa-miR-20a-5p	Homo sapiens	PLAGL2
MIRT439959	hsa-miR-20a-5p	Homo sapiens	PKMYT1
MIRT439971	hsa-miR-20a-5p	Homo sapiens	PIP4K2A
MIRT439977	hsa-miR-20a-5p	Homo sapiens	PIGO
MIRT439991	hsa-miR-20a-5p	Homo sapiens	PGM2L1
MIRT440006	hsa-miR-20a-5p	Homo sapiens	PDZD11
MIRT440025	hsa-miR-20a-5p	Homo sapiens	PCMTD1
MIRT440046	hsa-miR-20a-5p	Homo sapiens	PANK3
MIRT440068	hsa-miR-20a-5p	Homo sapiens	NUP98
MIRT440072	hsa-miR-20a-5p	Homo sapiens	NUP35
MIRT440093	hsa-miR-20a-5p	Homo sapiens	NR2C2
MIRT440099	hsa-miR-20a-5p	Homo sapiens	NPAT
MIRT440112	hsa-miR-20a-5p	Homo sapiens	NIPA1
MIRT440138	hsa-miR-20a-5p	Homo sapiens	NCAPD2
MIRT440145	hsa-miR-20a-5p	Homo sapiens	NAA50
MIRT440158	hsa-miR-20a-5p	Homo sapiens	N4BP1
MIRT440170	hsa-miR-20a-5p	Homo sapiens	MXI1
MIRT440208	hsa-miR-20a-5p	Homo sapiens	MTF1
MIRT440233	hsa-miR-20a-5p	Homo sapiens	MKRN1
MIRT440235	hsa-miR-20a-5p	Homo sapiens	MKNK2
MIRT440260	hsa-miR-20a-5p	Homo sapiens	MECP2
MIRT440278	hsa-miR-20a-5p	Homo sapiens	MAPK1
MIRT440284	hsa-miR-20a-5p	Homo sapiens	MAP3K2
MIRT440286	hsa-miR-20a-5p	Homo sapiens	MAP3K14
MIRT440296	hsa-miR-20a-5p	Homo sapiens	M6PR
MIRT440318	hsa-miR-20a-5p	Homo sapiens	LPGAT1
MIRT440325	hsa-miR-20a-5p	Homo sapiens	LIMA1
MIRT440340	hsa-miR-20a-5p	Homo sapiens	LAPTM4A
MIRT440346	hsa-miR-20a-5p	Homo sapiens	LAMC1
MIRT440357	hsa-miR-20a-5p	Homo sapiens	KLHL28
MIRT440369	hsa-miR-20a-5p	Homo sapiens	KIF23
MIRT440386	hsa-miR-20a-5p	Homo sapiens	CCSER2
MIRT440388	hsa-miR-20a-5p	Homo sapiens	ATG14
MIRT440391	hsa-miR-20a-5p	Homo sapiens	EFCAB14
MIRT440410	hsa-miR-20a-5p	Homo sapiens	KATNAL1
MIRT440417	hsa-miR-20a-5p	Homo sapiens	ITPKB
MIRT440427	hsa-miR-20a-5p	Homo sapiens	ITCH
MIRT440430	hsa-miR-20a-5p	Homo sapiens	IQSEC1

MIRT440451	hsa-miR-20a-5p	Homo sapiens	INPP5F
MIRT440469	hsa-miR-20a-5p	Homo sapiens	IER3
MIRT440506	hsa-miR-20a-5p	Homo sapiens	HIF1AN
MIRT440509	hsa-miR-20a-5p	Homo sapiens	HAUS8
MIRT440537	hsa-miR-20a-5p	Homo sapiens	GPAM
MIRT440543	hsa-miR-20a-5p	Homo sapiens	GOLGA1
MIRT440557	hsa-miR-20a-5p	Homo sapiens	GNAS
MIRT440561	hsa-miR-20a-5p	Homo sapiens	GLO1
MIRT440573	hsa-miR-20a-5p	Homo sapiens	GIGYF1
MIRT440583	hsa-miR-20a-5p	Homo sapiens	GBP3
MIRT440590	hsa-miR-20a-5p	Homo sapiens	GAK
MIRT440593	hsa-miR-20a-5p	Homo sapiens	GABPB1
MIRT440595	hsa-miR-20a-5p	Homo sapiens	GABBR1
MIRT440601	hsa-miR-20a-5p	Homo sapiens	FYCO1
MIRT440609	hsa-miR-20a-5p	Homo sapiens	CMTR2
MIRT440633	hsa-miR-20a-5p	Homo sapiens	FMNL3
MIRT440646	hsa-miR-20a-5p	Homo sapiens	FEM1C
MIRT440653	hsa-miR-20a-5p	Homo sapiens	FBXO48
MIRT440657	hsa-miR-20a-5p	Homo sapiens	FBXO21
MIRT440660	hsa-miR-20a-5p	Homo sapiens	FBXO10
MIRT440662	hsa-miR-20a-5p	Homo sapiens	FBXL5
MIRT440674	hsa-miR-20a-5p	Homo sapiens	FAM83D
MIRT440686	hsa-miR-20a-5p	Homo sapiens	FAM126B
MIRT440693	hsa-miR-20a-5p	Homo sapiens	FAM102A
MIRT440699	hsa-miR-20a-5p	Homo sapiens	EZH1
MIRT440703	hsa-miR-20a-5p	Homo sapiens	ETF1
MIRT440712	hsa-miR-20a-5p	Homo sapiens	ERAP1
MIRT440720	hsa-miR-20a-5p	Homo sapiens	ENTPD7
MIRT440729	hsa-miR-20a-5p	Homo sapiens	EIF5A2
MIRT440752	hsa-miR-20a-5p	Homo sapiens	EEA1
MIRT440755	hsa-miR-20a-5p	Homo sapiens	E2F2
MIRT440759	hsa-miR-20a-5p	Homo sapiens	DYNC1LI2
MIRT440767	hsa-miR-20a-5p	Homo sapiens	DUSP18
MIRT440797	hsa-miR-20a-5p	Homo sapiens	DNAJC27
MIRT440826	hsa-miR-20a-5p	Homo sapiens	DENND5B
MIRT440840	hsa-miR-20a-5p	Homo sapiens	DDHD1
MIRT440857	hsa-miR-20a-5p	Homo sapiens	CTSS
MIRT440873	hsa-miR-20a-5p	Homo sapiens	CRTC3
MIRT440876	hsa-miR-20a-5p	Homo sapiens	CRK
MIRT440886	hsa-miR-20a-5p	Homo sapiens	CPOX
MIRT440918	hsa-miR-20a-5p	Homo sapiens	CNOT7
MIRT440933	hsa-miR-20a-5p	Homo sapiens	CLOCK
MIRT440941	hsa-miR-20a-5p	Homo sapiens	CIT
MIRT440943	hsa-miR-20a-5p	Homo sapiens	CHURC1

MIRT440953	hsa-miR-20a-5p	Homo sapiens	CFL2
MIRT440954	hsa-miR-20a-5p	Homo sapiens	CEP97
MIRT440976	hsa-miR-20a-5p	Homo sapiens	CD47
MIRT441010	hsa-miR-20a-5p	Homo sapiens	CAPRIN2
MIRT441030	hsa-miR-20a-5p	Homo sapiens	TMEM245
MIRT441031	hsa-miR-20a-5p	Homo sapiens	C9orf40
MIRT441033	hsa-miR-20a-5p	Homo sapiens	BMT2
MIRT441043	hsa-miR-20a-5p	Homo sapiens	TMEM267
MIRT441050	hsa-miR-20a-5p	Homo sapiens	PRR14L
MIRT441055	hsa-miR-20a-5p	Homo sapiens	SUCO
MIRT441057	hsa-miR-20a-5p	Homo sapiens	RSRP1
MIRT441073	hsa-miR-20a-5p	Homo sapiens	FAM210A
MIRT441082	hsa-miR-20a-5p	Homo sapiens	C14orf28
MIRT441084	hsa-miR-20a-5p	Homo sapiens	VCPKMT
MIRT441088	hsa-miR-20a-5p	Homo sapiens	EMSY
MIRT441094	hsa-miR-20a-5p	Homo sapiens	BTN3A3
MIRT441097	hsa-miR-20a-5p	Homo sapiens	BTBD7
MIRT441138	hsa-miR-20a-5p	Homo sapiens	ATXN1
MIRT441150	hsa-miR-20a-5p	Homo sapiens	ATP2B1
MIRT441164	hsa-miR-20a-5p	Homo sapiens	ATG2B
MIRT441167	hsa-miR-20a-5p	Homo sapiens	ATG2A
MIRT441187	hsa-miR-20a-5p	Homo sapiens	ARL1
MIRT441189	hsa-miR-20a-5p	Homo sapiens	ARID4B
MIRT441201	hsa-miR-20a-5p	Homo sapiens	ARHGAP1
MIRT441213	hsa-miR-20a-5p	Homo sapiens	ARAP2
MIRT441217	hsa-miR-20a-5p	Homo sapiens	AP1G1
MIRT441223	hsa-miR-20a-5p	Homo sapiens	ANKRD52
MIRT441227	hsa-miR-20a-5p	Homo sapiens	ANKRD13C
MIRT441232	hsa-miR-20a-5p	Homo sapiens	ANKFY1
MIRT441235	hsa-miR-20a-5p	Homo sapiens	ALDH9A1
MIRT441239	hsa-miR-20a-5p	Homo sapiens	AKTIP
MIRT441293	hsa-miR-20a-5p	Homo sapiens	ACBD5
MIRT441296	hsa-miR-20a-5p	Homo sapiens	ACAP2
MIRT441312	hsa-miR-20a-5p	Homo sapiens	ABCA1
MIRT441316	hsa-miR-20a-5p	Homo sapiens	AAK1
MIRT441880	hsa-miR-20a-5p	Homo sapiens	PFKFB2
MIRT442202	hsa-miR-20a-5p	Homo sapiens	VPS50
MIRT442551	hsa-miR-20a-5p	Homo sapiens	SLCO5A1
MIRT442768	hsa-miR-20a-5p	Homo sapiens	NRIP3
MIRT442802	hsa-miR-20a-5p	Homo sapiens	CEP170
MIRT443258	hsa-miR-20a-5p	Homo sapiens	A1CF
MIRT443712	hsa-miR-20a-5p	Homo sapiens	LLPH
MIRT444311	hsa-miR-20a-5p	Homo sapiens	SREK1IP1
MIRT444437	hsa-miR-20a-5p	Homo sapiens	EMC1

MIRT448315	hsa-miR-20a-5p	Homo sapiens	WNK3
MIRT448365	hsa-miR-20a-5p	Homo sapiens	TSR1
MIRT448645	hsa-miR-20a-5p	Homo sapiens	NPNT
MIRT448728	hsa-miR-20a-5p	Homo sapiens	ITGA2
MIRT449173	hsa-miR-20a-5p	Homo sapiens	SORCS2
MIRT450189	hsa-miR-20a-5p	Homo sapiens	TMEM9B
MIRT450915	hsa-miR-20a-5p	Homo sapiens	CADM2
MIRT450954	hsa-miR-20a-5p	Homo sapiens	ATAD2
MIRT458293	hsa-miR-20a-5p	Homo sapiens	FUT10
MIRT463554	hsa-miR-20a-5p	Homo sapiens	ZBTB5
MIRT464847	hsa-miR-20a-5p	Homo sapiens	RPS27A
MIRT465236	hsa-miR-20a-5p	Homo sapiens	TRIP10
MIRT466455	hsa-miR-20a-5p	Homo sapiens	TFAM
MIRT467495	hsa-miR-20a-5p	Homo sapiens	SMIM13
MIRT467895	hsa-miR-20a-5p	Homo sapiens	SLC22A23
MIRT468161	hsa-miR-20a-5p	Homo sapiens	SGPL1
MIRT468185	hsa-miR-20a-5p	Homo sapiens	SGMS1
MIRT469861	hsa-miR-20a-5p	Homo sapiens	PXK
MIRT470052	hsa-miR-20a-5p	Homo sapiens	PTGFRN
MIRT471004	hsa-miR-20a-5p	Homo sapiens	PITPNA
MIRT472168	hsa-miR-20a-5p	Homo sapiens	NIN
MIRT472288	hsa-miR-20a-5p	Homo sapiens	NFIB
MIRT473167	hsa-miR-20a-5p	Homo sapiens	MLLT1
MIRT474598	hsa-miR-20a-5p	Homo sapiens	KLF6
MIRT475410	hsa-miR-20a-5p	Homo sapiens	ICMT
MIRT475477	hsa-miR-20a-5p	Homo sapiens	HSPA8
MIRT476130	hsa-miR-20a-5p	Homo sapiens	GPR157
MIRT477116	hsa-miR-20a-5p	Homo sapiens	FAM160B1
MIRT477286	hsa-miR-20a-5p	Homo sapiens	ERGIC2
MIRT478722	hsa-miR-20a-5p	Homo sapiens	CSNK1A1
MIRT479040	hsa-miR-20a-5p	Homo sapiens	COIL
MIRT479064	hsa-miR-20a-5p	Homo sapiens	CNOT6L
MIRT479268	hsa-miR-20a-5p	Homo sapiens	CHSY1
MIRT480567	hsa-miR-20a-5p	Homo sapiens	BZW1
MIRT480676	hsa-miR-20a-5p	Homo sapiens	BSCL2
MIRT480786	hsa-miR-20a-5p	Homo sapiens	BMP2
MIRT480953	hsa-miR-20a-5p	Homo sapiens	BBX
MIRT481882	hsa-miR-20a-5p	Homo sapiens	ANKRD50
MIRT482138	hsa-miR-20a-5p	Homo sapiens	AKAP11
MIRT482477	hsa-miR-20a-5p	Homo sapiens	ADAR
MIRT484868	hsa-miR-20a-5p	Homo sapiens	ZNF70
MIRT484885	hsa-miR-20a-5p	Homo sapiens	ZNF652
MIRT484922	hsa-miR-20a-5p	Homo sapiens	ZFYVE26
MIRT485095	hsa-miR-20a-5p	Homo sapiens	SLC30A1

MIRT485193	hsa-miR-20a-5p	Homo sapiens	PTP4A1
MIRT485331	hsa-miR-20a-5p	Homo sapiens	MYO1D
MIRT485368	hsa-miR-20a-5p	Homo sapiens	MYLIP
MIRT485588	hsa-miR-20a-5p	Homo sapiens	FOXQ1
MIRT486029	hsa-miR-20a-5p	Homo sapiens	LPAR2
MIRT486757	hsa-miR-20a-5p	Homo sapiens	CNOT4
MIRT489607	hsa-miR-20a-5p	Homo sapiens	ZDHHC20
MIRT491660	hsa-miR-20a-5p	Homo sapiens	PDRG1
MIRT491807	hsa-miR-20a-5p	Homo sapiens	ZFYVE21
MIRT492011	hsa-miR-20a-5p	Homo sapiens	UGCG
MIRT492377	hsa-miR-20a-5p	Homo sapiens	SEMA7A
MIRT492786	hsa-miR-20a-5p	Homo sapiens	PDGFB
MIRT493619	hsa-miR-20a-5p	Homo sapiens	HMGB3
MIRT494427	hsa-miR-20a-5p	Homo sapiens	BTG2
MIRT496064	hsa-miR-20a-5p	Homo sapiens	MORC1
MIRT500724	hsa-miR-20a-5p	Homo sapiens	TRIM37
MIRT502008	hsa-miR-20a-5p	Homo sapiens	MAP7
MIRT503213	hsa-miR-20a-5p	Homo sapiens	ACER2
MIRT503562	hsa-miR-20a-5p	Homo sapiens	MDM2
MIRT503610	hsa-miR-20a-5p	Homo sapiens	ZNF780A
MIRT503829	hsa-miR-20a-5p	Homo sapiens	TMEM242
MIRT503967	hsa-miR-20a-5p	Homo sapiens	ZNF180
MIRT504566	hsa-miR-20a-5p	Homo sapiens	ZNF417
MIRT504641	hsa-miR-20a-5p	Homo sapiens	MFSD8
MIRT505060	hsa-miR-20a-5p	Homo sapiens	ZNF202
MIRT505866	hsa-miR-20a-5p	Homo sapiens	POLR1B
MIRT506282	hsa-miR-20a-5p	Homo sapiens	PDPK1
MIRT506378	hsa-miR-20a-5p	Homo sapiens	NUFIP2
MIRT506458	hsa-miR-20a-5p	Homo sapiens	NACC2
MIRT506547	hsa-miR-20a-5p	Homo sapiens	MORF4L1
MIRT506685	hsa-miR-20a-5p	Homo sapiens	LZIC
MIRT506887	hsa-miR-20a-5p	Homo sapiens	PCLAF
MIRT506991	hsa-miR-20a-5p	Homo sapiens	HNRNPR
MIRT507440	hsa-miR-20a-5p	Homo sapiens	ELK4
MIRT507947	hsa-miR-20a-5p	Homo sapiens	BTF3L4
MIRT508576	hsa-miR-20a-5p	Homo sapiens	CEP72
MIRT508745	hsa-miR-20a-5p	Homo sapiens	ZNF682
MIRT508837	hsa-miR-20a-5p	Homo sapiens	GPR155
MIRT509130	hsa-miR-20a-5p	Homo sapiens	BMP8B
MIRT509745	hsa-miR-20a-5p	Homo sapiens	EFCAB11
MIRT510029	hsa-miR-20a-5p	Homo sapiens	CRISPLD2
MIRT511267	hsa-miR-20a-5p	Homo sapiens	KLHL36
MIRT511556	hsa-miR-20a-5p	Homo sapiens	HMGB1
MIRT512322	hsa-miR-20a-5p	Homo sapiens	ACTR2

MIRT513709	hsa-miR-20a-5p	Homo sapiens	RBM20
MIRT513751	hsa-miR-20a-5p	Homo sapiens	PKNOX1
MIRT514101	hsa-miR-20a-5p	Homo sapiens	EPS15L1
MIRT514137	hsa-miR-20a-5p	Homo sapiens	SERF2
MIRT514320	hsa-miR-20a-5p	Homo sapiens	FXYD5
MIRT514998	hsa-miR-20a-5p	Homo sapiens	DNTTIP2
MIRT515205	hsa-miR-20a-5p	Homo sapiens	CRCP
MIRT515575	hsa-miR-20a-5p	Homo sapiens	TMEM134
MIRT516060	hsa-miR-20a-5p	Homo sapiens	MED18
MIRT516559	hsa-miR-20a-5p	Homo sapiens	MIXL1
MIRT516799	hsa-miR-20a-5p	Homo sapiens	CAVIN1
MIRT517021	hsa-miR-20a-5p	Homo sapiens	COX19
MIRT517187	hsa-miR-20a-5p	Homo sapiens	SLC28A1
MIRT517260	hsa-miR-20a-5p	Homo sapiens	PRIM1
MIRT518317	hsa-miR-20a-5p	Homo sapiens	ZNF514
MIRT518468	hsa-miR-20a-5p	Homo sapiens	KIF6
MIRT518699	hsa-miR-20a-5p	Homo sapiens	KCNMB1
MIRT518804	hsa-miR-20a-5p	Homo sapiens	MED16
MIRT518863	hsa-miR-20a-5p	Homo sapiens	NEK8
MIRT519548	hsa-miR-20a-5p	Homo sapiens	TMEM38A
MIRT520082	hsa-miR-20a-5p	Homo sapiens	YIPF4
MIRT520156	hsa-miR-20a-5p	Homo sapiens	WSB1
MIRT521010	hsa-miR-20a-5p	Homo sapiens	SOCS5
MIRT521304	hsa-miR-20a-5p	Homo sapiens	RRAGD
MIRT521549	hsa-miR-20a-5p	Homo sapiens	QSOX1
MIRT522172	hsa-miR-20a-5p	Homo sapiens	NR2F6
MIRT522505	hsa-miR-20a-5p	Homo sapiens	MFN1
MIRT523083	hsa-miR-20a-5p	Homo sapiens	HYPK
MIRT523654	hsa-miR-20a-5p	Homo sapiens	FOXK1
MIRT524083	hsa-miR-20a-5p	Homo sapiens	DNAJC10
MIRT524251	hsa-miR-20a-5p	Homo sapiens	DCTN6
MIRT524295	hsa-miR-20a-5p	Homo sapiens	CYCS
MIRT524457	hsa-miR-20a-5p	Homo sapiens	CNKSR3
MIRT524706	hsa-miR-20a-5p	Homo sapiens	BTG3
MIRT524984	hsa-miR-20a-5p	Homo sapiens	AGO3
MIRT525204	hsa-miR-20a-5p	Homo sapiens	ZNF93
MIRT525488	hsa-miR-20a-5p	Homo sapiens	TPK1
MIRT526639	hsa-miR-20a-5p	Homo sapiens	NME6
MIRT527206	hsa-miR-20a-5p	Homo sapiens	XIRP2
MIRT527474	hsa-miR-20a-5p	Homo sapiens	CLEC12B
MIRT528374	hsa-miR-20a-5p	Homo sapiens	ZMYM1
MIRT530003	hsa-miR-20a-5p	Homo sapiens	TNFAIP8L1
MIRT530591	hsa-miR-20a-5p	Homo sapiens	ABHD15
MIRT530989	hsa-miR-20a-5p	Homo sapiens	EXO5

MIRT531480	hsa-miR-20a-5p	Homo sapiens	TNFRSF10B
MIRT531779	hsa-miR-20a-5p	Homo sapiens	TXK
MIRT531839	hsa-miR-20a-5p	Homo sapiens	MTPAP
MIRT532053	hsa-miR-20a-5p	Homo sapiens	FHDC1
MIRT532618	hsa-miR-20a-5p	Homo sapiens	SPTLC2
MIRT532922	hsa-miR-20a-5p	Homo sapiens	ZNF385A
MIRT533881	hsa-miR-20a-5p	Homo sapiens	TBL1XR1
MIRT534008	hsa-miR-20a-5p	Homo sapiens	KMT5B
MIRT534573	hsa-miR-20a-5p	Homo sapiens	RPS6KA5
MIRT534631	hsa-miR-20a-5p	Homo sapiens	RNASEH1
MIRT535033	hsa-miR-20a-5p	Homo sapiens	PRKAR1A
MIRT536446	hsa-miR-20a-5p	Homo sapiens	KMT2B
MIRT536503	hsa-miR-20a-5p	Homo sapiens	TMEM131L
MIRT536542	hsa-miR-20a-5p	Homo sapiens	KCNJ8
MIRT537450	hsa-miR-20a-5p	Homo sapiens	FBXL7
MIRT537742	hsa-miR-20a-5p	Homo sapiens	ELAVL2
MIRT538040	hsa-miR-20a-5p	Homo sapiens	DNAJB6
MIRT538070	hsa-miR-20a-5p	Homo sapiens	DIAPH2
MIRT539427	hsa-miR-20a-5p	Homo sapiens	ADAT2
MIRT540278	hsa-miR-20a-5p	Homo sapiens	FAM89A
MIRT541093	hsa-miR-20a-5p	Homo sapiens	RLIM
MIRT542063	hsa-miR-20a-5p	Homo sapiens	SLC25A46
MIRT542145	hsa-miR-20a-5p	Homo sapiens	DIS3L
MIRT542271	hsa-miR-20a-5p	Homo sapiens	HSPA4L
MIRT543188	hsa-miR-20a-5p	Homo sapiens	FICD
MIRT543511	hsa-miR-20a-5p	Homo sapiens	PLS1
MIRT543583	hsa-miR-20a-5p	Homo sapiens	RPF2
MIRT544632	hsa-miR-20a-5p	Homo sapiens	CSDE1
MIRT546299	hsa-miR-20a-5p	Homo sapiens	TMEM200C
MIRT546694	hsa-miR-20a-5p	Homo sapiens	RORA
MIRT546827	hsa-miR-20a-5p	Homo sapiens	RAP2C
MIRT547085	hsa-miR-20a-5p	Homo sapiens	PLRG1
MIRT548324	hsa-miR-20a-5p	Homo sapiens	EPHA4
MIRT548396	hsa-miR-20a-5p	Homo sapiens	ENPP5
MIRT548822	hsa-miR-20a-5p	Homo sapiens	CLIC4
MIRT548867	hsa-miR-20a-5p	Homo sapiens	CERCAM
MIRT549113	hsa-miR-20a-5p	Homo sapiens	C16orf70
MIRT550097	hsa-miR-20a-5p	Homo sapiens	TRAPPC2
MIRT550308	hsa-miR-20a-5p	Homo sapiens	ZNF681
MIRT551127	hsa-miR-20a-5p	Homo sapiens	ZNF107
MIRT551748	hsa-miR-20a-5p	Homo sapiens	FMNL2
MIRT552208	hsa-miR-20a-5p	Homo sapiens	F2RL3
MIRT552689	hsa-miR-20a-5p	Homo sapiens	YWHAZ
MIRT552869	hsa-miR-20a-5p	Homo sapiens	WIPF2

MIRT552899	hsa-miR-20a-5p	Homo sapiens	WASL
MIRT553034	hsa-miR-20a-5p	Homo sapiens	USP48
MIRT553579	hsa-miR-20a-5p	Homo sapiens	TMEM100
MIRT553705	hsa-miR-20a-5p	Homo sapiens	TCF7L2
MIRT554416	hsa-miR-20a-5p	Homo sapiens	SCD
MIRT554485	hsa-miR-20a-5p	Homo sapiens	SAMD12
MIRT554558	hsa-miR-20a-5p	Homo sapiens	RRN3
MIRT554751	hsa-miR-20a-5p	Homo sapiens	RHOC
MIRT555468	hsa-miR-20a-5p	Homo sapiens	POLR3A
MIRT556188	hsa-miR-20a-5p	Homo sapiens	MCC
MIRT556596	hsa-miR-20a-5p	Homo sapiens	LEPROT
MIRT556908	hsa-miR-20a-5p	Homo sapiens	ISOC1
MIRT557596	hsa-miR-20a-5p	Homo sapiens	GNPTAB
MIRT557768	hsa-miR-20a-5p	Homo sapiens	FRS2
MIRT557892	hsa-miR-20a-5p	Homo sapiens	FEM1B
MIRT558341	hsa-miR-20a-5p	Homo sapiens	DNAJC28
MIRT558939	hsa-miR-20a-5p	Homo sapiens	CBX1
MIRT559442	hsa-miR-20a-5p	Homo sapiens	ARSJ
MIRT561247	hsa-miR-20a-5p	Homo sapiens	ZNF354B
MIRT561650	hsa-miR-20a-5p	Homo sapiens	RUNX3
MIRT562219	hsa-miR-20a-5p	Homo sapiens	HMGB2
MIRT562567	hsa-miR-20a-5p	Homo sapiens	CCDC71L
MIRT562969	hsa-miR-20a-5p	Homo sapiens	LRPAP1
MIRT563383	hsa-miR-20a-5p	Homo sapiens	DSPP
MIRT564687	hsa-miR-20a-5p	Homo sapiens	ZNF35
MIRT565704	hsa-miR-20a-5p	Homo sapiens	SESN3
MIRT566145	hsa-miR-20a-5p	Homo sapiens	RACGAP1
MIRT566573	hsa-miR-20a-5p	Homo sapiens	OTUD4
MIRT566882	hsa-miR-20a-5p	Homo sapiens	LRP12
MIRT567059	hsa-miR-20a-5p	Homo sapiens	KCNB1
MIRT567117	hsa-miR-20a-5p	Homo sapiens	ITGB1
MIRT567689	hsa-miR-20a-5p	Homo sapiens	EIF4A2
MIRT567850	hsa-miR-20a-5p	Homo sapiens	DCAF8
MIRT567996	hsa-miR-20a-5p	Homo sapiens	COX6B1
MIRT568184	hsa-miR-20a-5p	Homo sapiens	CCDC6
MIRT568274	hsa-miR-20a-5p	Homo sapiens	BICD2
MIRT571019	hsa-miR-20a-5p	Homo sapiens	CKAP2
MIRT571682	hsa-miR-20a-5p	Homo sapiens	RRAS2
MIRT571698	hsa-miR-20a-5p	Homo sapiens	RPRD2
MIRT571728	hsa-miR-20a-5p	Homo sapiens	RPL17-C18orf32
MIRT571863	hsa-miR-20a-5p	Homo sapiens	NKIRAS1
MIRT572213	hsa-miR-20a-5p	Homo sapiens	C18orf32
MIRT572680	hsa-miR-20a-5p	Homo sapiens	AGMAT
MIRT573920	hsa-miR-20a-5p	Homo sapiens	SNAP47

MIRT608372	hsa-miR-20a-5p	Homo sapiens	PIWIL2
MIRT608752	hsa-miR-20a-5p	Homo sapiens	MYH9
MIRT608978	hsa-miR-20a-5p	Homo sapiens	PRKCB
MIRT611003	hsa-miR-20a-5p	Homo sapiens	BRI3BP
MIRT611840	hsa-miR-20a-5p	Homo sapiens	FEM1A
MIRT612615	hsa-miR-20a-5p	Homo sapiens	RANGAP1
MIRT614267	hsa-miR-20a-5p	Homo sapiens	WDR53
MIRT615180	hsa-miR-20a-5p	Homo sapiens	SPIB
MIRT615451	hsa-miR-20a-5p	Homo sapiens	FAXC
MIRT616176	hsa-miR-20a-5p	Homo sapiens	ELOC
MIRT619845	hsa-miR-20a-5p	Homo sapiens	POLM
MIRT620981	hsa-miR-20a-5p	Homo sapiens	TM4SF5
MIRT624428	hsa-miR-20a-5p	Homo sapiens	CBX8
MIRT626042	hsa-miR-20a-5p	Homo sapiens	ATAT1
MIRT626225	hsa-miR-20a-5p	Homo sapiens	PNRC1
MIRT628569	hsa-miR-20a-5p	Homo sapiens	MELK
MIRT633126	hsa-miR-20a-5p	Homo sapiens	CBX5
MIRT634102	hsa-miR-20a-5p	Homo sapiens	APOH
MIRT634459	hsa-miR-20a-5p	Homo sapiens	PAK6
MIRT634650	hsa-miR-20a-5p	Homo sapiens	HIP1
MIRT634959	hsa-miR-20a-5p	Homo sapiens	GTF2H2C
MIRT640014	hsa-miR-20a-5p	Homo sapiens	OSTM1
MIRT641710	hsa-miR-20a-5p	Homo sapiens	SPCS1
MIRT645219	hsa-miR-20a-5p	Homo sapiens	POLR3F
MIRT662411	hsa-miR-20a-5p	Homo sapiens	ICA1L
MIRT664232	hsa-miR-20a-5p	Homo sapiens	LSM3
MIRT664398	hsa-miR-20a-5p	Homo sapiens	CYB5A
MIRT664798	hsa-miR-20a-5p	Homo sapiens	LIAS
MIRT673138	hsa-miR-20a-5p	Homo sapiens	MFSD2A
MIRT675843	hsa-miR-20a-5p	Homo sapiens	DHODH
MIRT677159	hsa-miR-20a-5p	Homo sapiens	DEGS1
MIRT677265	hsa-miR-20a-5p	Homo sapiens	C15orf40
MIRT678752	hsa-miR-20a-5p	Homo sapiens	SRCAP
MIRT680378	hsa-miR-20a-5p	Homo sapiens	GATAD1
MIRT680705	hsa-miR-20a-5p	Homo sapiens	ZNF785
MIRT680783	hsa-miR-20a-5p	Homo sapiens	WDR73
MIRT681415	hsa-miR-20a-5p	Homo sapiens	RMND1
MIRT681709	hsa-miR-20a-5p	Homo sapiens	ABI2
MIRT681845	hsa-miR-20a-5p	Homo sapiens	N4BP2L2
MIRT682336	hsa-miR-20a-5p	Homo sapiens	RAB42
MIRT683334	hsa-miR-20a-5p	Homo sapiens	FAAP24
MIRT683404	hsa-miR-20a-5p	Homo sapiens	ESR2
MIRT683508	hsa-miR-20a-5p	Homo sapiens	ZNF7
MIRT683540	hsa-miR-20a-5p	Homo sapiens	C11orf54

MIRT683891	hsa-miR-20a-5p	Homo sapiens	OCIAD1
MIRT683964	hsa-miR-20a-5p	Homo sapiens	MYLK3
MIRT683993	hsa-miR-20a-5p	Homo sapiens	QRFPR
MIRT684096	hsa-miR-20a-5p	Homo sapiens	TLR7
MIRT684149	hsa-miR-20a-5p	Homo sapiens	CEP104
MIRT684374	hsa-miR-20a-5p	Homo sapiens	BCAS4
MIRT684590	hsa-miR-20a-5p	Homo sapiens	ORAI2
MIRT684633	hsa-miR-20a-5p	Homo sapiens	GTF2IRD2B
MIRT684803	hsa-miR-20a-5p	Homo sapiens	MYO1F
MIRT684935	hsa-miR-20a-5p	Homo sapiens	CD28
MIRT685211	hsa-miR-20a-5p	Homo sapiens	DCTN5
MIRT685367	hsa-miR-20a-5p	Homo sapiens	CCL5
MIRT685535	hsa-miR-20a-5p	Homo sapiens	MSH3
MIRT685594	hsa-miR-20a-5p	Homo sapiens	KCNK6
MIRT685725	hsa-miR-20a-5p	Homo sapiens	BHMT2
MIRT685758	hsa-miR-20a-5p	Homo sapiens	C12orf65
MIRT685799	hsa-miR-20a-5p	Homo sapiens	ZNF426
MIRT685971	hsa-miR-20a-5p	Homo sapiens	PTGIS
MIRT686122	hsa-miR-20a-5p	Homo sapiens	TNIP3
MIRT686172	hsa-miR-20a-5p	Homo sapiens	HS3ST1
MIRT686299	hsa-miR-20a-5p	Homo sapiens	WWC1
MIRT686337	hsa-miR-20a-5p	Homo sapiens	VPS53
MIRT686504	hsa-miR-20a-5p	Homo sapiens	TRIOBP
MIRT686543	hsa-miR-20a-5p	Homo sapiens	TRAF3IP2
MIRT686598	hsa-miR-20a-5p	Homo sapiens	TMOD3
MIRT686843	hsa-miR-20a-5p	Homo sapiens	SLC7A11
MIRT686896	hsa-miR-20a-5p	Homo sapiens	SLC1A5
MIRT686996	hsa-miR-20a-5p	Homo sapiens	SERINC1
MIRT687060	hsa-miR-20a-5p	Homo sapiens	RNF115
MIRT687095	hsa-miR-20a-5p	Homo sapiens	RABGAP1L
MIRT687270	hsa-miR-20a-5p	Homo sapiens	PDHB
MIRT687666	hsa-miR-20a-5p	Homo sapiens	LRIF1
MIRT687874	hsa-miR-20a-5p	Homo sapiens	ISCA2
MIRT687999	hsa-miR-20a-5p	Homo sapiens	GTF2IRD2
MIRT688139	hsa-miR-20a-5p	Homo sapiens	GEMIN8
MIRT688233	hsa-miR-20a-5p	Homo sapiens	FKBP14
MIRT688473	hsa-miR-20a-5p	Homo sapiens	DNAJB4
MIRT688522	hsa-miR-20a-5p	Homo sapiens	DDI2
MIRT688682	hsa-miR-20a-5p	Homo sapiens	CPT1A
MIRT688716	hsa-miR-20a-5p	Homo sapiens	CPS1
MIRT688846	hsa-miR-20a-5p	Homo sapiens	CAPZA2
MIRT689128	hsa-miR-20a-5p	Homo sapiens	ZBTB25
MIRT689190	hsa-miR-20a-5p	Homo sapiens	ZNF665
MIRT689815	hsa-miR-20a-5p	Homo sapiens	GTF2H3

MIRT690755	hsa-miR-20a-5p	Homo sapiens	IRAK4
MIRT691000	hsa-miR-20a-5p	Homo sapiens	ZNF578
MIRT691092	hsa-miR-20a-5p	Homo sapiens	NUGGC
MIRT691349	hsa-miR-20a-5p	Homo sapiens	KIAA1841
MIRT691512	hsa-miR-20a-5p	Homo sapiens	FOXRED2
MIRT691594	hsa-miR-20a-5p	Homo sapiens	CCDC125
MIRT691630	hsa-miR-20a-5p	Homo sapiens	IPP
MIRT692090	hsa-miR-20a-5p	Homo sapiens	ACOT9
MIRT692127	hsa-miR-20a-5p	Homo sapiens	CXorf38
MIRT692336	hsa-miR-20a-5p	Homo sapiens	RFK
MIRT692398	hsa-miR-20a-5p	Homo sapiens	LY6G5B
MIRT692459	hsa-miR-20a-5p	Homo sapiens	METTL8
MIRT692562	hsa-miR-20a-5p	Homo sapiens	PARD3
MIRT692835	hsa-miR-20a-5p	Homo sapiens	C1orf50
MIRT692897	hsa-miR-20a-5p	Homo sapiens	RBM41
MIRT693157	hsa-miR-20a-5p	Homo sapiens	THEM4
MIRT693368	hsa-miR-20a-5p	Homo sapiens	RNF34
MIRT694129	hsa-miR-20a-5p	Homo sapiens	ZNF446
MIRT694213	hsa-miR-20a-5p	Homo sapiens	ZNF347
MIRT694679	hsa-miR-20a-5p	Homo sapiens	C14orf119
MIRT694833	hsa-miR-20a-5p	Homo sapiens	STX4
MIRT694955	hsa-miR-20a-5p	Homo sapiens	ANKS4B
MIRT695199	hsa-miR-20a-5p	Homo sapiens	SLC25A33
MIRT695681	hsa-miR-20a-5p	Homo sapiens	MAN2B2
MIRT695944	hsa-miR-20a-5p	Homo sapiens	ZNF174
MIRT696202	hsa-miR-20a-5p	Homo sapiens	GNB5
MIRT696463	hsa-miR-20a-5p	Homo sapiens	SUGP1
MIRT696879	hsa-miR-20a-5p	Homo sapiens	UBOX5
MIRT696926	hsa-miR-20a-5p	Homo sapiens	CCDC198
MIRT697265	hsa-miR-20a-5p	Homo sapiens	ZYG11A
MIRT697410	hsa-miR-20a-5p	Homo sapiens	ZMAT3
MIRT698004	hsa-miR-20a-5p	Homo sapiens	TSPAN6
MIRT699290	hsa-miR-20a-5p	Homo sapiens	SLC6A4
MIRT699653	hsa-miR-20a-5p	Homo sapiens	SH3BP5
MIRT699715	hsa-miR-20a-5p	Homo sapiens	SF3B3
MIRT700064	hsa-miR-20a-5p	Homo sapiens	RPL14
MIRT700122	hsa-miR-20a-5p	Homo sapiens	RNF19B
MIRT701314	hsa-miR-20a-5p	Homo sapiens	NUDT3
MIRT701596	hsa-miR-20a-5p	Homo sapiens	MYPN
MIRT702396	hsa-miR-20a-5p	Homo sapiens	KLF10
MIRT702545	hsa-miR-20a-5p	Homo sapiens	KCND3
MIRT703111	hsa-miR-20a-5p	Homo sapiens	GPRIN3
MIRT704130	hsa-miR-20a-5p	Homo sapiens	DRAXIN
MIRT704161	hsa-miR-20a-5p	Homo sapiens	DNAL1

MIRT704217	hsa-miR-20a-5p	Homo sapiens	LDHD
MIRT704783	hsa-miR-20a-5p	Homo sapiens	CDKN2AIPNL
MIRT705104	hsa-miR-20a-5p	Homo sapiens	ABHD18
MIRT705369	hsa-miR-20a-5p	Homo sapiens	ATP1B3
MIRT706125	hsa-miR-20a-5p	Homo sapiens	ENTPD4
MIRT706295	hsa-miR-20a-5p	Homo sapiens	SLC35F6
MIRT706331	hsa-miR-20a-5p	Homo sapiens	CCDC30
MIRT706423	hsa-miR-20a-5p	Homo sapiens	HAS2
MIRT706534	hsa-miR-20a-5p	Homo sapiens	MTMR9
MIRT707608	hsa-miR-20a-5p	Homo sapiens	PCNX2
MIRT708390	hsa-miR-20a-5p	Homo sapiens	CDIPT
MIRT708469	hsa-miR-20a-5p	Homo sapiens	MAPKAPK5
MIRT709092	hsa-miR-20a-5p	Homo sapiens	FAHD1
MIRT709557	hsa-miR-20a-5p	Homo sapiens	ZBED1
MIRT710425	hsa-miR-20a-5p	Homo sapiens	YTHDC1
MIRT711789	hsa-miR-20a-5p	Homo sapiens	RFXAP
MIRT713354	hsa-miR-20a-5p	Homo sapiens	KLRD1
MIRT714326	hsa-miR-20a-5p	Homo sapiens	ZNF454
MIRT716560	hsa-miR-20a-5p	Homo sapiens	GOLGA2
MIRT719091	hsa-miR-20a-5p	Homo sapiens	ACOX1
MIRT725227	hsa-miR-20a-5p	Homo sapiens	PEA15
MIRT731268	hsa-miR-20a-5p	Homo sapiens	RB1CC1
MIRT731837	hsa-miR-20a-5p	Homo sapiens	NFKBIB
MIRT732249	hsa-miR-20a-5p	Homo sapiens	KIF26B
MIRT732435	hsa-miR-20a-5p	Homo sapiens	TIMP2
MIRT732671	hsa-miR-20a-5p	Homo sapiens	NTN4
MIRT734034	hsa-miR-20a-5p	Homo sapiens	TGFBR1
MIRT734853	hsa-miR-20a-5p	Homo sapiens	PTPRO
MIRT734854	hsa-miR-20a-5p	Homo sapiens	PPP2R2A
MIRT735333	hsa-miR-20a-5p	Homo sapiens	DAPK3
MIRT738870	hsa-miR-20a-5p	Homo sapiens	DCBLD2
MIRT738871	hsa-miR-20a-5p	Homo sapiens	EREG
MIRT738872	hsa-miR-20a-5p	Homo sapiens	GPR183
MIRT738874	hsa-miR-20a-5p	Homo sapiens	NBL1
MIRT738875	hsa-miR-20a-5p	Homo sapiens	PLEKHM1
MIRT738876	hsa-miR-20a-5p	Homo sapiens	RAB3IP
MIRT738877	hsa-miR-20a-5p	Homo sapiens	SYNJ2BP
MIRT763757	hsa-miR-20a-5p	Homo sapiens	DPP9
MIRT763758	hsa-miR-20a-5p	Homo sapiens	DSTYK
MIRT763759	hsa-miR-20a-5p	Homo sapiens	ISY1
MIRT763760	hsa-miR-20a-5p	Homo sapiens	KPNA6
MIRT763761	hsa-miR-20a-5p	Homo sapiens	NR3C1
MIRT763762	hsa-miR-20a-5p	Homo sapiens	PMAIP1
MIRT763763	hsa-miR-20a-5p	Homo sapiens	SESN1

MIRT763764	hsa-miR-20a-5p	Homo sapiens	SLC12A6
MIRT763765	hsa-miR-20a-5p	Homo sapiens	TRIM65
MIRT784171	hsa-miR-20a-5p	Homo sapiens	ARMT1
MIRT784172	hsa-miR-20a-5p	Homo sapiens	C17orf75
MIRT784173	hsa-miR-20a-5p	Homo sapiens	FAM241A
MIRT784174	hsa-miR-20a-5p	Homo sapiens	LRRC58
MIRT784175	hsa-miR-20a-5p	Homo sapiens	MPPE1
MIRT784176	hsa-miR-20a-5p	Homo sapiens	MRPS10
MIRT784178	hsa-miR-20a-5p	Homo sapiens	SON
MIRT784179	hsa-miR-20a-5p	Homo sapiens	SP2
MIRT784180	hsa-miR-20a-5p	Homo sapiens	WDR92
MIRT784181	hsa-miR-20a-5p	Homo sapiens	ZNF786

Table S15. The identified 31 miRNA-mRNA pairs with correlation coefficients (R value) meeting the criteria of FDR< 0.05 and R < -0.3 among control group, COCA1, COCA2 and COCA3.

miRNA	genename	R	FDR.value
miR-24-3p	NET1	-0.30116	0.001319
miR-20a-5p	AP3D1	-0.30182	0.001285
miR-20a-5p	CCND2	-0.30201	0.001275
miR-20a-5p	RPL31	-0.30318	0.001218
miR-24-3p	RAP2B	-0.30528	0.001122
miR-20a-5p	IQSEC1	-0.31293	0.000826
miR-20a-5p	CSDE1	-0.3149	0.000762
miR-20a-5p	ARCN1	-0.32148	0.000581
miR-652-3p	RPL32	-0.32417	0.000519
miR-24-3p	RPS16	-0.32686	0.000463

miR-20a-5p	PCBP2	-0.32718	0.000457
miR-20a-5p	TUBB	-0.33096	0.000388
miR-20a-5p	PTGES3	-0.33891	0.000274
miR-20a-5p	CCND1	-0.34811	0.000181
miR-20a-5p	PRKCB	-0.35315	0.000144
miR-20a-5p	YTHDC1	-0.35499	0.000132
miR-20a-5p	RPL17	-0.35542	0.000129
miR-20a-5p	EPAS1	-0.35581	0.000127
miR-20a-5p	ARPC2	-0.37653	4.64E-05
miR-20a-5p	DYNC1LI2	-0.38372	3.22E-05
miR-20a-5p	DPYSL2	-0.39261	2.03E-05
miR-20a-5p	RPS27A	-0.39805	1.52E-05
miR-24-3p	S100A8	-0.41063	7.59E-06
miR-24-3p	RPL37A	-0.42362	3.61E-06
miR-20a-5p	RPL18A	-0.42562	3.21E-06
miR-20a-5p	YBX1	-0.45112	6.73E-07
miR-20a-5p	RPS10	-0.45385	5.65E-07
miR-24-3p	RPS7	-0.47608	1.28E-07
miR-20a-5p	PNRC1	-0.49423	3.53E-08
miR-24-3p	EEF1A1	-0.49476	3.40E-08
miR-20a-5p	RPL21	-0.49859	2.56E-08

Supplementary figures

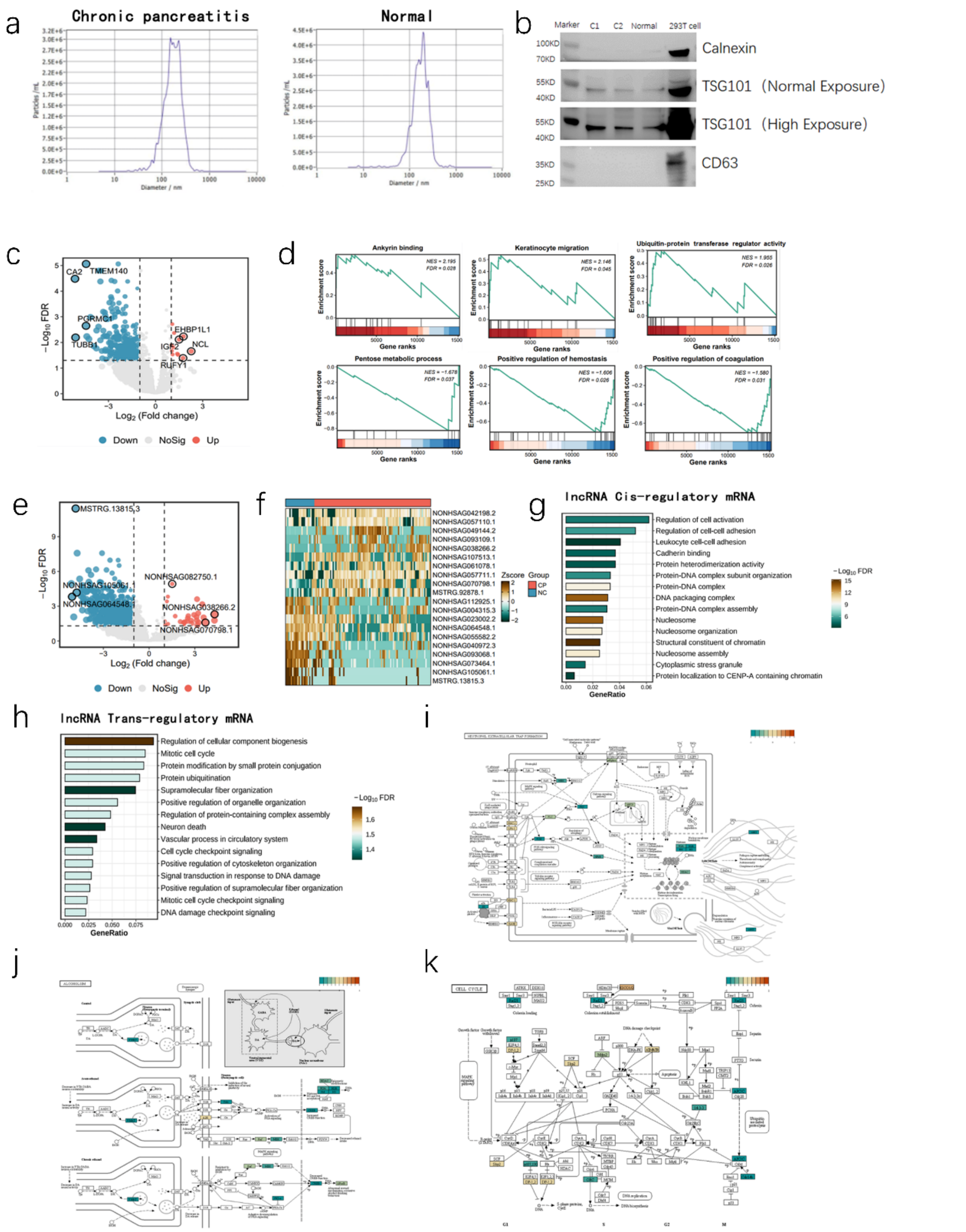

Figure S1. (a) Left: Representative particle size analysis imagine of EV from CP patients. Right: Representative particle size analysis imagine of EV from CP patients. (b) The protein expression of EV markers in two CP EVs(C1 and C2), one EV sample from healthy donor(Normal) and 293 T cell as control sample. (c) The volcano plot of differential expressed mRNAs. (d) Top 6 enriched pathway based on differential expressed mRNAs identified through GSEA. (e) The volcano plot of differential expressed lncRNA. (f) Top 10 differential expressed lncRNA between NC group and CP group. (g) The upregulated enrichment GO term of putative cis-regulatory mRNA

of differential expressed lncRNAs. (FDR<0.05) (h) The upregulated enrichment GO term of putative trans-regulatory mRNA of differential expressed lncRNAs. (FDR<0.05) (i) The expression of putative cis-regulatory mRNA and trans-regulatory mRNA of differential expressed lncRNAs in neutrophil extracellular trap formation pathway. (j) The expression of putative cis-regulatory mRNA and trans-regulatory mRNA of differential expressed lncRNAs in alcoholism pathway. (k) The expression of putative cis-regulatory mRNA and trans-regulatory mRNA of differential expressed lncRNAs in cell cycle pathway.

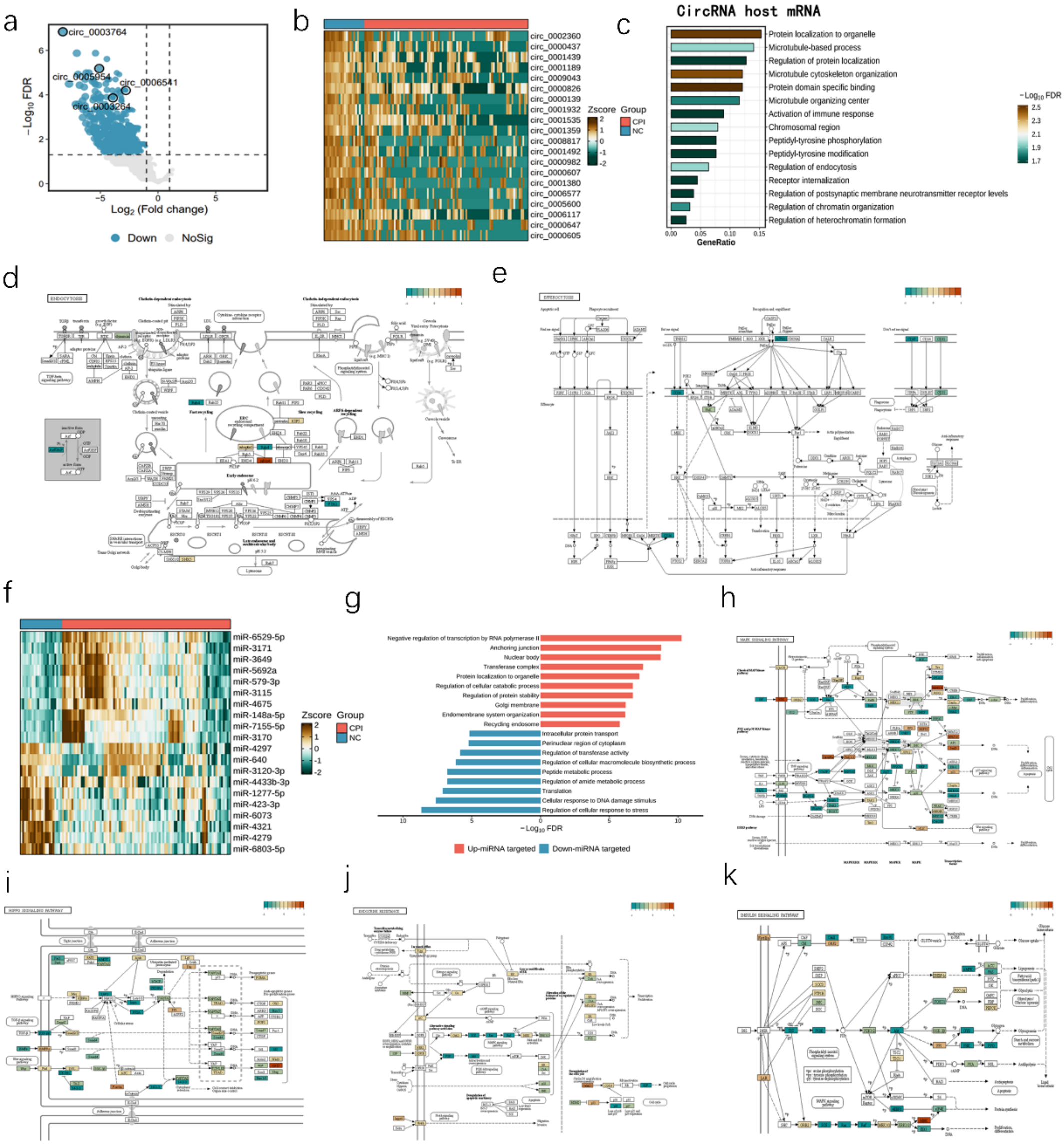

Figure S2. (a) The volcano plot of differential expressed circRNA. (b) Top 10 differential expressed circRNA between NC group and CP group. (c) The upregulated enrichment GO term of host genes of differential expressed circRNAs. (FDR<0.05) (d) The expression of host genes of differential expressed circRNAs in endocytosis pathway. (e) The expression of host genes of differential expressed circRNAs in efferocytosis pathway. (f) Top 10 differential expressed miRNA between NC group and CP group. (g) Top 10 enrichment GO term based on the differential expressed miRNA (upregulated or downregulated) targeted genes between NC group and CP group. (h) The expression of differential expressed miRNA (upregulated or downregulated) targeted genes in MAPK pathway. (i) The expression of differential expressed miRNA (upregulated or downregulated) targeted genes in HIPPO pathway. (j) The expression of differential expressed miRNA (upregulated or downregulated) targeted genes in endocrine resistance pathway. (k) The expression of differential

expressed miRNA (upregulated or downregulated) targeted genes in insulin signaling pathway.

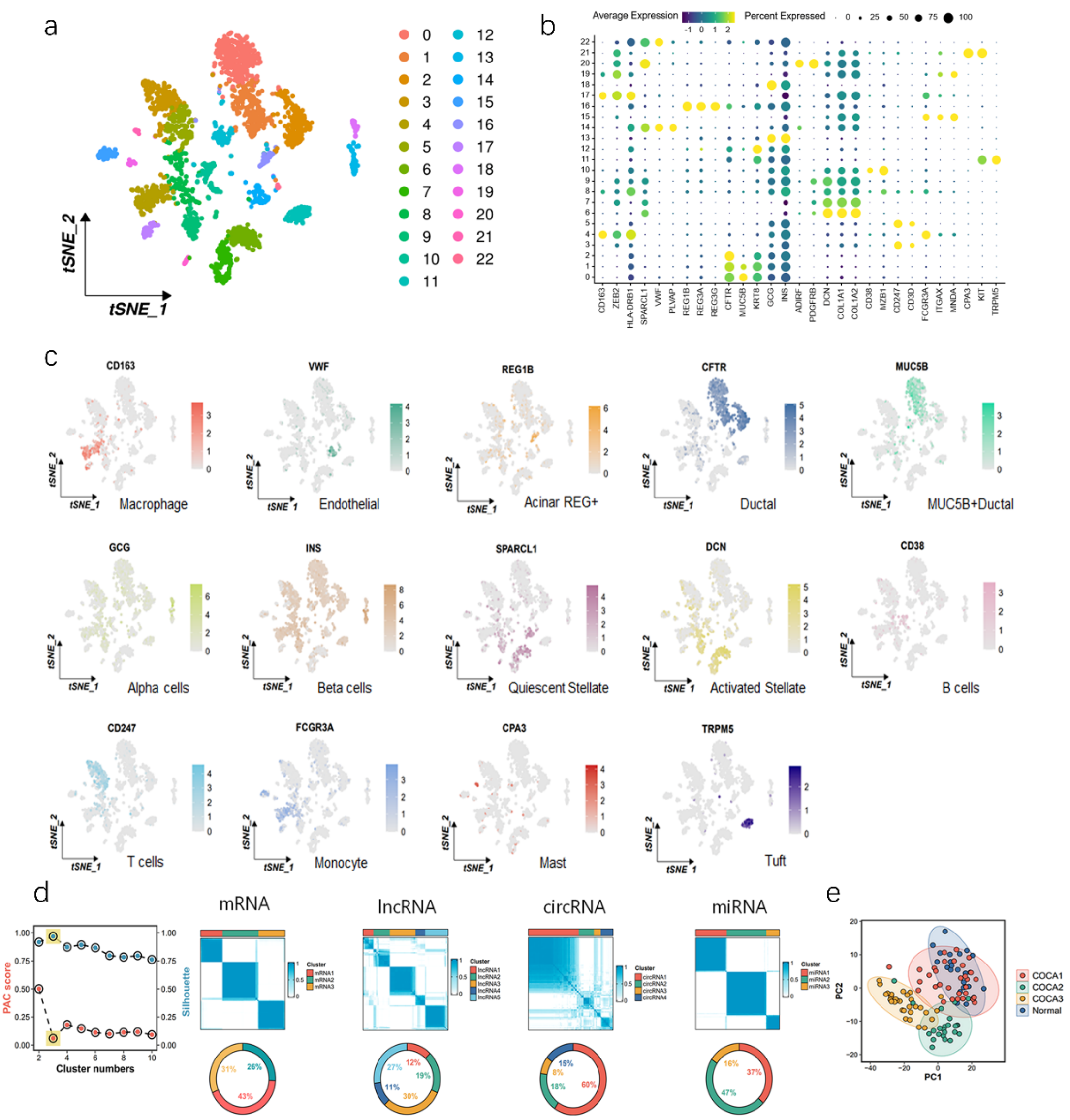

Figure S3. (a) UMAP of identified clusters in single-cell data. (b) Dot plot shows the expression of identified cell markers in each cluster. (c) The expression of some pivotal markers in single-cell data. (d) Left: The PAC (Proportion of ambiguous clustering) score among clusters. Upper Right: expression matrixes of mRNA, lncRNA, circRNA and miRNA when PAC score=3. Lower Right: The percentage of each cluster in mRNA, lncRNA, circRNA and miRNA when PAC score=3. (e) Principal Component Analysis (PCA) based on expression of mRNA from these two signatures in EV sequencing data shows the distribution of each subtype.

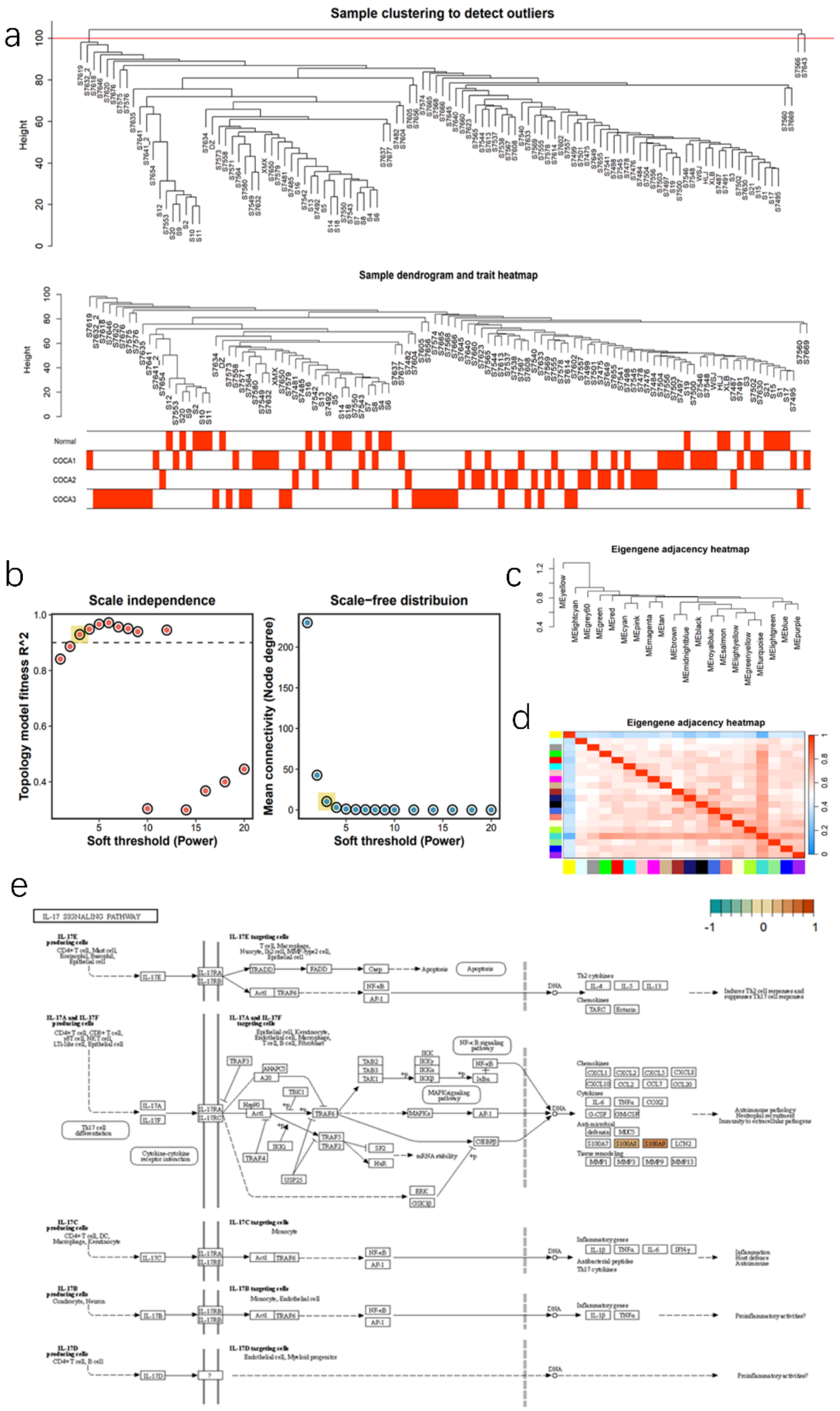

Figure S4. (a) Sample dendrogram and trait heatmap of all samples. (b) The distribution scaled by Topology model fitness and mean connectivity. (c) The dendrogram of eigengene adjacency heatmap all samples. (d) The eigengene adjacency heatmap of all samples. (e) The expression of differential expressed proteins (upregulated or downregulated) in IL-17 pathway.

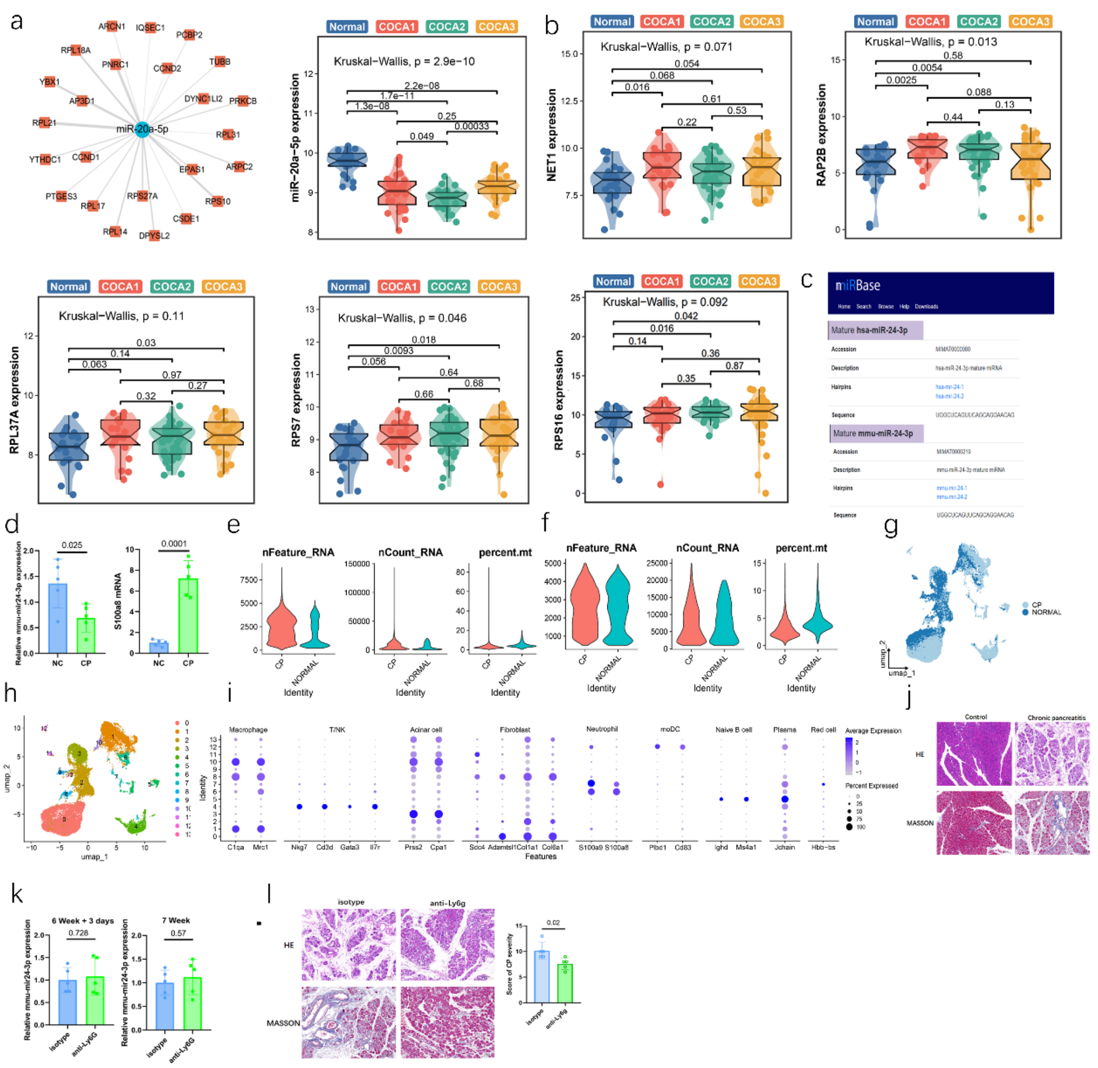

Figure S5. (a) Left: The screened miRNA–mRNA interaction network. Right: The expression of miR-20a-5 in each group. (b) The expression of 5 putative miR-24-3p binding mRNA, including NET1, RAP2B, RPL37A, RPS7 and RPS16 in each group. (c) The sequencing of hsa-miR-24-3p and mmu-miR-24-3p in miRbase. (d) Left: the expression of miR-24-3p in extracted exosome from plasma of CP mice and normal

group mice. Right: the mRNA expression of S100a8 in extracted exosome from plasma of CP mice and normal group mice. (e) The RNA feature counts, RNA counts and mitochondrial gene ratio before filtering. (f) The RNA feature counts, RNA counts and mitochondrial gene ratio after filtering. (g) The UMAP plot of all cells between CP group and NC group. (h) UMAP of identified clusters in single-cell data. (i) Dot plot shows the expression of identified cell markers in each cluster. (j) The representative HE and MASSON imagine of pancreas from chronic pancreatitis modeling mouse and healthy control mouse. (k) Exosomal mmu-miR-24-3p expression profiles in CP mouse models treated with neutrophil-depleting anti-Ly6G antibod versus isotype control antibod. (l) HE and Masson staining demonstrate the histopathological changes in pancreatic tissues of CP mouse models following intraperitoneal administration of neutrophil-depleting anti-Ly6G antibody compared with isotype control antibody.

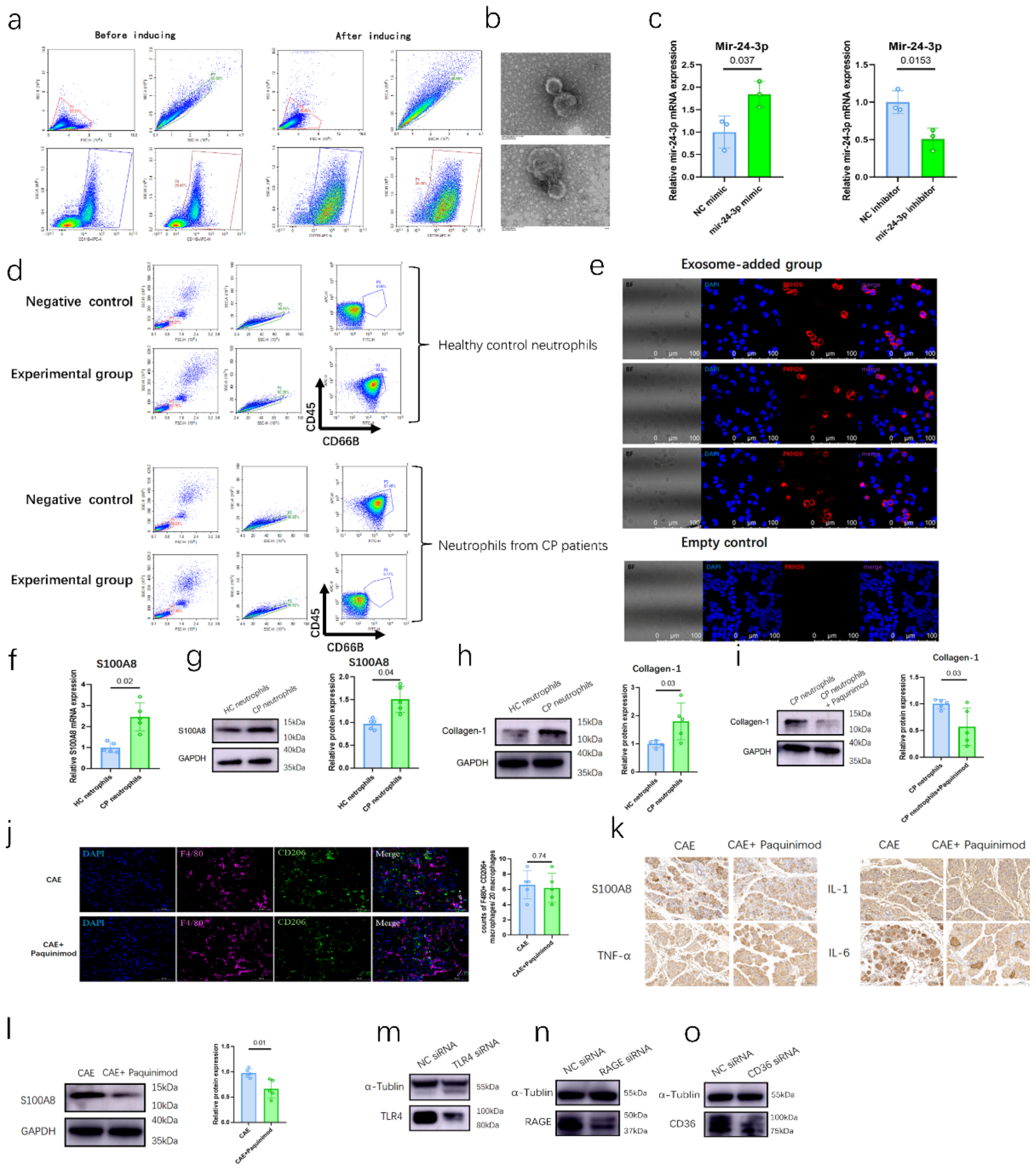

Figure S6. (a) The representative flow cytometry imagines and CD11B expression of HL-60 cell line before neutrophil inducing or after neutrophil inducing. (b) A representative electron microscope image of used exosomes in neutrophil uptake assay. (c) The expression of miR-24-3p between NC group and miR-24-3p mimic group or miR-24-3p inhibitor group. (d) The representative flow cytometry imagines and CD66B and CD45B expression of sorted neutrophils from healthy control individuals and chronic pancreatitis patients. (e) The exosome loaded with PKH26 dye and can be absorbed by human neutrophils, including exosome-added group and empty control. (f)The mRNA expression of S100A8 between healthy control (HC) neutrophils and neutrophils from chronic pancreatitis patients (CP). (g) Protein expression of S100A8 between neutrophils from HC and neutrophils from CP. N = 5 per group. (h) Protein expression of collagen-1 in pancreatic stellar cells after coculture with neutrophils from HC and neutrophils from CP. N = 5 per group. (i) Relative protein levels of collagen-1 in pancreatic stellar cells after coculture with CP

neutrophils, with or without paquinimod. N = 5 per group. (j) Left: representative immunofluorescence staining of pancreatic sections showing $CD206^{+}F4/80^{+}$ double-positive macrophages in pancreas of CP mice treated with paquinimod versus vehicle control. Right: Quantification of $CD206^{+}F4/80^{+}$ cells per field N = 5 per group. (k) The representative immunohistochemical imagine of S100A8, TNF-α, IL-1 and IL-6 in pancreas of CP mice treated with paquinimod versus vehicle control. (l) Protein expression of S100A8 between neutrophils from pancreas of CP mice treated with or without paquinimod. N = 5 per group. (m) The protein level of TLR4 between TLR4 siRNA group and NC siRNA group in HPSCs. (n) The protein level of RAGE between RAGE siRNA group and NC siRNA group in HPSCs. (o) The protein level of CD36 between CD36 siRNA group and NC siRNA group in HPSCs.
